# Reactions of the Criegee Intermediate Methyl Vinyl Ketone Oxide with HC(O)OH: Infrared Spectra of the Hydrogen-Transfer Adduct 2‑Hydroperoxybut-3-en-2-yl Formate

**DOI:** 10.1021/acs.jpca.5c06912

**Published:** 2025-12-03

**Authors:** Yu-Lun Hsiao, Yuan-Pern Lee

**Affiliations:** † Department of Applied Chemistry and Institute of Molecular Science, 34914 National Yang Ming Chiao Tung University, 1001, Ta-Hsueh Road, Hsinchu 300093, Taiwan; ‡ Center for Emergent Functional Matter Science, National Yang Ming Chiao Tung University, Hsinchu 300093, Taiwan

## Abstract

Reactions between the Criegee intermediate and HC­(O)­OH exhibit large rate coefficients, contributing significantly to secondary organic aerosol formation in the atmosphere. This study examines the reaction of methyl vinyl ketone oxide [MVKO, (C_2_H_3_)­C­(CH_3_)­OO], a Criegee intermediate generated via isoprene ozonolysis in the atmosphere, with HC­(O)­OH. Product analysis was performed using step-scan Fourier-transform infrared spectroscopy, capturing time-resolved infrared absorption spectra following irradiation at 248 nm of a flowing mixture of (*Z*)-(CH_2_I)­HC=C­(CH_3_)­I/HC­(O)­OH and O_2_ at 298 K and 10–40 Torr. Ten absorption bands near 1726, 1425, 1378, 1310, 1247, 1215, 1170, 1068, 984, and 952 cm^–1^ were assigned to 2-hydroperoxybut-3-en-2-yl formate [HPBF, C_2_H_3_C­(CH_3_)­(OCHO)­OOH], the hydrogen-transfer adduct of MVKO and HC­(O)­OH. Additional weak bands near 1675, 1600, 1432, 1387, 1330, and 1254 cm^–1^ were tentatively attributed to 2-hydroperoxybuta-1,3-diene [HPBD, (C_2_H_3_)­C­(=CH_2_)­OOH], a hydrogen-transfer isomer of MVKO formed via HC­(O)­OH-catalyzed rearrangement. Two further bands near 1733 and 1200 cm^–1^ were tentatively assigned to a complex of HPBD and HC­(O)­OH, with additional features overlapping those of HPBD. Spectral assignments were supported by B3LYP + D3/aug-cc-pVTZ calculations of vibrational wavenumbers and IR intensities. The identification of HPBF and HPBD is consistent with the reaction pathway scheme predicted by the CCSD­(T)/aug-cc-pVTZ//B3LYP + D3/aug-cc-pVTZ method. In contrast to reactions of CH_2_OO or CH_3_CHOO with HC­(O)­OH, no dehydrated end product of the adduct was observed, which was attributed to the lack of an abstractable hydrogen atom in HPBF.

## Graphical abstract

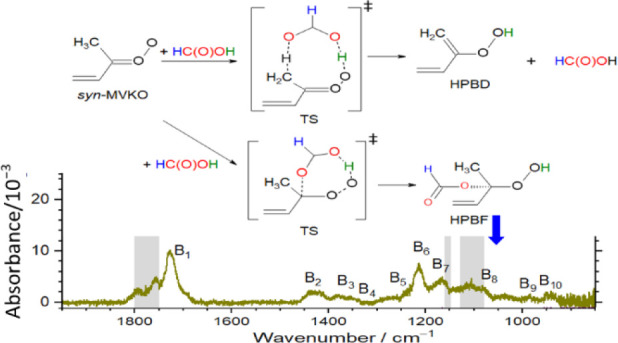

## 1 Introduction

Carbonyl oxides, commonly referred to as Criegee intermediates, play crucial roles in atmospheric chemistry. They contribute to the nonphotolytic production of hydroxyl radicals (OH)^1,2^ and facilitate the formation of secondary organic aerosols.^3,4^ In recent years, research on Criegee intermediates have expanded significantly, as reflected in numerous reviews and perspective articles that highlight their chemical reactivity and atmospheric relevance.^5–15^

Methyl vinyl ketone oxide [MVKO, C_2_H_3_C­(CH_3_)­OO] is a Criegee intermediate formed via ozonolysis of isoprene [CH_2_=CH–C­(CH_3_)=CH_2_],^6,16,17^ which is the most abundantly emitted volatile organic compound after methane.^18,19^ MVKO may be viewed as a derivative of the Criegee intermediate CH_3_CHOO, in which the methine hydrogen atom is replaced by a vinyl group. This substitution introduces resonance stabilization through the vinyl moiety, which enhances the O–O bond and significantly influences the reactivity of MVKO.

MVKO exists in four conformers: *syn*-*trans*, *syn*-*cis*, *anti*-*trans*, and *anti*-*cis*-MVKO. The designations *syn* and *anti* indicate the relative orientation of the methyl group and the terminal O atom, while *cis* and *trans* refer to the spatial arrangement of the terminal C=C bond with respect to the C=O bond. Among them, *syn*-*trans*-MVKO has the lowest energy, with *syn*-*cis*-, *anti*-*trans*-, and *anti*-*cis*-MVKO conformers exhibiting higher energies by ∼7, 11, and 13 kJ mol^–1^, respectively.^20–22^ The molecular structures of the two lowest-energy conformers are illustrated in Figure 1a and b, respectively. Under ambient conditions, interconversion between *syn*- and *anti*-configurations is unlikely due to a substantial barrier (∼125 kJ mol^–1^). In contrast, the interconversion between *cis*- and *trans*-configuration is more feasible, as it involves a significantly smaller barrier (<40 kJ mol^–1^).^20,22^

**Figure 1:**
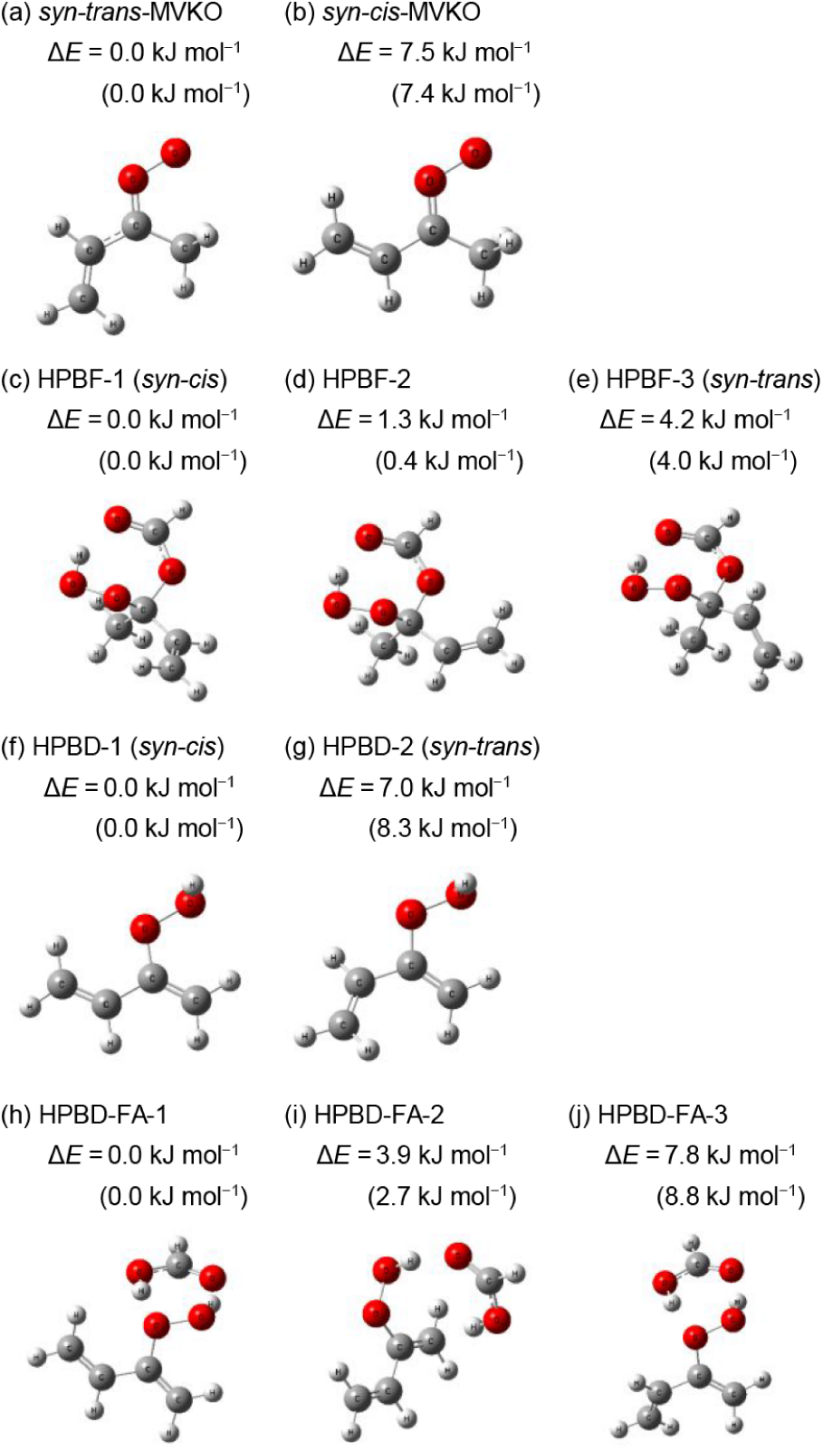
Geometries of conformers of (C_2_H_3_)­C­(CH_3_)­OO (MVKO), (C_2_H_3_)­C­(CH_3_)­(OCHO)­OOH (HPBF), C_2_H_3_C­(=CH_2_)­OOH (HPBD), and complexes of HPBD and HC­(O)­OH (HPBD–FA) considered in this work. (a) *syn-trans-*MVKO, (b) *syn-cis-*MVKO, (c) HPBF-1, (d) HPBF-2, (e) HPBF-3, (f) HPBD-1, (g) HPBD-2, (h) HPBD–FA-1, (i) HPBD–FA-2, and (j) HPBD–FA-3. The structures were computed by the B3LYP + D3/aug-cc-pVTZ method. Relative energies (in kJ mol^–1^) among conformers are listed in parentheses; energies were calculated with the CCSD­(T)/aug-cc-pVTZ//B3LYP + D3/aug-cc-pVTZ method and corrected for B3LYP harmonic vibrational zero-point energy. Energies calculated using the B3LYP + D3/aug-cc-pVTZ method are also given in parentheses for comparison.

To synthesize MVKO in laboratories, Barber et al. developed a novel method involving the ultraviolet (UV) photolysis of gaseous 1,3-diiodo-but-2-ene [(CH_2_I)­HC=C­(CH_3_)­I] in the presence of O_2_.^20^ The photolysis preferentially cleaves the terminal allylic C–I bond rather than the vinylic C–I bond, producing the iodoalkenyl radical, 3-iodo-but-2-en-1-yl [C_2_H_3_C­(CH_3_)­I], as confirmed by IR spectroscopy.^23^ Endo et al. employed Fourier-transform microwave spectroscopy to determine the structure of *syn*-*trans*-MVKO via rotational spectroscopy.^21^ Chung and Lee in our group utilized a step-scan Fourier-transform infrared (FTIR) spectrometer to record transient IR spectra of *syn*-*trans*-MVKO,^23^ while Barber et al. reported the IR spectrum of the CH-stretch overtone region (5750–6300 cm^–1^) of MVKO using IR action spectroscopy coupled with laser-induced fluorescence (LIF) detection of the resulting OH products.^20^ The UV spectrum of MVKO under jet-cooled conditions was characterized by Vansco et al.,^24^ while the spectra of *syn*-MVKO at 298 K were reported by Caravan et al.^25^ and Lin et al.^26^; these authors assumed that, at ambient temperature, *anti*-MVKO decayed rapidly via unimolecular decomposition. Vansco et al. also reported rapid dissociation to O­(^1^D) following electronic excitation of MVKO in the region of 300–430 nm.^24^ However, to the best of our knowledge, no distinct UV spectral features have been identified that differentiate *syn*-MVKO from *anti*-MVKO.

Formic acid, HC­(O)­OH, has an estimated global annual burden of 100–120 Tg, primarily originating from anthropogenic activities, biomass burning, and biogenic emission from vegetation. It has an atmospheric lifetime of 3–4 days.^27,28^ In addition to direct emissions, HC­(O)­OH is also formed through sunlight-driven oxidation of nonmethane hydrocarbons, which contributes substantially to its atmospheric abundance.^27^ As a key contributor to atmospheric acidity, HC­(O)­OH plays an important role in cloud condensation processes and thereby influences the global thermal balance.^29,30^

Reactions between Criegee intermediates and HC­(O)­OH are characterized by large rate coefficients.^31^ Beyond their potential role in the atmospheric removal of Criegee intermediates, incorporating these reactions into global atmospheric models is expected to reduce the predicted lifetime and steady-state concentration of HC­(O)­OH,^31,32^ which might help reconcile the observed atmospheric abundance of formic acid with values predicted by current models.^27^ Moreover, the reaction products formed from Criegee intermediates and HC­(O)­OH are likely to contribute to the formation of secondary organic aerosols (SOA),^33^ further influencing atmospheric composition and climate-relevant processes.

The rate coefficients for the reactions of CH_2_OO + HC­(O)­OH, *syn*-CH_3_CHOO + HC­(O)­OH, and *anti*-CH_3_CHOO + HC­(O)­OH have been reported to be (1.1–1.4) × 10^–10^,^4,31,34^ (2.5 ± 0.3) × 10^–10^,^31^ and (5 ± 3)×10^–10^ cm^3^ molecule^–1^ s^–1^,^31^ respectively. By monitoring the formation of the product, Behera and Lee reported an overall rate coefficient of *syn*-/*anti*-CH_3_CHOO + HC­(O)­OH at 298 K and 40–80 Torr to be (2.1 ± 0.7)×10^–10^ cm^3^ molecule^–1^ s^–1^.^35^

Similar to CH_2_OO, CH_3_CHOO is predicted to react with HC­(O)­OH via a submerged barrier, forming a hydrogen-transfer adduct known as hydroperoxyethyl formate (HPEF), or 1-hydroperoxy-1-formate ethanol, (CH_3_)­CH­(OCHO)­OOH]. However, due to the existence of *syn*- and *anti*-conformation of CH_3_CHOO, the reaction pathway is more complex than that of CH_2_OO + HC­(O)­OH. Cabezas and Endo identified *syn*- and *anti*-HPEF in a discharged jet using microwave spectroscopy.^36^ Behera and Lee employed time-resolved FTIR absorption spectroscopy to detect three types of conformers of HPEF: transient HPEF (P2*/P3*), a more stable open-form HPEF (primarily P2), and a stable intramolecularly hydrogen-bonded HPEF (primarily P1), distinguished by their distinct temporal profiles; the asterisk (*) indicates *syn*-configuration, while the absence of * indicates *anti*-configuration.^35^ The *syn*-HPEF and *anti*-HPEF conformers reported by Cabezas and Endo correspond to P2* and P1, respectively, as identified by Behera and Lee. Based on quantum-chemical computations, Behera and Lee proposed that P2*/P3* was initially produced from the reaction of *syn*-CH_3_CHOO + HC­(O)­OH, while P2/P1 arise from *anti*-CH_3_CHOO + HC­(O)­OH. The conversion of P2*/P3* to P2 and P1 is feasible as the barriers are small. Analogous to the reactions of CH_2_OO + HC­(O)­OH and CH_2_OO + CH_3_C­(O)­OH,^34,37^ the open-form adduct HPEF (P2) undergoes decomposition to yield the dehydrated end-product formic acetic anhydride [CH_3_C­(O)­OC­(O)­H, FAA] with a rate coefficient *k* = (1420 ± 70) s^–1^ at 298 K. In contrast, the intramolecularly hydrogen-bonded HPEF (P1) remains stable.^35^

Additionally, the acid-catalyzed tautomerization of *syn*-CH_3_CHOO via a double hydrogen shift yields vinyl hydroperoxide (VHP, CH_2_=CHOOH).^38^ This transformation is geometrically feasible only for the *syn*-conformer, in which the CH_3_ and OO moieties reside on the same side of the molecule. Liu et al. investigated this reaction using DC­(O)­OD as the acid catalyst by employing vacuum ultraviolet (VUV) photoionization coupled with mass spectrometry. Their detection of partially deuterated VHP (CH_2_=CHOOD) provides compelling evidence in support of the proposed tautomerization mechanism.^39^

The rate coefficient of the reaction of *syn*-MVKO with HCOOH at 10 Torr was reported by Caravan et al. as (3.0 ± 0.1) × 10^–10^ cm^3^ molecule^–1^ s^–1^.^25^ In contrast, no experimental or theoretical rate coefficient has been reported for the reaction of *anti*-MVKO + HC­(O)­OH. MVKO is predicted to react with HCOOH via a submerged transition state through a 1,4-insertion mechanism, forming a hydrogen-transfer adduct, 2-hydroperoxybut-3-en-2-yl formate [HPBF, C_2_H_3_C­(CH_3_)­(OCHO)­OOH]. Structures of the three lowest-energy conformers of HPBF are depicted in Figure 1c–e. This reaction is exothermic by 120–138 kJ mol^–1^,^25,40^ which is smaller than the corresponding exothermicities of 190 kJ mol^–1^ and 170 kJ mol^–1^ for CH_2_OO + HCOOH^41–43^ and CH_3_CHOO + HC­(O)­OH,^35^ respectively. The resonance stabilization of reactant MVKO reduces the exothermicity. For *syn*-MVKO, an additional pathway involving formic-acid-catalyzed tautomerization has been proposed, leading to the formation of 2-hydroperoxybuta-1,3-diene [HPBD, C_2_H_3_C­(=CH_2_)­OOH]. Structures of the two lowest-energy conformers of HPBD are shown in Figure 1f and g. This mechanism proceeds via a 9-membered cyclic transition structure, wherein the hydroxy H atom of HC­(O)­OH approaches the terminal O atom of MVKO, while the carbonyl oxygen of HC­(O)­OH attacks a hydrogen atom of the CH_3_ moiety of MVKO.^40^ If provided with sufficient energy, ∼30 kJ mol^–1^ above MVKO + HC­(O)­OH, HPBD may further decompose into 2-oxybuta-1,3-diene (OBD or 1-methylene-2-propen-1-yloxy) radical and OH.^40^ In contrast, for *anti*-MVKO, the formation of the HPBF adduct is predicted to be the dominant pathway, as acid-catalyzed isomerization is sterically hindered due to the large spatial separation between the terminal O atom and the CH_3_ group, as well as the steric hindrance caused by the vinyl group. Vansco et al. investigated the reaction of *syn*-MVKO + DC­(O)­OD using multiplex photoionization mass spectrometry (MPIMS), detecting both the dissociative photoionization products of the deuterated HPBF adduct and the acid-catalyzed hydrogen-transferred tautomer, deuterated HPBD (DPBD). A branching ratio of ∼94:6 was derived under the assumption of equal population of *syn*- and *anti*-MVKO and identical photoionization cross sections.^40^

In our previous studies, we investigated the reactions of HC­(O)­OH with CH_2_OO and CH_3_CHOO using a step-scan Fourier-transform infrared (FTIR) spectrometer, identifying the IR spectra of several conformers of hydrogen-transfer adducts: hydroperoxylmethyl formate (HPMF) and hydroperoxyethyl formate (HPEF), respectively.^34,35^ We also examined the reaction of CH_2_OO with CH_3_C­(O)­OH and observed hydroperoxylmethyl acetate (HPMA).^37^ The open-form conformers of HPMF and HPMA (or HPEF) further underwent dehydrolysis to yield formic anhydride (FAN) and formic acetic anhydride (FAA), respectively, but their intramolecular H-bonded conformers remained stable.

In the present work, we extended this investigation to the reaction of MVKO with HC­(O)­OH and observed the IR spectrum of the hydrogen-transfer adduct, 2-hydroperoxybut-3-en-2-yl formate (HPBF). However, the corresponding dehydrogenated product was not observed. Certain weak IR bands were tentatively attributed to the tautomeric species 2-hydroperoxybuta-1,3-diene (HPBD) and its complex with HC­(O)­OH. The reaction pathway scheme (RPS) and associated mechanisms were also explored.

## 2 Experiments and Computations

Detailed descriptions of transient IR detection experiments have been reported previously.^34,44,45^ A step-scan FTIR spectrometer (Bruker, Vertex 80v) was employed in conjunction with a multipass White cell (volume ∼1370 cm^3^; base path length = 15 cm; effective path length = 3.6 m), which was pumped using a dry screw pump (1600 L min^–1^). Photolysis was initiated using a KrF excimer laser operating at 8 Hz with a pulse energy of ∼216 mJ pulse^–1^ and a beam size of 1.2 × 8.4 cm^2^. The laser beam entered and exited the White cell through two rectangular quartz windows (3 × 12 cm^2^) mounted on its side, propagating nearly perpendicularly to the IR probe beam. The IR probe beam from the FTIR spectrometer was directed into the White cell via an external port and subsequently detected with a liquid-N_2_-cooled HgCdTe (MCT) detector operating at 77 K.

At each scan step during ultraviolet irradiation, both dc- and ac-coupled signals from the detector were amplified and directed to either an external 14-bit analog-to-digital converter (ADC) with a temporal resolution of 4 ns or an internal 24-bit ADC with a resolution of 12.5 μs. These signals were typically averaged over 15 laser shots per scan step. Using the external ADC, 10 000 data points were acquired over a 40-μs window, including ∼4 μs preceding photolysis; four spectra were recorded and averaged under identical experimental conditions to improve the signal-to-noise ratio (SNR). With the internal ADC, 2000 data points were collected over a 25-ms period, and two spectra were averaged. To shorten the overall data acquisition time, undersampling was implemented using appropriate optical filters. For a spectral range of 770–2200 cm^–1^ at a resolution of 1 cm^–1^, 4569 scan steps were completed in ∼150 min. After apodization with a Blackman–Harris three-term function, the spectral full width at half-maximum (FWHM) was 1.28 times the listed instrumental resolution (typically 1 cm^–1^), which assumes a triangular apodization function.^45^ In continuous-scan mode, the scanning mirror was operated at 10 kHz with a resolution of 0.25 cm^–1^, and 400 scans were averaged. During data acquisition in these continuous-scan experiments, the KrF laser was operated at 2 Hz to irradiate the flowing mixture.

Criegee intermediate MVKO [C_2_H_3_C­(CH_3_)­OO] was generated via photolysis of a flowing mixture of (*Z*)-(CH_2_I)­HC=C­(CH_3_)I and O_2_ at 248 nm. A stream of gaseous O_2_ was bubbled through a dark flask containing liquid (*Z*)-(CH_2_I)­HC=C­(CH_3_)I at 298 K to transport its vapor into the reaction chamber. The partial pressure of (*Z*)-(CH_2_I)­HC=C­(CH_3_)I was determined by integrating the IR absorbance over the 1118–1205 cm^–1^ region and applying a calculated IR cross section of 1.7 × 10^–17^ cm^2^ molecule^–1^.^46^ Separately, another stream of gaseous O_2_ was bubbled through a flask containing liquid HC­(O)­OH to introduce its vapor into the reactor. The partial pressure of HC­(O)­OH was evaluated from the integrated absorbance in the 1050–1085 cm^–1^ region using a reported IR intensity of 5.83 × 10^–18^ cm molecule^–1^.^47^

Using the UV absorption cross-section of (*Z*)-(CH_2_I)­HC=C­(CH_3_)I (∼1.94 × 10^–17^ cm^2^ molecule^–1^ at 248 nm^48^) and the measured laser fluence, the fraction of photolysis of (*Z*)-(CH_2_I)­HC=C­(CH_3_)I was estimated to be ∼46%, assuming a quantum yield of unity. The main stream of O_2_ was directly introduced into the reaction chamber, serving as both the reactant and buffer gas. The total flow rate of O_2_ (*F*_O2_) was ∼12 STP cm^3^ s^–1^ at standard temperature and pressure (STP; 273 K, 1 bar), with total pressure *P*_T_ = 10–40 Torr. The partial pressures of (*Z*)-(CH_2_I)­HC=C­(CH_3_)I and HC­(O)­OH were maintained at 15–25 mTorr and 10–55 mTorr, respectively. The formation of the dimer of formic acid [HC­(O)­OH]_2_ was estimated using the equilibrium constant *K*_eq_ for the association between HC­(O)­OH and *cyc*-[HC­(O)­OH]_2_, defined as *K*_eq_ = (*P*_[HC(O)OH]2_/*P*°)/(*P*_HC(O)OH_/*P*°)^2^ = 361 ± 45, where *P*_[HC(O)OH]2_ and *P*_HC(O)OH_ are the equilibrium partial pressures of *cyc*-[HC­(O)­OH]_2_ and HC­(O)­OH, respectively, and *P*° is the standard pressure (1 bar).^49–52^ Based on this equilibrium, at the highest HC­(O)­OH partial pressure used, *P*_HC(O)OH_ = 55 mTorr, the estimated partial pressure of the dimer was *P*_(HC(O)OH)2_ = 1.4 mTorr, corresponding to ∼2.5% of P_HC(O)OH_. (*Z*)-(CH_2_I)­HC=C­(CH_3_)I (>95%, Accela ChemBio), HC­(O)­OH (Thermo, 98+%), and O_2_ (99.99%, Chiah–Lung) were used as received.

Quantum-chemical calculations were performed using the Gaussian 16 program^53^ to investigate the reaction between MVKO and HC­(O)­OH. Equilibrium geometries, harmonic and anharmonic vibrational wavenumbers, IR intensities, and rotational parameters of all species considered in this work were computed with the B3LYP density functional theory^54–56^ in conjunction with Dunning′s correlation-consistent polarized valence triple-ζ basis set, augmented with diffused and polarization functions (aug-cc-pVTZ).^57,58^ A reaction pathway scheme (RPS) was constructed by identifying transition structures (TS) and confirming their connectivity to reactants and products via intrinsic reaction coordinate (IRC) calculations. The interconversion between various isomers of HPBF and HPBD was explored through potential-energy surface scans, wherein the dihedral angle was varied in 10° increments, while all other geometrical parameters were optimized. Single-point energies of each conformer were further refined at the B3LYP-optimized geometries using the CCSD­(T) method (coupled cluster with single and double excitations and perturbative triples).^59^ Zero-point vibrational energy (ZPVE) corrections were applied based on B3LYP calculations. Harmonic vibrational wavenumbers and IR intensities were evaluated at the global minimum for each species. To improve agreement with experimental data, harmonic vibrational wavenumbers were scaled as discussed in Section 3.1. Anharmonic vibrational wavenumbers and intensities were obtained using second-order vibrational perturbation (VPT2), as implemented in Gaussian 16.

## 3 Results and Discussion

### 3.1 Quantum-Chemical Calculations

In this section, we consider only the reactions of HC­(O)­OH with *syn*-MVKO, excluding *anti*-MVKO, because the reactions of *anti*-MVKO + HC­(O)­OH have been reported by Vansco et al.; they are similar to those of *syn*-MVKO + HC­(O)­OH.^40^ Formic acid exists as *cis* and *trans* conformers, with the *cis-*conformer being ∼16 kJ mol^–1^ greater in energy and separated from the *trans* form by an isomerization barrier of ∼58 kJ mol^–1^.^60^ Given the negligible population of *cis*-HC­(O)­OH at 298 K, only *trans-*HC­(O)­OH was included in the RPS. Throughout this paper, HC­(O)­OH refers to *trans*-HC­(O)­OH unless otherwise stated.

The RPS for reactions of *syn-trans*-MVKO + HC­(O)­OH and *syn*-*cis*-MVKO + HC­(O)­OH are presented in Figure 2; blue and red dashed lines denote reactions involving *syn-trans*- and *syn*-*cis*-MVKO, respectively. All energies are reported relative to the *syn-trans*-MVKO + HC­(O)­OH reactants and were calculated at the CCSD­(T)/aug-cc-pVTZ//B3LYP/aug-cc-PVTZ level of theory. The RPS for reactions of various conformers of MVKO with HC­(O)­OH have previously been reported by Caravan et al.^25^ and Vansco et al.^40^; corresponding values for *syn*-*trans*- and *syn*-*cis*-MVKO + HC­(O)­OH reported by Vansco et al., using the CCSD­(T)-F12/CBS level of theory with an estimate of the CCSDT­(Q) correction, are provided in parentheses for comparison. The reaction pathways for *syn*-*cis*-MVKO + HC­(O)­OH are qualitatively similar to those of *syn-trans*-MVKO + HC­(O)­OH, differing primarily in relative energies. Reactions involving *syn-trans*-MVKO + HC­(O)­OH (blue dashed lines) proceed mainly via three distinct channels: (1) formation of a hydrogen-transfer adduct HPBF-3 [C_2_H_3_(CH_3_)­C­(OCHO)­OOH] with an exothermicity of ∼125 kJ mol^–1^ via a submerged transition structure TS1 (−62 kJ mol^–1^ relative to *syn*-*trans*-MVKO + HC­(O)­OH),; (2) HC­(O)­OH-assisted double hydrogen-transfer tautomerization yields a hydrogen-bonded complex of HPBD with HC­(O)­OH (HPBD–FA-4), with an exothermicity of 98 kJ mol^–1^ via a transition structure TS2 (energy −23 kJ mol^–1^); and (3) direct hydrogen transfer from *syn*-*trans*-MVKO to HPBD-2 via TS4 (energy 72 kJ mol^–1^). This direct hydrogen-transfer channel cannot compete with the catalytic channel because of the large barrier. The scheme of the first two reaction paths is shown in Figure 3. The HC­(O)­OH-catalyzed isomerization proceeds through a nine-membered cyclic transition structure, wherein the hydroxy H atom of HC­(O)­OH approaches the terminal O atom of MVKO and the carbonyl oxygen of HC­(O)­OH abstracts a hydrogen atom of the methyl group of MVKO. This concerted mechanism lowers the barrier for hydrogen transfer from ∼72 kJ mol^–1^ above *syn*-*trans*-MVKO + HC­(O)­OH to ∼23 kJ mol^–1^ below it. The first two reaction channels involving *syn*-*trans*-MVKO + HC­(O)­OH proceed via a prereaction complex (PRC1) located at −64 kJ mol^–1^. The hydrogen-bonded complex HPBD-FA-4 subsequently decomposes to HPBD-2 + HC­(O)­OH, requiring ∼43 kJ mol^–1^. Further decomposition of HPBD-2 to *trans*-OBD [2-oxybuta-1,3-diene, C_2_H_3_C­(=CH_2_)­O] + OH requires an additional 80 kJ mol^–1^.

**Figure 2:**
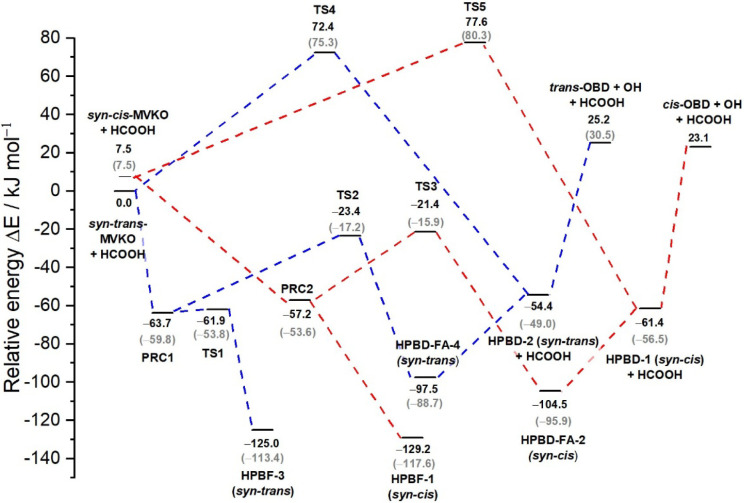
Reaction pathway scheme (RPS) of *syn-*MVKO + HC­(O)­OH predicted with the CCSD­(T)/aug-cc-pVTZ//B3LYP + D3/aug-cc-PVTZ method. Energies were corrected for zero-point vibrational energies calculated with the B3LYP + D3/aug-ccpVTZ method. Dashed lines in blue and red indicate reactions of HC­(O)­OH with *syn-trans*-MVKO and *syn-cis*-MVKO, respectively. Energies reported by Vansco et al.^40^ using the CCSD­(T)-F12/CBS level of theory with an estimate of the CCSDT­(Q) correction are listed in parentheses for comparison. HPBF indicates (C_2_H_3_)­C­(CH_3_)­(OCHO)­OOH, HPBD indicates C_2_H_3_C­(=CH_2_)­OOH, HPBD–FA indicates complexes of HPBD and HC­(O)­OH, OBD indicates C_2_H_3_C­(=CH_2_)­O, and PRC indicates a prereaction complex.

**Figure 3:**
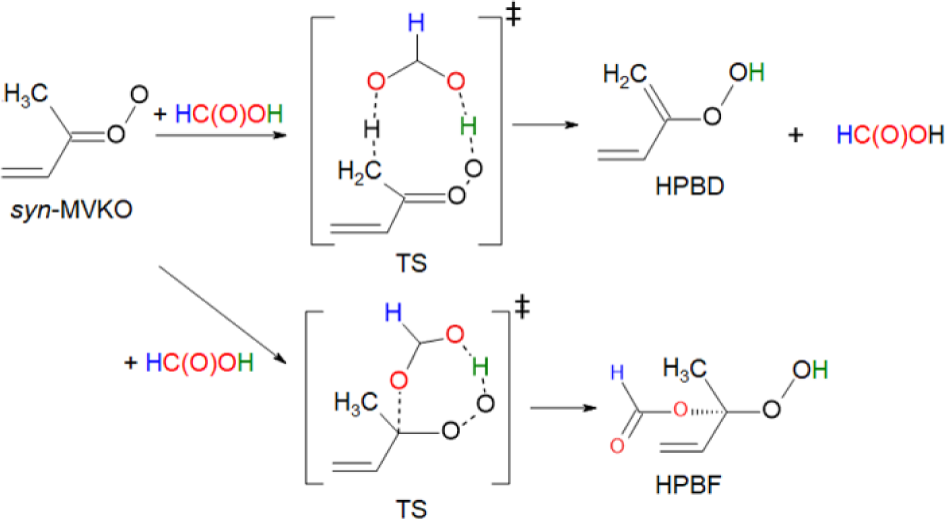
Reaction schemes for *syn*-MVKO + HC­(O)­OH. The hydrogen atoms in HC­(O)­OH are color-coded.

The *syn-cis*-MVKO + HC­(O)­OH reactions (red dashed lines) followed analogous pathways. The exothermicities for the formation of HPBF-1, HPBD–FA-2, and HPBD-1 + HC­(O)­OH are approximately 137, 112, and 69 kJ mol^–1^, respectively. The transition structures associated with the latter two channels lie at – 29 (TS3) and 70 (TS5) kJ mol^–1^ relative to *syn-cis*-MVKO + HC­(O)­OH, which itself is 7.5 kJ mol^–1^ higher in energy than *syn-trans*-MVKO + HC­(O)­OH. The first two channels proceed via a prereaction complex (PRC2), located at −65 kJ mol^–1^ relative to *syn-cis*-MVKO + HC­(O)­OH. The complex HPBD–FA-2 decomposes to HPBD-1 (*syn*-*cis*-) + HC­(O)­OH with an energy requirement of 43 kJ mol^–1^, and further decomposition of HPBD-1 to *cis*-OBD + OH requires an additional 85 kJ mol^–1^.

Eleven conformers of HPBF (HPBF-1–HPBF-11) were identified; their optimized structures and relative energies are shown in Figure S1, with Cartesian coordinates provided in Table S1. Among these, the intramolecularly H-bonded conformers HPBF-1–HPBF-3, shown in Figure 1c–e, exhibit the lowest relative energies (0.0, 1.3, and 4.2 kJ mol^–1^, respectively). Detailed structural parameters for these conformers are presented in Figure S2. The remaining conformers adopt open structures and lie >7.9 kJ mol^–1^ above HPBF-1.

Two conformers of HPBD (HPBD-1 and HPBD-2) were located with an energy separation of 7.0 kJ mol^–1^. Their optimized structures and relative energies are shown in Figure 1f and g, with Cartesian coordinates listed in Table S2; detailed structural parameters are presented in Figure S3. HPBD forms hydrogen-bonded complexes with HC­(O)­OH, designated as HPBD–FA. Fifteen conformers of HPBD and formic acid (HPBD–FA-1 to HPBD–FA-15) were identified; their optimized structures and relative energies are shown in Figure S4, and Cartesian coordinates are listed in Table S3. The first three conformers (Figure 1h–j) lie within 7.8 kJ mol^–1^ of each other, and their structural parameters are detailed in Figure S5. Optimized structures of PRC1, PRC2, and transition structures TS1–TS5 are shown in Figure S6, with the corresponding Cartesian coordinates provided in Table S4.

The potential energy profiles for interconversion among the three lowest-energy conformers of HPBF via rotation of the dihedral angle Φ (C1–C2–C3–O1) are shown in Figure S7. The energy barriers for interconversion among HPBF-1, HPBF-2, and HPBF-3 are below 12 kJ mol^–1^. Other conformers can convert to HPBF-1–HPBF-3 through pathways with barriers of less than 40 kJ mol^–1^. Given the highly exothermic nature of HPBF formation (125–137 kJ mol^–1^), it is likely that internally excited conformers of HPBF possess sufficient energy to overcome these barriers, resulting in a Boltzmann distribution among conformers. Analogously, the conversion of *syn*-*trans*-HPBD to the lowest-energy *syn*-*cis*-HPBD occurs via a barrier of ∼11 kJ mol^–1^, as illustrated in Figure S8.

The scaled harmonic vibrational wavenumbers, along with IR intensities, of the low-energy conformers of HPBF, HPBD, and HPBD-FA are summarized in Tables S5–S7, respectively. Anharmonic vibrational calculations were also performed for HPBF and HPBD, as listed in Tables S5 and S6, respectively. Scaled harmonic vibrational wavenumbers of all species discussed in this work were obtained using the linear scaling equation *y* = 0.971*x* + 11.0, in which *y* is the scaled vibrational wavenumber and *x* is the calculated harmonic vibrational wavenumber. This equation was derived by fitting experimentally observed wavenumbers in the range of 800–1800 cm^–1^ to harmonic vibrational wavenumbers computed at the B3LYP/aug-cc-pVTZ level for the precursor (*Z*)-(CH_2_I)­HC=C­(CH_3_)­I, as illustrated in Figure S9 and tabulated in Table S8. The average absolute deviation between experiments and scaled harmonic vibrational wavenumbers of (*Z*)-(CH_2_I)­HC=C­(CH_3_)I is (8.3 ± 5.1) cm^–1^ and that between experiments and anharmonic vibrational wavenumbers is (11.1 ± 5.3) cm^–1^.

### 3.2 Time-Resolved IR Spectra Following Irradiation of (*Z*)-(CH_2_I)­HC= C­(CH_3_)­I/HC­(O)­OH/O_2_ Mixtures

Time-resolved IR spectra were recorded with either an external ADC (resolution 4 ns, covering 0–38 μs) or an internal ADC (resolution 12.5 μs, covering 0–25 ms). Results recorded with the external ADC provide information on the initial products, while those recorded with the internal ADC provide data with an improved signal-to-noise ratio but might suffer from contributions from products of secondary reactions. Different data processing methods are, hence, employed.

Figure 4a displays the IR absorption spectrum recorded in the region 850–1950 cm^–1^ (instrumental resolution 1 cm^–1^) for a flowing mixture of (*Z*)-(CH_2_I)­HC=C­(CH_3_)­I/HC­(O)­OH/O_2_ (0.025/0.038/40.0, total pressure *P*_T_ = 40.1 Torr) at 298 K. Five characteristic bands of (*Z*)-(CH_2_I)­HC=C­(CH_3_)I (indicated by asterisks), with the most intense near 1169 cm^–1^, and two bands of HC­(O)­OH near 1105 and 1775 cm^–1^ (marked with triangles) were observed. For comparison, the reference spectrum of MVKO reported by Chung and Lee^23^ is shown in Figure 4b, with bands of MVKO indicated by blue circles. These spectra are reproduced in Figures S10a and b. Time-resolved difference spectra, recorded using an external ADC at intervals of 0–5, 5–10, 10–15, and 30–35 μs following photolysis at 248 nm, are presented in Figure S10c–f, respectively; negative features corresponding to the depletion of reactants were truncated, while positive features represent product formation. Immediately after photolysis, absorption bands of (*Z*)-(CH_2_I)­HC=C­(CH_3_)I and HC­(O)­OH diminished, and bands of MVKO were not distinctly observed, likely due to its rapid reaction with HC­(O)­OH. After 5 μs, product bands became discernible. To minimize spectral interference from negative reactant bands, we reconstructed the spectra by reintroducing (adding back) the depleted signals of (*Z*)-(CH_2_I)­HC=C­(CH_3_)I and HC­(O)­OH using appropriately scaled reference spectra.

**Figure 4:**
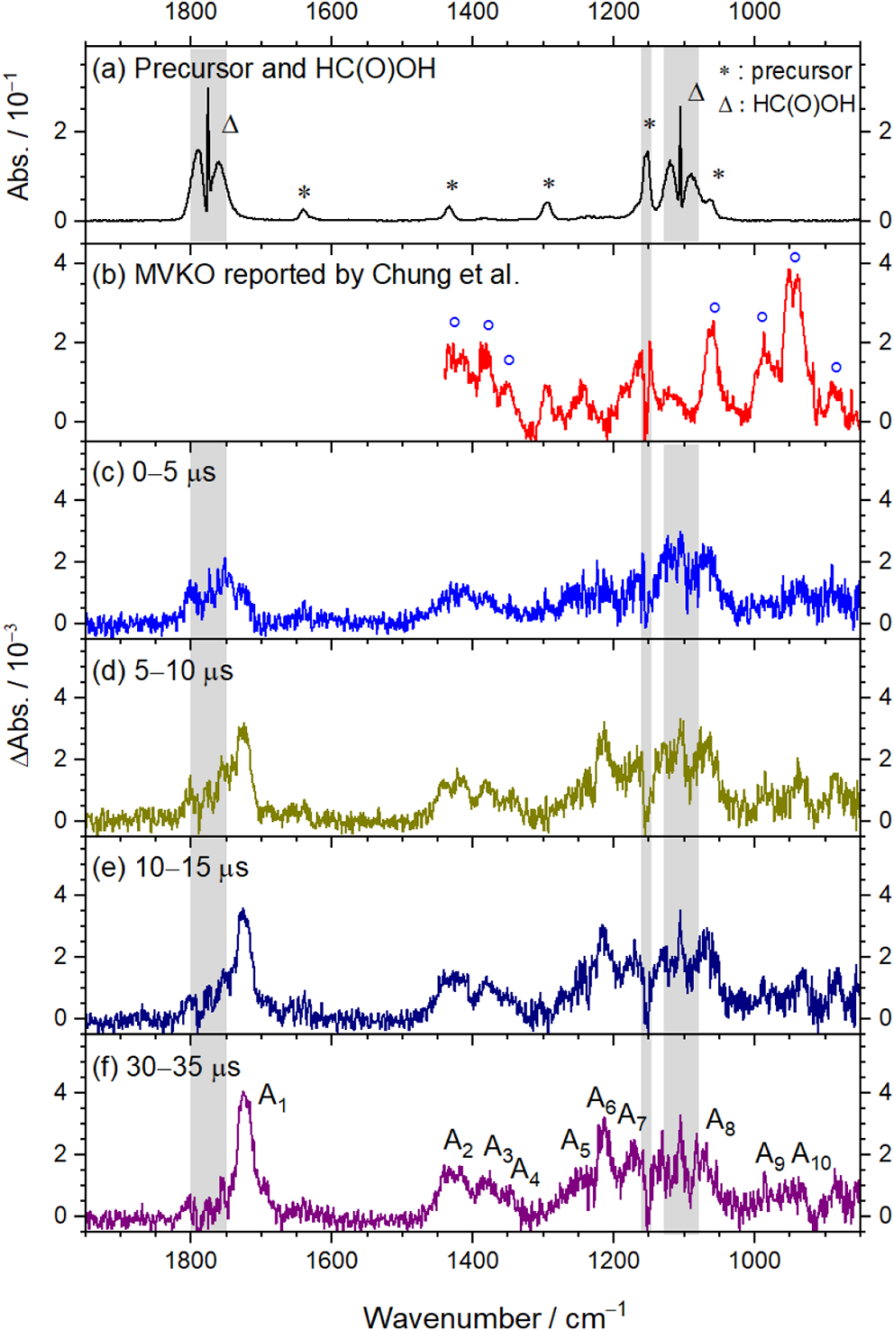
IR spectra recorded with an external ADC following photolysis at 248 nm of a flowing mixture of (*Z*)-(CH_2_I)­HC=C­(CH_3_)­I/HC­(O)­OH/O_2_ (0.025/0.038/40.0, *P*_T_ = 40.1 Torr) at 298 K. (a) Absorption spectrum before photolysis. Bands of (*Z*)-(CH_2_I)­HC=C­(CH_3_)I are indicated by *, while those of HC­(O)­OH are by Δ. (b) MVKO absorption spectrum reported by Chung and Lee.^23^ Processed difference spectra were recorded 0–5 μs (c), 5–10 μs (d) 10–15 μs (e), and 30–35 μs (f) after irradiation; depletion of absorption bands of the precursors (*Z*)-(CH_2_I)­HC=C­(CH_3_)I and HC­(O)­OH were added back. Regions affected by precursor absorption are shaded gray. New features in groups A are labeled A_1_–A_10_ in (f). Instrumental resolution is 1.0 cm^–1^.

The processed time-resolved spectra recorded at 0–5, 5–10, 10–15, and 30–35 μs following photolysis at 248 nm are shown in Figure 4c–f, respectively. Regions where intense absorption bands of (*Z*)-(CH_2_I)­HC=C­(CH_3_)I and HC­(O)­OH may interfere are shaded gray. Ten features near 1726, 1425, 1378, 1310, 1247, 1215, 1170, 1068, 984, and 952 cm^–1^ (designated A_1_ through A_10_, respectively) exhibited increasing intensity over the reaction period. These bands in group A correspond to the absorption of HPBF, which is discussed in Section 4.1.

To extend the observation temporal window and enhance the signal-to-noise ratio, a separate experiment employed an internal 24-bit ADC with a temporal resolution of 12.5 μs. Figure S11a displays the IR absorption spectrum in the region 850–1950 cm^–1^ (resolution 2 cm^–1^) for a flowing mixture of (*Z*)-(CH_2_I)­HC=C­(CH_3_)­I/HC­(O)­OH/O_2_ (0.037/0.055/40.0, *P*_T_ = 40.1 Torr) at 298 K. Absorption bands of (*Z*)-(CH_2_I)­HC=C­(CH_3_)I and HC­(O)­OH are marked with asterisks and triangles, respectively. Time-resolved difference spectra recorded at 0–50, 50–100, and 150–1000 μs after irradiation at 248 nm are shown in Figure S11b–d, respectively. During this period, the formation of methyl vinyl ketone (MVK) becomes non-negligible. For reference, the IR spectrum of methyl vinyl ketone (MVK) is provided in Figure S11e. These spectra were processed analogously by adding back the depleted signal of reactants (*Z*)-(CH_2_I)­HC=C­(CH_3_)I and HC­(O)­OH and, additionally, subtracting the contributions from MVK. The resulting spectra are shown in Figure S12. The positions and intensities of the newly observed features closely resemble those in Figure 4, labeled as A_1_–A_10_. Notably, the intensities of these features remain nearly constant throughout the reaction period, up to 1 ms.

Additional experiments conducted at 10 Torr using both external and internal ADCs yielded spectral features postphotolysis that are nearly identical to those observed at 40 Torr, indicating negligible pressure dependence under the conditions examined.

### 3.3 Continuous-Scan IR Spectra during Irradiation of (*Z*)-(CH_2_I)­HC=C­(CH_3_)­I/O_2_/HC­(O)­OH Mixtures

The continuous-scan mode provides data with the best signal-to-noise ratio, but it can only detect IR absorption of end products without temporal resolution. To distinguish the differences in products at various pressures, the processed spectra of mixtures of (*Z*)-(CH_2_I)­HC=C­(CH_3_)­I/HC­(O)­OH/O_2_ at 40.8 and 10.7 Torr in the region 850–1950 cm^–1^ recorded during UV irradiation using FTIR in continuous-scan mode at an instrumental resolution of 0.25 cm^–1^ are presented in Figure 5b and c, respectively. To indicate possible regions of interference due to parent absorption, the IR spectrum of a flowing mixture of (*Z*)-(CH_2_I)­HC=C­(CH_3_)­I/HC­(O)­OH/O_2_ (0.032/0.039/40.7, *P*_T_ = 40.8 Torr) prior to photolysis is shown in Figure 5a; bands of (*Z*)-(CH_2_I)­HC=C­(CH_3_)I are indicated by asterisks, while bands of HC­(O)­OH are marked with triangles. The processed spectra were derived from the raw spectra described below. The spectrum in Figure 5a is also shown in Figure S13a. Difference spectra obtained during irradiation at 248 nm (8 Hz, 216 mJ pulse^–1^) of the flowing mixture are presented in Figure S13b. The processed spectrum, in which the decays of (*Z*)-(CH_2_I)­HC=C­(CH_3_)I and HC­(O)­OH were added back, is shown in Figure S13c. Because the reactions in the system are complex during a longer reaction period, we could only compare the results with and without HC­(O)­OH added and ascribe the differences to reactions of MVKO with HC­(O)­OH. For comparison, Figure S13d presents the spectrum recorded during irradiation at 248 nm of a flowing mixture of (*Z*)-(CH_2_I)­HC=C­(CH_3_)­I/O_2_ (0.032/40.7, *P*_T_ = 40.7 Torr) without HC­(O)­OH. The processed spectrum in which the loss of (*Z*)-(CH_2_I)­HC=C­(CH_3_)I was added back is presented in Figure S13e. By subtracting 0.50× Figure S13e from Figure S13c, we isolated new spectral features attributed to products from reactions of MVKO with HC­(O)­OH, as shown in Figure S13f. Analogous experiments conducted at 10.7 Torr were processed using the same approach, and the resulting features are presented in Figure S14f. The spectra obtained at 10 and 40 Torr exhibit only minor differences.

**Figure 5:**
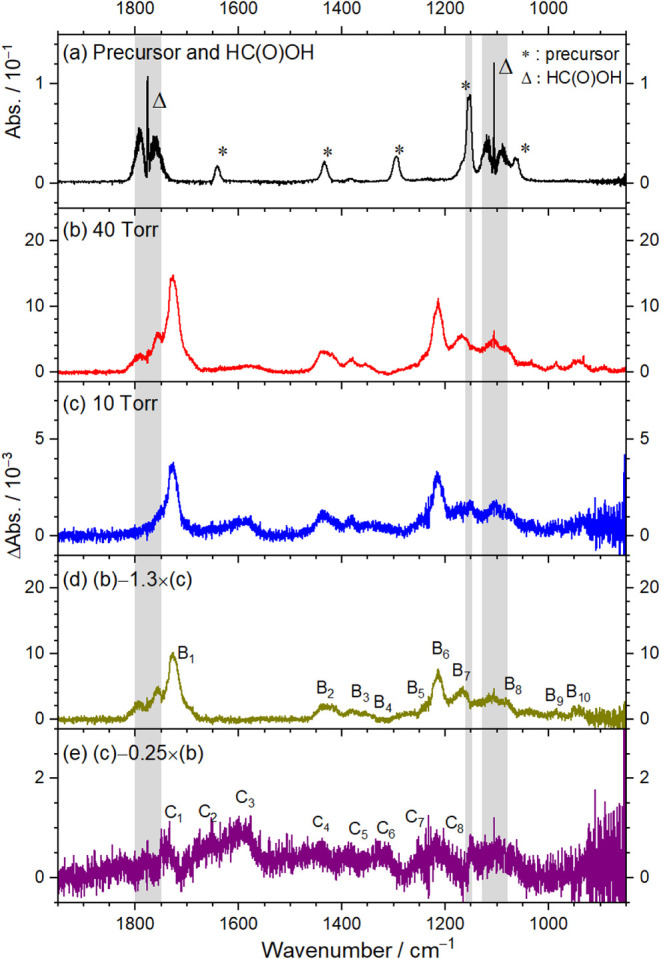
IR spectra recorded with continuous-scan mode during photolysis at 248 nm of a flowing mixture of (*Z*)-(CH_2_I)­HC=C­(CH_3_)­I/HC­(O)­OH/O_2_ at 298 K. (a) Absorption spectrum before photolysis. (b) Processed spectra of a flowing mixture of (*Z*)-(CH_2_I)­HC=C­(CH_3_)­I/HC­(O)­OH/O_2_ (0.032/0.039/40.7, *P*_T_ = 40.8 Torr) during photolysis, from Figure S13f. (c) Processed spectra of a flowing mixture of (*Z*)-(CH_2_I)­HC=C­(CH_3_)­I/HC­(O)­OH/O_2_ (0.015/0.011/10.7, *P*_T_ = 10.7 Torr) during photolysis, from Figure S14f. (d) Difference spectrum obtained from the spectrum in (b) minus 1.3 times that in (c). (e) Difference spectrum obtained from the spectrum in (c) minus 0.25 times that in (b). Regions affected by precursor absorption are shaded gray. New features in groups B and C are labeled B_1_–B_10_ in (d) and C_1_–C_8_ in (e). Instrumental resolution is 0.25 cm^–1^.

To distinguish the minor differences, the processed spectra of a mixture at 40.8 Torr were recorded during UV irradiation (Figure S13f) is reproduced in Figure 5b. A corresponding processed spectrum for a similar mixture at 10.7 Torr is shown in Figure S14f and is reproduced in Figure 5c. Compared to the spectrum at 40.8 Torr, the 10.7 Torr spectrum exhibits several additional weak features. To isolate the spectral features unaffected by these weak contributions, we subtracted 1.3× Figure 5c from Figure 5b. The factor of 1.3 was derived by observing a zero baseline in the spectral region 1500–1650 cm^–1^, indicating no contribution from bands in group C. The resulting spectrum, free from interference of these additional weaker features, is presented in Figure 5d; the newly resolved features are labeled B_1_–B_10_. Conversely, subtracting 0.25× Figure 5b from Figure 5c yielded a spectrum highlighting the additional weak features observed at 10.7 Torr; these are shown in Figure 5e and marked as C_1_–C_8_. The factor of 0.25 was derived by observing the disappearance of the intense band B_1_, indicating no significant contribution from bands in group B.

## 4 Discussion

Although the unimolecular decay rate coefficient of *anti*-MVKO has been predicted to be large, in the range of 2140–64000 s^–1^,^14,20,25^ reactions of MVKO with HC­(O)­OH in our experiments might be highly competitive, considering an expected bimolecular rate coefficient of >3 × 10^–10^ cm^3^ molecule^–1^ s^–1^ and [HC­(O)­OH] ≈ 10^15^ molecules cm^–3^. However, no spectral characterization (UV, IR, or microwave) of *anti*-MVKO has been reported. Even though we observed an IR spectrum mainly contributed by *syn*-MVKO,^23^ we could not exclude the possibility that *anti*-MVKO was also present in the system and participated in the reaction with HC­(O)­OH. In the following discussion, we used the RPS of *syn*-MVKO + HC­(O)­OH, but similar contributions from *anti*-MVKO + HC­(O)­OH might apply.

### 4.1 Assignments of Bands in Groups A/B to Conformers of HPBF

Reported quantum-chemical calculations,^25,40^ along with our calculation results (Figure 2), indicate that *syn*-MVKO reacts with HC­(O)­OH via a pathway involving a submerged transition structure, located approximately 54–62 kJ mol^–1^ below the energy of *syn*-MVKO + HC­(O)­OH. This reaction proceeds through a 1,4-insertion mechanism, yielding a hydrogen-transfer adduct, 2-hydroperoxybut-3-en-2-yl formate [HPBF, C_2_H_3_C­(CH_3_)­(OCHO)­OOH], as the primary product.

Among the 11 predicted conformers of HPBF, only three lie within 5 kJ mol^–1^ of the least-energy conformer, HPBF-1. Conformers HPBF-4 to HPBF-11 are at least 8 kJ mol^–1^ higher in energy than HPBF-1 and are therefore excluded from consideration. The reaction of *syn*-*trans*-MVKO with HC­(O)­OH is predicted to yield HPBF-3 directly, while the reaction of *syn*-*cis*-MVKO + HC­(O)­OH leads to HPBF-2. These conformers are calculated to interconvert via barriers below 12 kJ mol^–1^ (Figure S7), suggesting a facile equilibration. Accordingly, we focus our analysis on HPBF-1, HPBF-2, and HPBF-3.

The IR features in group A (obtained from step-scan, Figure S12d) and B (obtained from continuous scan, Figure 5d) are reproduced in Figure 6a and b, respectively. The spectral features in groups A and B exhibit nearly the same pattern, although the latter benefits from an improved signal-to-noise ratio. We hence treat them as the same group and used the spectral positions of features in group B to compare with theoretical simulations. For comparison, simulated IR spectra of the HPBF conformers HPBF-1, HPBF-2, and HPBF-3 are shown in Figure 6c–e, respectively. These IR spectra were simulated based on scaled harmonic fundamental vibrational wavenumbers, harmonic IR intensities predicted at the B3LYP/aug-cc-pVTZ level of theory, and a Gaussian width (FWHM) of 5 cm^–1^. An analogous comparison of observed spectra with stick spectra derived from anharmonic vibrational wavenumbers is presented in Figure S15; overtone and combination bands (indicated in lighter colors) generally exhibit low intensity, except for a few that are located near the fundamental bands, so they do not introduce significant additional features beyond the spectral width of observed bands.

**Figure 6:**
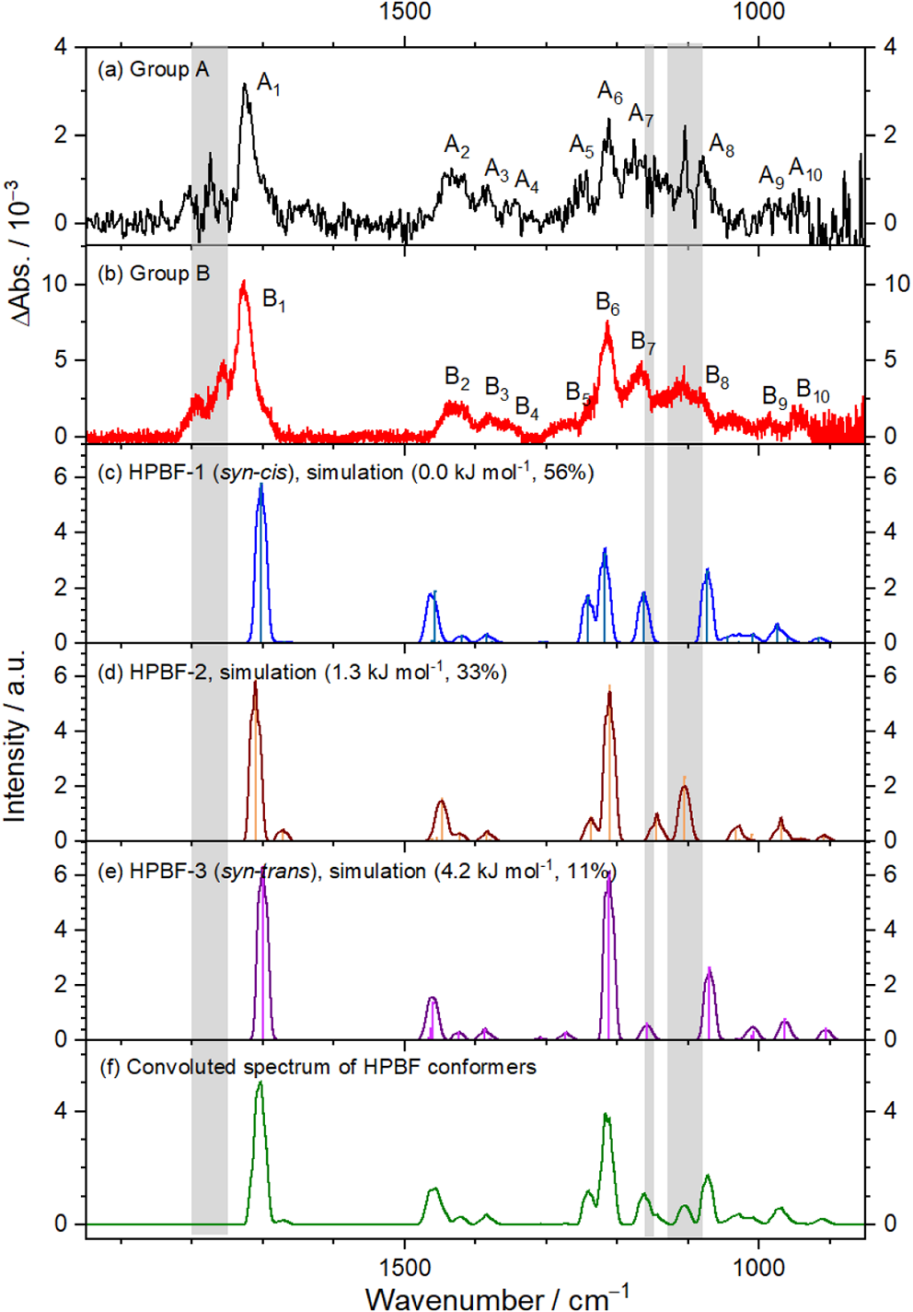
Comparison of bands in groups A and B with simulated scaled harmonic spectra of the three lowest-energy conformers of HPBF, (C_2_H_3_)­C­(CH_3_)­(OCHO)­OOH. (a) Absorption spectrum of group A, taken from Figure S12d with an instrumental resolution of 2 cm^–1^. (b) Absorption spectrum of group B, taken from Figure 5d with an instrumental resolution of 0.25 cm^–1^. Simulated spectra of fundamental bands of HPBF-1 (c), HPBF-2 (d), and HPBF-3 (e) based on scaled harmonic vibrational wavenumbers and IR intensities predicted with the B3LYP + D3/aug-cc-pVTZ method. Gaussian fwhm is 5 cm^–1^. (f) Convoluted spectrum of HPBF-1 to HPBF-3 according to Boltzmann population distribution 0.58:0.33:0.11. Regions affected by precursor absorption are shaded gray.

Simulated IR spectra of conformers HPBF-1, HPBF-2, and HPBF-3 reveal generally consistent features, with some distinctions in the region of 1100–1250 cm^–1^. In this region, the three bands (ν_18_–ν_20_) of HPBF-1 display comparable intensities, whereas the ν_19_ band of HPBF-2 and HPBF-3 is markedly more intense than ν_18_ and ν_20_. A convoluted spectrum, constructed from the simulated spectra of HPBF-1, HPBF-2, and HPBF-3 using their predicted Boltzmann population distributions of 56%, 33%, and 11%, respectively, is shown in Figure 6f. The observed intensities and wavenumbers for features in group A/B align satisfactorily with the individually simulated spectra of HPBF-1, HPBF-2, and HPBF-3; however, the agreement is notably improved when compared with the convoluted spectrum. Given the current spectral resolution and inherent uncertainties in theoretical predictions of band intensities and wavenumbers, unambiguous assignment of individual conformer bands remains highly challenging. Nevertheless, the comparison between observed and simulated spectra strongly suggests that more than one conformer of HPBF contributes to the experimental spectrum.

As summarized in Table 1, the most intense band B_1_, observed at 1726 cm^–1^, closely matches the C=O stretching (ν_9_) modes predicted for HPBF-1 (1702 cm^–1^), HPBF-2 (1710 cm^–1^), HPBF-3 (1700 cm^–1^), and the convoluted HPBF spectrum (1703 cm^–1^). This band is red-shifted relative to the typical C=O stretching wavenumber near 1750 cm^–1^, indicative of an H-bonded C=O group. Such a shift is consistent with the predicted intramolecular H-bonding in the structures of HPBF-1, HPBF-2, and HPBF-3. In our previous studies on the reactions of CH_2_OO + HC­(O)­OH^34^ and CH_3_CHOO + HC­(O)­OH,^35^ not only open-form adducts but also stable intramolecularly H-bonded adducts were observed. The corresponding H-bonded C=O stretching bands of these H-bonded adducts were observed at 1732 and 1724 cm^–1^, respectively, similar to our observation at 1726 cm^–1^.

**Table 1:** Comparison of Observed Vibrational Wavenumbers (in cm^–1^) and Relative IR Intensities of Features in Group A/B with the Scaled Harmonic Vibrational Wavenumbers and IR Intensities of Conformers (C_2_H_3_)­C­(CH_3_)­(OCHO)­OOH (HPBF-1, HPBF-2, and HPBF-3)

	HPBF conformers^a^				
Mode	HPBF-1	HPBF-2	HPBF-3	Convoluted^b^	Experiment
ν_9_	1702^c^ (311)^d^	1710^c^ (291)^d^	1700^c^ (329)^d^	1703 (306)^d^	1726 (100)^e^
ν_10_	1670 (1.9)	1673 (21.4)	1677 (0.3)	1673 (8.2)	
ν_11_	1461 (3.3)	1461 (4.2)	1466 (4.3)	1458 (99.2)	1425 (54)
ν_12_	1457 (101)	1455 (7.0)	1463 (22.1)		
ν_13_	1452 (1.1)	1447 (78.3)	1460 (70.8)		
ν_14_	1420 (14.1)	1423 (15.1)	1424 (15.2)	1422 (14.6)	
ν_15_	1385 (15.9)	1384 (16.2)	1387 (20.0)	1385 (18.4)	1378 (28)
ν_16_	1377 (1.7)	1379 (2.5)	1380 (2.0)		
ν_17_	1305 (0.9)	1306 (0.7)	1308 (3.5)	1308 (2.7)	1310 (4)
ν_18_	1241 (88.3)	1237 (44.5)	1273 (14.6)	1240 (64.1)	1247 (41)
ν_19_	1218 (173)	1211 (284)	1212 (312)	1214 (225)	1215 (60)
ν_20_	1162 (97.2)	1144 (48.1)	1158 (31.4)	1162 (73.8)	1170 (49)
ν_21_		1105 (117)		1105 (38.6)	^f^
	1073 (138)		1070 (138)	1071 (92.5)	1068 (54)
ν_22_	1044 (11.1)	1032 (18.6)	1028 (2.4)	1032 (15.0)	
ν_23_	1028 (1.5)	1028 (1.7)	1010 (9.0)		
ν_24_	1009 (16.4)	1010 (12.5)	1008 (15.9)	1009 (15.1)	
ν_25_	974 (34.7)	969 (38.3)	963 (40.1)	970 (36.5)	984 (8)
ν_26_	959 (7.9)	941 (5.3)	951 (0.5)	959 (6.2)	952 (30)
ν_27_	915 (10.3)	908 (11.5)	906 (21.5)	908 (11.9)	^g^
ν_28_	812 (9.3)	824 (83.7)	814 (62.0)	817 (39.6)	^g^

a Predicted with the B3LYP + D3/aug-cc-pVTZ method.

b Constructed from the simulated spectra of HPBF-1, HPBF-2, and HPBF-3 using their predicted Boltzmann population distribution of 56%, 33%, and 11%, respectively.

c Harmonic vibrational wavenumbers (cm^–1^) scaled with *y* = 0.971*x* + 11, where *x* is the harmonic vibrational wavenumber.

d IR intensities in km mol^–1^ are given in parentheses.

e Percentage integrated IR intensities relative to the most intense band at 1726 cm^–1^ are listed in parentheses.

f Unobserved due to interference from the parent absorption.

g Unobserved due to a poor signal-to-noise ratio caused by the filter cutoff.

The second most intense band, B_6_, observed at 1215 cm^–1^, corresponds closely to the CO-stretching (ν_19_) mode in HPBF-1 (1218 cm^–1^), HPBF-2 (1211 cm^–1^), and HPBF-3 (1212 cm^–1^), and the convoluted band (1214 cm^–1^). Similarly, the B_2_ band observed at 1425 cm^–1^ aligns with the HOO-bending modes predicted for HPBF-1 (1457 cm^–1^, ν_12_), HPBF-2 (1447 cm^–1^, ν_13_), HPBF-3 (1460 cm^–1^, ν_13_), and the convoluted band (1458 cm^–1^). The B_7_ band at 1170 cm^–1^ matches well with the C–CH_3_ stretching mode coupled with the C–C­(O) stretching mode (ν_20_) of HPBF-1 (1162 cm^–1^), HPBF-2 (1144 cm^–1^), HPBF-3 (1158 cm^–1^), and the convoluted band (1162 cm^–1^). Additional weaker bands observed at 1378 (B_3_), 1310 (B_4_), 1247 (B_5_), 1068 (B_8_), 984 (B_9_), and 952 (B_10_) also correspond to predicted vibrational features, as summarized in Table 1. All IR-active bands of HPBF-1 in the 800–2000 cm^–1^ region with predicted IR intensities exceeding 20 km mol^–1^ were experimentally observed. The mean absolute deviations between observed and calculated band positions are 10.7 ± 9.5 cm^–1^ for HPBF-1, 15.1 ± 10.6 cm^–1^ for HPBF-2, 13.7 ± 12.4 cm^–1^ for HPBF-3, and 10.2 ± 10.4 cm^–1^ for the convoluted HPBF spectrum. A detailed comparison of observed band positions with those predicted using anharmonic (fundamental) vibrational treatment is provided in Table S9, with corresponding mean absolute deviations of 11.9 ± 9.4 cm^–1^ for HPBF-1, 16.7 ± 10.2 cm^–1^ for HPBF-2, 13.6 ± 7.4 cm^–1^ for HPBF-3, and 11.7 ± 7.8 cm^–1^ for the convoluted HPBF spectrum. These spectral correlations support the assignment of the observed group A/B features to the hydrogen-transferred intramolecular hydrogen-bonded adduct HPBF, likely present in multiple conformations.

### 4.2 Tentative Assignments of Bands in Group C to Conformers of HPBD and HPBD–FA

Weak and broad features in group C were observed in the continuous-scan detection mode and attributed to enhanced detectivity. Based on the RPS (Figure 2), MVKO may undergo HC­(O)­OH-catalyzed hydrogen transfer to yield HPBD, which can further form a hydrogen-bonded complex with HC­(O)­OH, denoted HPBD–FA.

The spectrum of bands in group C (Figure 5e) is reproduced in Figure 7a. Scaled harmonic vibrational wavenumbers and IR intensities for the two lowest-energy conformers of HPBD, HPBD-1 and HPBD-2, are shown as stick spectra in Figure 7b and c, respectively. Likewise, stick spectra for the four lowest-energy conformers of HPBD–FA (HPBD–FA-1 to HPBD–FA-4) are presented in Figure 7d and g, respectively. The observed features in group C are compared with calculated results for HPBD-1, HPBD-2, and HPBD–FA-1 to HPBD–FA-4 in Table 2. Observed bands C_2_ (1675 cm^–1^), C_3_ (1600 cm^–1^), C_4_ (1432 cm^–1^), C_5_ (1387 cm^–1^), C_6_ (1330 cm^–1^), and C_7_ (1254 cm^–1^) exhibit close correspondence with vibrational modes predicted for HPBD-1 near 1669, 1613, 1433, 1392, 1356, and 1280 cm^–1^, respectively. In contrast, the predicted bands for HPBD-2 near 1653, 1642, 1430, 1394, 1352, and 1206 cm^–1^ show poorer agreement. According to the Boltzmann population distributions, HPBD-1 accounts for ∼93% of the conformational ensemble, making the spectral contribution of HPBD-2 difficult to discern.

**Table 2:** Comparison of Observed Vibrational Wavenumbers and Relative IR Intensities of Features in Group C with the Scaled Harmonic vibrational Wavenumbers and IR Intensities of Conformers C_2_H_3_C­(=CH_2_)­OOH (HPBD-1, HPBD-2) and their Complexes with HC­(O)­OH (HPBD–FA-1, HPBD–FA-2, HPBD–FA-3, and HPBD–FA-4)

		HPBD^a^			HPBD–FA^a^				
label	experiment	mode	HPBD-1	HPBD-2	mode	HPBD–FA-1	HPBD–FA-2	HPBD–FA-3	HPBD–FA-4
C1	1733 (16)^b^				ν_9_	1715^c^ (278.5)^d^	1724^c^ (344.8)^d^	1716^c^ (302.0)^d^	1724^c^ (341.4)^d^
C2	1675 (100)^e^	ν_7_	1669^c^ (22.6)^d^	1653^c^ (61.4)^d^	ν_10_	1673 (27.3)	1661 (4.6)	1663 (74.0)	1658 (2.8)
C3	1600^e^	ν_8_	1613 (78.2)	1642 (27.9)	ν_11_	1616 (62.8)	1595 (85.6)	1652 (7.2)	1623 (70.6)
C4	1432 (21)	ν_9_	1433 (0.1)	1430 (17.6)	ν_12_	1514 (74.0)	1479 (54.7)	1510 (75.0)	1477 (60.2)
					ν_13_	1435 (1.1)	1432 (1.9)	1432 (21.0)	1435 (20.1)
					ν_14_	1401 (2.3)	1388 (4.2)	1404 (0.6)	1391 (2)
C5	1387 (13)	ν_10_	1392 (13.5)	1394 (19.3)	ν_15_	1386 (2.7)	1383 (7.2)	1389 (3.8)	1387 (10.6)
C6	1330 (15)	ν_11_	1356 (46.0)	1352 (46.3)	ν_16_	1355 (12.1)	1331 (11.9)	1356 (15.5)	1335 (11.4)
		ν_12_	1305 (0.5)	1303 (2.5)	ν_17_	1304 (1.4)	1305 (4.5)	1306 (3.4)	1309 (3.9)
C7	1254 (38)^e^	ν_13_	1280 (43.2)	1206 (58.7)	ν_18_	1272 (73.6)	1294 (44.8)	1194 (192.5)	1222 (86.9)
C8	1200^e^				ν_19_	1186 (180)	1170 (185.7)	1185 (85.1)	1171 (163.6)

a Predicted with the B3LYP+D3/aug-cc-pVTZ method.

b Percentage integrated IR intensities relative to the most intense band at 1726 cm^–1^ are listed in parentheses.

c Harmonic vibrational wavenumbers (cm^–1^) scaled with y = 0.971x + 11, in which x is the harmonic vibrational wavenumber.

d IR intensities in km mol^–1^ are given in parentheses.

e Overlapped bands intensities are summed together.

**Figure 7:**
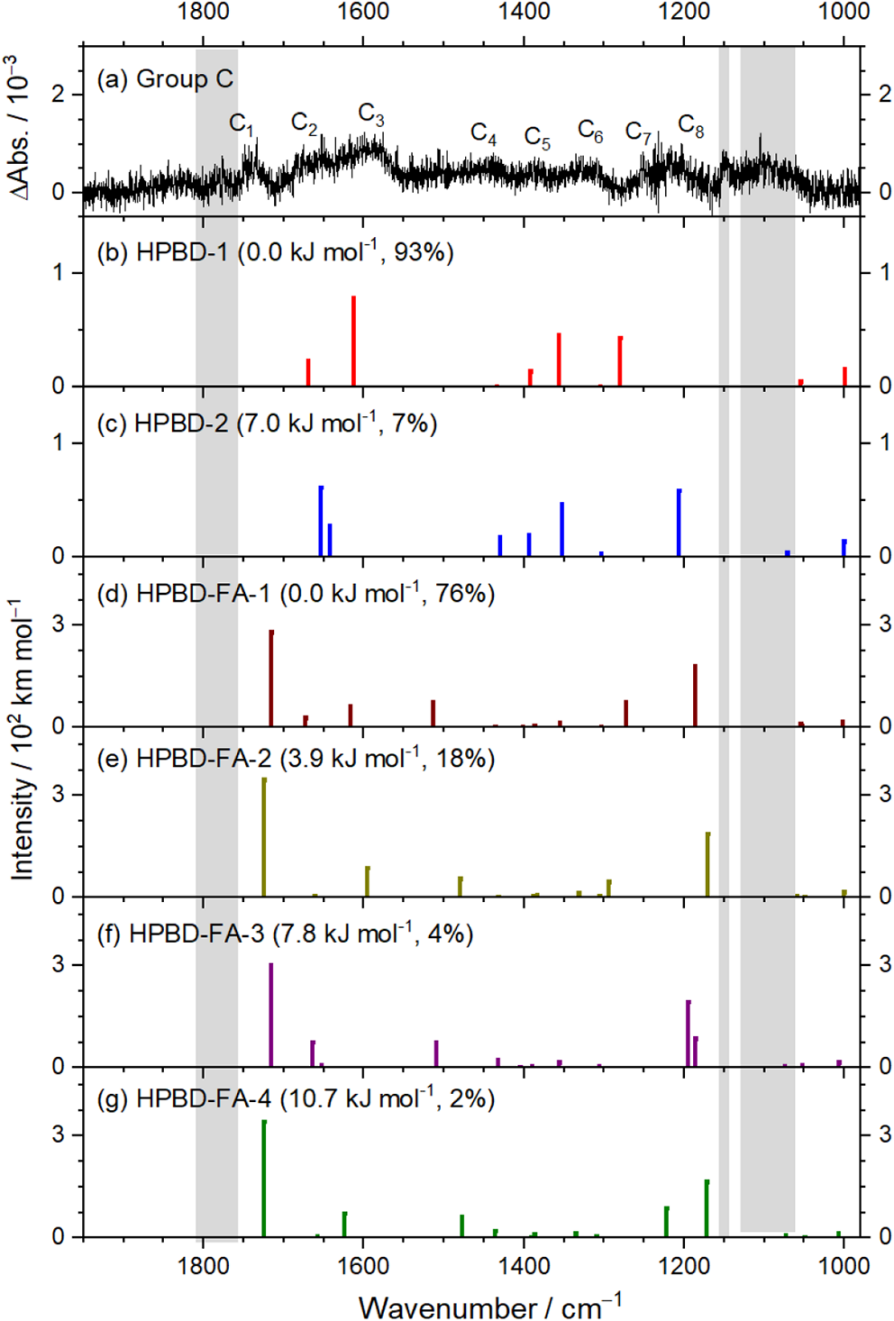
Comparison of bands in group C with stick spectra of two conformers of HPBD, C_2_H_3_C­(=CH_2_)­OOH, and four complexes of HPBD and HC­(O)­OH (HPBD–FA). (a) Absorption spectrum of group C, taken from Figure 5e. IR stick spectra of fundamental bands of HPBD–1 (b), HPBD–2 (c), (d) HPBD–FA-1, (e) HPBD–FA-2, (f) HPBD–FA-3, and (g) HPBD–FA-4, based on scaled harmonic vibrational wavenumbers and IR intensities predicted with the B3LYP + D3/aug-cc-pVTZ method. Regions affected by precursor absorption are shaded gray.

Bands C_1_ (1733 cm^–1^) and C_8_ (1200 cm^–1^) do not correspond to any vibrational modes predicted for HPBD-1 or HPBD-2. However, the two most intense features predicted for HPBD–FA-1 (1715 and 1186 cm^–1^) and HPBD–FA-2 (1724 and 1170) cm^–1^ align well with the observed positions of C_1_ and C_8_. In both HPBD–FA-1 and HPBD–FA-2, the HC­(O)­OH moiety forms a hydrogen bond with HPBD via the interaction between its C=O group and the hydroxyl moiety of HPBD, resulting in a red shift of the C=O stretching bands to ∼1733 cm^–1^. Additionally, three medium-intensity bands predicted for HPBD–FA-1 (1616, 1514, and 1272 cm^–1^) and HPBD–FA-2 (1595, 1479, and 1294 cm^–1^) correspond satisfactorily with the observed bands C_3_ (1600 cm^–1^), C_4_ (1432 cm^–1^), and C_7_ (1254 cm^–1^), respectively. According to Boltzmann population analysis, HPBD–FA-1 and HPBD–FA-2 account for ∼76 and 18% of the conformational ensemble, respectively. The mean absolute deviations between observed and calculated band positions are 12.8 ± 10.9 cm^–1^ (HPBD-1), 23.8 ± 18.3 cm^–1^ (HPBD-1), 22.0 ± 25.6 cm^–1^ (HPBD–FA-1), and 18.8 ± 17.8 cm^–1^ (HPBD–FA-2). The larger deviation for HPBD-2 compared to HPBD-1 supports our assignment of observed features to HPBD-1. Furthermore, a comparison between observed band positions in group C and those of HPBD-1 and HPBD-2 predicted with the anharmonic vibrational treatment is provided in Table S10, with mean absolute deviations of 11.0 ± 6.6 cm^–1^ (HPBD-1) and 30.5 ± 15.9 cm^–1^ (HPBD-2). The larger deviations for the HPBD–FA complexes are due to the interference of their weaker bands from HPBD. Given the broad and weak nature of the features in group C, we tentatively assign them to HPBD, with some contribution from HPBD–FA. The observation of a minute amount of HPBD and HPBD–FA only in experiments at low pressure using the continuous-scan mode is consistent with the expectation that the production of HPBD might be enhanced at low pressure (with less quenching), considering the higher energy of the transition state (TS2 and TS3 in Figure 2) for these pathways.

Vansco et al. employed multiplex photoionization mass spectrometry (MPIMS) to detect both the dissociative photoionization products of adduct DPBF (deuterated HPBF) and acid-catalyzed hydrogen-transfer product DPBD (deuterated HPBD), produced in the reaction of MVKO with DC­(O)­OD. A branching ratio of ∼94:6 for the formation of DPBF versus DPBD was estimated, assuming that *syn*- and *anti*-MVKO are equally populated and that their photoionization cross sections were identical.^40^ We refrain from providing a branching ratio from our data because we could not distinguish the contributions from various conformers of each species, and the quantum-chemically calculated IR intensities are not so reliable. Nevertheless, our observations of mainly HPBF and the weak features attributable to HPBD and HPBD–FA align with the findings of Vansco et al. Notably, HPBD was observed exclusively under continuous-scan detection at low pressure due to its low yield and the enhanced signal-to-noise ratio afforded by the continuous-scan detection. These results further support the proposed formation pathway involving acid-catalyzed hydrogen transfer and validate the presence of both HPBF and HPBD under our experimental conditions.

### 4.3 Absence of Dehydrated End Product

In our previous studies, two conformers of the hydrogen-transfer adduct HPMF were identified in the reaction of CH_2_OO with HC­(O)­OH.^34^ The open-form conformer, HPMF-2, underwent decomposition to form formic anhydride (FAN) and H_2_O at a later period, with a first-order rate coefficient of (1460 ± 30) s^–1^. FAN was detected under continuous-scan data acquisition. Similarly, two conformers of hydrogen-transfer adduct HPMA were observed in the reaction of CH_2_OO with CH_3_C­(O)­OH,^37^ where the open-form conformer decomposed to form formic acetic anhydride (FAA) and H_2_O with a first-order rate coefficient of (980 ± 40) s^–1^. In the reaction of CH_3_CHOO + HC­(O)­OH, the open-form adduct HPEF­(P2) also decomposed to FAA and H_2_O, with a first-order rate coefficient of (1420 ± 70) s^–1^.^35^ In contrast, in this work, the hydrogen-transfer adduct HPBF formed in the reaction of MVKO with HC­(O)­OH was found to be stable and was clearly observed under continuous-scan detection. This stability arises from the absence of a labile hydrogen atom in MVKO; hence, HPBF is unsuitable for dehydration. The vinylic (sp^2^) hydrogen exhibits significantly greater C–H bond strength compared to the C–H (sp^3^) bonds present in CH_2_OO and CH_3_CHOO. Moreover, the three lowest-energy conformers of HPBF are all stabilized by intramolecular hydrogen bonding, which restricts the mobility of the hydroxyl moiety and inhibits dehydration.

## 5 Conclusion

We employed a step-scan FTIR spectrometer to investigate the reaction of MVKO with HC­(O)­OH and identified 10 vibrational bands attributable to the hydrogen-transfer adduct 2-hydroperoxybut-3-en-2-yl formate [HPBF, C_2_H_3_(CH_3_)­C­(OCHO)­OOH]. Vibrational bands in groups A and B were assigned through comparison with quantum-chemical predictions of vibrational wavenumbers and IR intensities of various conformers of HPBF. Although the precise conformation of HPBF cannot be definitively determined, the observed spectra are consistent with the convoluted spectra simulated for the three lowest-energy conformers, weighted by Boltzmann distribution. In contrast to the open-form hydrogen-transfer adducts formed in reactions of HC­(O)­OH with CH_2_OO or CH_3_CHOO—which undergo dehydration over time—HPBF remains stable. This stability arises from the absence of a labile hydrogen atom suitable for OH-induced dehydration, as the vinylic hydrogen in MVKO possesses a C–H bond significantly stronger than the C–H bond in CH_2_OO or CH_3_CHOO. Additionally, the three lowest-energy HPBF conformers exhibit intramolecular hydrogen bonding, restricting hydroxyl mobility and further inhibiting dehydration.

Weak bands in group C were observed under a continuous-scan detection mode. Six of these bands are tentatively assigned to the acid-catalyzed hydrogen-transfer isomer of MVKO, 2-hydroperoxybuta-1,3-diene [HPBD, C_2_H_3_C­(=CH_2_)­OOH], while two additional bands are tentatively attributed to the hydrogen-bonded complex of HPBD with HC­(O)­OH, which also contributes to the six bands attributed to HPBD. The weak signal intensity aligns with the literature report of a 6:94 branching ratio of HPBD:HPBF, as determined by Vansco et al.^40^

## Supplementary Material



## Notes

Cartesian coordinates of geometries of conformers of HPBF, HPBD, HPBD–FA, PRC1, PRC2, and TS1–TS5. Harmonic and anharmonic vibrational wavenumbers and IR intensities of three conformers of HPBF, two conformers of HPBD, and four conformers of HPBD–FA. Comparison of experimentally observed wavenumbers and intensities of (*Z*)-(CH_2_I)­HC=C­(CH_3_)I with quantum-chemical computations. Comparison of observed vibrational wavenumbers and relative IR intensities of features in group A/B with anharmonic vibrational calculations of three conformers of HPBF. Comparison of observed vibrational wavenumbers and relative IR intensities of features in group C with anharmonic vibrational calculations of two conformers of HPBD (Tables S1–S10). Geometries of conformers of HPBF, HPBD, HPBD–FA, PRC1, PRC2, and TS1–TS5. Potential energies for the interconversion of the three lowest-energy conformers of HPBF. Potential energies for the interconversion of two *syn*-conformers of HPBD. Comparison of experimentally observed wavenumbers with the harmonic vibrational wavenumbers of (*Z*)-(CH_2_I)­HC=C­(CH_3_)­I. IR spectra recorded with an external ADC or an internal ADC or in continuous-scan mode following photolysis at 248 nm of a flowing mixture of (*Z*)-(CH_2_I)­HC=C­(CH_3_)­I/HC­(O)­OH/O_2_ at 298 K. Comparison of bands in groups A/B with stick anharmonic spectra of three conformers of HPBF. Comparison of bands in group C with stick anharmonic spectra of two conformers of HPBF (Figures S1–S16) (PDF)
