# Supplementary material for: Reactions of the Criegee Intermediate Methyl Vinyl Ketone Oxide with HC(O)OH: Infrared Spectra of the Hydrogen-Transfer Adduct 2‑Hydroperoxybut-3-en-2-yl Formate
Source: J Phys Chem A. 2025 Dec 3;129(50):11624–36. doi: 10.1021/acs.jpca.5c06912 (PMC12720241; doi:10.1021/acs.jpca.5c06912)
Supplement: Supplementary file 1 [file jp5c06912_si_001.pdf]

Electronic Supplementary Information

Reactions of Criegee Intermediate Methyl Vinyl Ketone Oxide with HC(O)OH: Infrared Spectra of Hydrogen-Transfer Adduct 2-Hydroperoxybut-3-en-2-yl Formate

Yu-Lun Hsiao[†] *and Yuan-Pern Lee*[†,‡*]

[†]Department of Applied Chemistry and Institute of Molecular Science, National Yang Ming Chiao Tung University, 1001, Ta-Hsueh Road, Hsinchu 300093, Taiwan.

[‡]Center for Emergent Functional Matter Science, National Yang Ming Chiao Tung University, Hsinchu 300093, Taiwan

Emails: reverie0323@gmail.com (YLH); yplee@nycu.edu.tw (YPL)

Table of Contents

Table S1. Cartesian coordinates of optimized geometries of eleven conformers of 2-hydroperoxybut-3-en-2-yl formate [HPBF, $C_2H_3C(CH_3)(OCHO)OOH$] -----------------S1
Table S2. Cartesian coordinates of optimized geometries of two conformers of 2-hydroperoxybuta-1,3-diene [HPBD, $(C_2H_3)C(=CH_2)OOH$]--------------------------------S4
Table S3. Cartesian coordinates of optimized geometries of fifteen complexes of HPBD and HC(O)OH (HPBD−FA)---S5
Table S4. Cartesian coordinates of optimized geometries of *syn-trans*-MVKO…FA (PRC1), *syn-cis*-MVKO…FA (PRC2), and transition states TS1−TS5--------------------S9
Table S5. Vibrational wavenumbers and IR intensities of three conformers of HPBF [$C_2H_3C(CH_3)(OCHO)OOH$ -- S11
Table S6. Vibrational wavenumbers and IR intensities of two conformers of HPBD [$C_2H_3C(=CH_2)OOH$] -- S13
Table S7. Vibrational wavenumbers and IR intensities of four complexes of HPBD and HC(O)OH (HPBD−FA)--- S14
Table S8. Comparison of experimentally observed wavenumbers (cm^{-1}) and intensities of (*Z*)-$(CH_2I)HC=C(CH_3)I$ with harmonic and anharmonic vibrational wavenumbers and infrared intensities -- S16
Table S9. Comparison of observed vibrational wavenumbers (in cm^{-1}) and relative IR intensities of features in group A/B with anharmonic vibrational wavenumbers and IR intensities of conformers $(C_2H_3)C(CH_3)(OCHO)OOH$ (HPBF-1, HPBF-2, and HPBF-3)--- S17
Table S10. Comparison of observed vibrational wavenumbers and relative IR intensities of features in group C with the anharmonic vibrational wavenumbers and IR intensities of conformers $C_2H_3C(=CH_2)OOH$ (HPBD-1, HPBD-2)--------------------- S18
Figure S1. Geometries of eleven conformers of $C_2H_3C(CH_3)(OCHO)OOH$ (HPBF) calculated with the B3LYP+D3/aug-cc-pVTZ method------------------------------------ S19
Figure S2. Geometries of three lowest-energy conformers of $C_2H_3C(CH_3)(OCHO)OOH$ (HPBF) calculated with the B3LYP+D3/aug-cc-pVTZ method--- S20
Figure S3. Geometries of two *syn*-conformers of $C_2H_3C(=CH_2)OOH$ (HPBD) calculated with the B3LYP+D3/aug-cc-pVTZ method------------------------------------ S21
Figure S4. Geometries of fifteen conformers of complex of HPBD and HC(O)OH (HPBD−FA) calculated with the B3LYP+D3/aug-cc-pVTZ method -------------------- S22
Figure S5. Geometries of four lowest-energy conformers of complex of HPBD and HC(O)OH (HPBD−FA) calculated with the B3LYP+D3/aug-cc-pVTZ method------- S23
Figure S6. Geometries of two lowest-energy conformers of *syn-trans*-MVKO…FA (PRC1), *syn-cis*-MVKO…FA (PRC2), and transition state TS1−TS5 calculated with the B3LYP+D3/aug-cc-pVTZ method--- S24
Figure S7. Potential energies for the interconversion of three lowest-energy conformers of HPBF calculated with the CCSD(T)/aug-cc-pVTZ//B3LYP+D3/aug-cc-pVTZ method--- S26
Figure S8. Potential energies for the interconversion of two *syn*-conformers of $C_2H_3C(=CH_2)OOH$ (HPBD) calculated with the CCSD(T)/aug-cc-pVTZ//B3LYP+D3/aug-cc-pVTZ method --- S27

Figure S9. Comparison of experimentally observed wavenumbers with the harmonic vibrational wavenumbers of (*Z*)-(CH_2I)HC=C(CH_3)I predicted with the B3LYP/aug-cc-pVTZ-pp method ---------- S28
Figure S10. IR spectra recorded with an external ADC following photolysis at 248 nm of a flowing mixture of (*Z*)-(CH_2I)HC=C(CH_3)I/HC(O)OH/O_2 (0.025/0.038/40.0, P_T = 40.1 Torr) at 298 K ---------- S29
Figure S11. IR spectra recorded with an internal ADC following photolysis at 248 nm of a flowing mixture of (*Z*)-(CH_2I)HC=C(CH_3)I/HC(O)OH/O_2 (0.037/0.055/40.0, P_T = 40.1Torr) at 298 K ---------- S30
Figure S12. IR spectra recorded with an internal ADC following photolysis at 248 nm of a flowing mixture of (*Z*)-(CH_2I)HC=C(CH_3)I/HC(O)OH/O_2 (0.037/0.055/40.0, P_T = 40.1Torr) at 298 K ---------- S31
Figure S13. IR spectra recorded with continuous-scan mode during photolysis at 248 nm of a flowing mixture of (*Z*)-(CH_2I)HC=C(CH_3)I/HC(O)OH/O_2 at 40.8 Torr and 298 K. ---------- S32
Figure S14. IR spectra recorded with continuous-scan mode during photolysis at 248 nm of a flowing mixture of (*Z*)-(CH_2I)HC=C(CH_3)I/HC(O)OH/O_2 at 10.7 Torr and 298 K ---------- S33
Figure S15. Comparison of bands in groups A and B with simulated anharmonic spectra of three lowest-energy conformers of HPBF, (C_2H_3)C(CH_3)(OCHO)OOH ---------- S34
Figure S16. Comparison of bands in group C with stick anharmonic spectra of two conformers of HPBD, $C_2H_3C(=CH_2)OOH$ ---------- S35

Table S1. Cartesian coordinates of optimized geometries of eleven conformers of 2-hydroperoxybut-3-en-2-yl formate [HPBF, $C_2H_3C(CH_3)(OCHO)OOH$]

	HPBF-1 (*syn-cis*)[a]				HPBF-2[a]		
	x	*y*	*z*		*x*	*y*	*z*
C1	2.79855	-0.09591	-0.62400	C1	-2.69037	0.71386	-0.42210
C2	1.78843	-0.74126	-0.06256	C2	-1.91537	-0.32787	-0.16671
C3	0.44288	-0.13042	0.23883	C3	-0.44427	-0.30039	0.16718
C4	0.26427	0.19414	1.71295	C4	-0.15626	-0.87712	1.54435
C5	-1.79049	-1.09049	-0.32280	C5	1.18795	1.56455	0.05060
H1	3.74587	-0.59174	-0.78398	H1	-3.73810	0.57295	-0.64865
H2	2.71119	0.93280	-0.94240	H2	-2.31451	1.72601	-0.41343
H3	1.88256	-1.77427	0.24678	H3	-2.31444	-1.33439	-0.18844
H4	-0.76118	0.46953	1.94074	H4	0.89810	-0.80313	1.79249
H5	0.54387	-0.67666	2.30272	H5	-0.43883	-1.92793	1.55943
H6	0.91617	1.02530	1.97274	H6	-0.74493	-0.33582	2.28186
H7	-1.49714	1.46804	-0.27051	H7	1.99205	-0.82503	-0.49304
H8	-2.25732	-2.06362	-0.51623	H8	1.19857	2.65834	0.12415
O1	0.27303	0.93585	-0.63483	O1	0.15499	-0.98529	-0.89824
O2	-0.64281	1.93513	-0.13514	O2	1.40837	-1.61290	-0.53835
O3	-2.41795	-0.05585	-0.33720	O3	2.19200	0.91473	-0.12227
O4	-0.50051	-1.26584	-0.09338	O4	-0.05885	1.13731	0.17708
	HPBF-3 (*syn-trans*)[a]				HPBF-4[a]		
	x	*y*	*z*		*x*	*y*	*z*
C1	2.95496	-0.33687	-0.06416	C1	0.24473	0.01322	-0.32382
C2	1.76695	-0.31102	-0.64574	C2	0.23470	1.52657	-0.27001
C3	0.46646	0.05816	0.02098	C3	1.32572	-0.69941	0.44725
C4	0.53756	0.46443	1.47630	C4	2.26285	-0.11858	1.17838
C5	-1.62254	-1.32937	-0.02162	C5	-1.58704	-0.33150	1.28849
H1	3.83767	-0.60016	-0.62991	H1	1.15543	1.90159	-0.71295
H2	3.10140	-0.10061	0.98045	H2	0.15167	1.87171	0.75563
H3	1.65904	-0.55109	-1.69633	H3	-0.60416	1.90606	-0.84612
H4	-0.45734	0.61171	1.88330	H4	1.28802	-1.77755	0.34858
H5	1.08436	1.40052	1.56700	H5	2.32451	0.95317	1.30253
H6	1.04255	-0.31214	2.04554	H6	3.00363	-0.71194	1.69602
H7	-1.91526	-2.38538	-0.06051	H7	-1.26052	-0.56604	-2.47481
H8	-1.82545	1.21223	-0.15461	H8	-2.55036	-0.85603	1.34583
O1	-0.07934	1.01346	-0.83957	O1	0.42866	-0.48680	-1.63358
O2	-1.07615	1.84721	-0.20971	O2	-0.54820	0.08704	-2.53703
O3	2.95496	-0.33687	-0.06416	O3	-1.11160	0.30754	2.18403
O4	1.76695	-0.31102	-0.64574	O4	-1.06810	-0.54063	0.06952

	HPBF-5[a]				HPBF-6[a]		
	x	*y*	*z*		*x*	*y*	*z*
C1	0.02659	0.16637	-0.28695	C1	0.27206	-0.10746	0.41501
C2	-0.43767	1.59772	-0.07283	C2	-0.23468	-0.75804	1.70104
C3	1.52440	0.01745	-0.19986	C3	1.73962	-0.38117	0.20572
C4	2.32360	-0.28683	-1.21120	C4	2.28762	-1.04859	-0.79688
C5	-0.46122	-0.59370	2.01065	C5	-1.75270	-0.50721	-0.89617
H1	-0.09466	2.19529	-0.91509	H1	0.43646	-0.50856	2.52070
H2	-0.01614	1.98953	0.84766	H2	-0.23937	-1.83865	1.57077
H3	-1.52224	1.63784	-0.02480	H3	-1.23749	-0.41416	1.93272
H4	1.91628	0.21743	0.78751	H4	2.35735	0.01219	1.00588
H5	1.94960	-0.47960	-2.20614	H5	1.69440	-1.45313	-1.60365
H6	3.39273	-0.34809	-1.06109	H6	3.35679	-1.20616	-0.83522
H7	-2.13759	-0.96160	-1.35626	H7	1.26944	2.47297	-0.16012
H8	-1.06008	-1.35949	2.52206	H8	-2.00702	-0.86330	-1.90325
O1	-0.35830	-0.36384	-1.52655	O1	0.02318	1.27450	0.60643
O2	-1.77107	-0.15326	-1.74448	O2	0.50444	2.02020	-0.54119
O3	0.22189	0.21698	2.57505	O3	-2.55195	-0.13219	-0.08977
O4	-0.62777	-0.73705	0.69190	O4	-0.41342	-0.58581	-0.76298

	HPBF-7[a]				HPBF-8[a]		
	x	*y*	*z*		*x*	*y*	*z*
C1	-0.26456	-0.06307	0.33878	C1	-0.27069	-0.40606	0.28240
C2	0.14054	0.39052	1.73206	C2	0.27068	-1.53684	1.14657
C3	-0.38688	1.01064	-0.71579	C3	-0.41076	0.89793	1.02774
C4	-0.61589	2.29216	-0.47532	C4	-0.29745	2.11020	0.50827
C5	1.92149	-0.83353	-0.42366	C5	1.79822	0.00884	-0.93862
H1	-0.60214	1.07594	2.13348	H1	-0.41967	-1.72188	1.96761
H2	1.10729	0.88295	1.69112	H2	1.24336	-1.26570	1.54490
H3	0.19856	-0.47858	2.38415	H3	0.35843	-2.44195	0.54889
H4	-0.30534	0.65316	-1.73362	H4	-0.66536	0.77556	2.07420
H5	-0.69750	2.69561	0.52455	H5	-0.04369	2.26812	-0.53077
H6	-0.72000	2.99548	-1.28993	H6	-0.44477	2.99088	1.11814
H7	-2.79362	-0.54314	-0.78869	H7	-2.74093	0.54084	-0.38160
H8	2.40261	-1.75415	-0.77850	H8	2.14012	0.06472	-1.98010
O1	-1.48533	-0.74040	0.55521	O1	-1.53897	-0.90049	-0.12778
O2	-2.05556	-1.16417	-0.70919	O2	-2.19771	0.03766	-1.00596
O3	2.47780	0.22501	-0.31732	O3	2.50609	0.16547	0.01660
O4	0.64333	-1.10889	-0.13858	O4	0.48464	-0.25952	-0.94781

	HPBF-9[a]				HPBF-10[a]		
	x	*y*	*z*		*x*	*y*	*z*
C1	0.29549	-0.18009	0.09912	C1	-0.17101	-0.22887	0.06388
C2	0.15545	-1.35081	1.05987	C2	-0.04258	-1.22886	1.19928
C3	1.60864	-0.09735	-0.64457	C3	-0.21929	1.20644	0.52430
C4	2.71674	-0.75394	-0.33639	C4	-1.02062	2.12461	0.00630
C5	-2.00920	-0.28839	-0.76173	C5	2.16791	-0.16084	-0.61598
H1	0.97845	-1.34527	1.77048	H1	-0.90768	-1.15240	1.85341
H2	0.17530	-2.28250	0.49818	H2	0.85342	-1.01940	1.77765
H3	-0.78132	-1.27581	1.60085	H3	0.01827	-2.23824	0.79352
H4	1.58950	0.57365	-1.49232	H4	0.46385	1.45163	1.32577
H5	2.78197	-1.44368	0.49353	H5	-1.71060	1.89440	-0.79288
H6	3.61524	-0.62001	-0.92310	H6	-1.01079	3.13909	0.38048
H7	1.04268	2.53200	0.57433	H7	-2.74460	-1.48992	-0.14328
H8	-2.51138	-0.24162	-1.73734	H8	2.77757	-0.37310	-1.50294
O1	0.05937	0.95847	0.90666	O1	-1.26230	-0.55288	-0.81627
O2	0.26319	2.16916	0.13115	O2	-2.48477	-0.56393	-0.04173
O3	-2.57818	-0.38180	0.28645	O3	2.60271	0.23982	0.42767
O4	-0.68103	-0.22999	-0.98456	O4	0.89113	-0.43388	-0.91447

	HPBF-11[a]		
	x	*y*	*z*
C1	-0.25012	-0.20211	0.02196
C2	-0.20963	-0.17620	1.53588
C3	-0.30043	1.11779	-0.69514
C4	-0.31307	2.31341	-0.12938
C5	2.11023	-0.69066	-0.33704
H1	-1.12422	0.27189	1.91694
H2	0.64351	0.39306	1.89187
H3	-0.13426	-1.19851	1.90376
H4	-0.35421	1.01370	-1.77277
H5	-0.26253	2.45076	0.94173
H6	-0.36883	3.20989	-0.73113
H7	-2.95313	-1.07741	0.49889
H8	2.72333	-1.42938	-0.86845
O1	-1.33492	-1.03828	-0.45425
O2	-2.58000	-0.40151	-0.08317
O3	2.54580	0.22834	0.29513
O4	0.82017	-1.01448	-0.52752

[a] Optimized geometries were predicted with the B3LYP+D3/aug-cc-pVTZ method.

Table S2. Cartesian coordinates of optimized geometries of two conformers of 2-hydroperoxybuta-1,3-diene [HPBD, $(C_2H_3)C(=CH_2)OOH$]

	HPBD-1 (*syn-cis*)[a]				HPBD-2 (*syn-trans*)[a]		
	x	*y*	*z*		*x*	*y*	*z*
C1	-0.70760	1.59667	-0.00115	C1	-2.47504	-0.12774	-0.22428
C2	-0.01117	0.45794	0.00085	C2	-1.30016	-0.56940	0.21420
C3	1.44920	0.40153	0.02053	C3	-0.02652	0.15295	0.08697
C4	2.19576	-0.70140	-0.00534	C4	0.15119	1.47190	0.10985
H1	-0.17013	2.53142	0.01608	H1	-1.23275	-1.53694	0.69848
H2	-1.78293	1.61568	-0.03308	H2	-2.57361	0.81196	-0.75096
H3	1.92186	1.37501	0.05762	H3	-0.70312	2.11231	0.25719
H4	1.76073	-1.68936	-0.04726	H4	2.58303	-0.39089	-0.91150
H5	3.27421	-0.63485	0.01097	H5	1.12647	1.91794	0.01792
H6	-2.21659	-1.12695	0.78138	H6	-3.37702	-0.70155	-0.06503
O1	-0.55194	-0.80811	-0.02111	O1	0.97665	-0.79139	-0.03017
O2	-1.99109	-0.76682	-0.08828	O2	2.28337	-0.18100	-0.01565

[a] Optimized geometries were predicted with the B3LYP+D3/aug-cc-pVTZ method.

Table S3. Cartesian coordinates of optimized geometries of fifteen complexes of HPBD and HC(O)OH (HPBD−FA)

	HPBD−FA-1 [a]				HPBD−FA-2 [a]		
	x	*y*	*z*		*x*	*y*	*z*
C1	-2.80740	-0.63936	-1.18689	C1	-2.80740	-0.63936	-1.18689
C2	-2.18458	-0.81605	-0.02355	C2	-2.18458	-0.81605	-0.02355
C3	-1.14652	0.05696	0.52908	C3	-1.14652	0.05696	0.52908
C4	-0.56348	-0.15975	1.72010	C4	-0.56348	-0.15975	1.72010
C5	2.61732	-0.62136	-0.49329	C5	2.61732	-0.62136	-0.49329
H1	-3.56995	-1.33236	-1.51269	H1	-3.56995	-1.33236	-1.51269
H2	-2.58121	0.19337	-1.83687	H2	-2.58121	0.19337	-1.83687
H3	-2.43727	-1.65823	0.60810	H3	-2.43727	-1.65823	0.60810
H4	0.14412	0.53350	2.14269	H4	0.14412	0.53350	2.14269
H5	-0.89621	-0.99596	2.31505	H5	-0.89621	-0.99596	2.31505
H6	0.95555	1.58396	-0.14446	H6	0.95555	1.58396	-0.14446
H7	3.51498	-1.10592	-0.89195	H7	3.51498	-1.10592	-0.89195
H8	1.09446	-1.05169	0.58395	H8	1.09446	-1.05169	0.58395
O1	-0.85146	1.08142	-0.31830	O1	-0.85146	1.08142	-0.31830
O2	0.13487	1.97388	0.22960	O2	0.13487	1.97388	0.22960
O3	2.35159	0.54034	-0.69325	O3	2.35159	0.54034	-0.69325
O4	1.90044	-1.48181	0.21489	O4	1.90044	-1.48181	0.21489
	HPBD−FA-3 [a]				HPBD−FA-4 [a]		
	x	*y*	*z*		*x*	*y*	*z*
C1	-2.80740	-0.63936	-1.18689	C1	2.65644	-1.38632	-0.37932
C2	-2.18458	-0.81605	-0.02355	C2	2.20897	-0.14695	-0.55202
C3	-1.14652	0.05696	0.52908	C3	1.12042	0.47142	0.22083
C4	-0.56348	-0.15975	1.72010	C4	0.78275	0.18832	1.48710
C5	2.61732	-0.62136	-0.49329	C5	-2.37456	-1.02840	-0.27652
H1	-3.56995	-1.33236	-1.51269	H1	3.47421	-1.76843	-0.97352
H2	-2.58121	0.19337	-1.83687	H2	2.22314	-2.05767	0.35026
H3	-2.43727	-1.65823	0.60810	H3	2.63401	0.48497	-1.32274
H4	0.14412	0.53350	2.14269	H4	1.38562	-0.50783	2.04729
H5	-0.89621	-0.99596	2.31505	H5	-0.00094	0.72768	1.99216
H6	0.95555	1.58396	-0.14446	H6	-1.31352	1.52748	-0.08762
H7	3.51498	-1.10592	-0.89195	H7	-3.15693	-1.72683	-0.59468
H8	1.09446	-1.05169	0.58395	H8	-0.65581	-1.05664	0.56767
O1	-0.85146	1.08142	-0.31830	O1	0.49592	1.38413	-0.58035
O2	0.13487	1.97388	0.22960	O2	-0.54104	2.10233	0.11061
O3	2.35159	0.54034	-0.69325	O3	-2.46512	0.16886	-0.41903
O4	1.90044	-1.48181	0.21489	O4	-1.35899	-1.68173	0.26636

	HPBD−FA-5[a]				HPBD−FA-6[a]		
	x	*y*	*z*		*x*	*y*	*z*
C1	2.63836	1.84662	0.17305	C1	3.73774	-0.84221	0.59708
C2	2.81019	0.52593	0.20239	C2	2.62012	-0.84811	-0.12300
C3	1.77752	-0.48277	-0.02471	C3	1.65925	0.26135	-0.18582
C4	2.00091	-1.79769	0.00575	C4	1.92983	1.56157	-0.10622
C5	-3.18526	0.37768	0.33315	C5	-3.33501	-0.34495	0.43896
H1	3.46924	2.51338	0.35416	H1	4.41956	-1.68043	0.57113
H2	1.67726	2.29613	-0.03068	H2	4.00395	-0.00619	1.22999
H3	3.78860	0.11053	0.40933	H3	2.35768	-1.71975	-0.71184
H4	2.99918	-2.15005	0.21103	H4	2.95594	1.87659	-0.00931
H5	1.22268	-2.51822	-0.17623	H5	1.15797	2.30957	-0.16472
H6	-0.97014	-0.86124	0.31223	H6	-1.05040	0.75839	0.28861
H7	-4.14071	0.86175	0.56243	H7	-4.30548	-0.76735	0.72118
H8	-1.93533	0.21800	-1.07946	H8	-2.14905	-0.27562	-1.03413
O1	0.55708	0.11110	-0.27165	O1	0.40073	-0.28697	-0.36841
O2	-0.46919	-0.86456	-0.53469	O2	-0.60061	0.72625	-0.58573
O3	-2.58544	-0.31651	1.12340	O3	-2.65557	0.31907	1.18972
O4	-2.79758	0.65885	-0.90464	O4	-3.02727	-0.66099	-0.81269
	HPBD−FA-7[a]				HPBD−FA-8[a]		
	x	*y*	*z*		*x*	*y*	*z*
C1	-1.84562	1.95495	-0.72932	C1	3.74162	-0.51286	0.07510
C2	-1.91633	1.13313	0.31816	C2	2.90181	0.51225	-0.05829
C3	-1.42209	-0.24226	0.36288	C3	1.44192	0.43104	-0.06473
C4	-1.49556	-1.01837	1.44758	C4	0.64154	1.49513	-0.16141
C5	2.00692	0.40264	0.02978	C5	-3.24652	0.14506	-0.09773
H1	-2.23478	2.96125	-0.66775	H1	4.81017	-0.35193	0.06751
H2	-1.41481	1.64523	-1.67080	H2	3.39286	-1.52829	0.19550
H3	-2.36654	1.47168	1.24315	H3	3.28580	1.51765	-0.17785
H4	-1.95785	-0.62607	2.33956	H4	1.08590	2.47513	-0.22998
H5	-1.12148	-2.02739	1.45161	H5	-0.43208	1.42481	-0.17427
H6	0.62174	-1.72840	-0.62567	H6	-0.77137	-0.92155	-0.70197
H7	1.06488	0.94287	-0.09846	H7	-4.27358	0.52700	-0.08020
H8	3.79864	0.71211	0.52126	H8	-1.77328	-0.10472	1.07054
O1	-0.85881	-0.60214	-0.83774	O1	1.02372	-0.88006	0.02147
O2	-0.31903	-1.93576	-0.80810	O2	-0.39305	-1.00086	0.19512
O3	2.15142	-0.78081	-0.15629	O3	-2.71513	-0.29605	-1.08906
O4	2.98221	1.22724	0.41870	O4	-2.69137	0.24425	1.10660

	HPBD−FA-9[a]				HPBD−FA-10[a]		
	x	*y*	*z*		*x*	*y*	*z*
C1	0.22211	2.14842	-0.27011	C1	3.82851	-0.62420	0.24416
C2	1.35705	1.58805	0.14732	C2	2.89883	0.21332	-0.20493
C3	1.69240	0.16826	0.08733	C3	1.45079	-0.00336	-0.08084
C4	2.86730	-0.35150	0.43905	C4	0.81581	-1.17349	-0.11228
C5	-2.84073	-0.17157	0.31294	C5	-3.19060	-0.45901	0.10705
H1	0.07959	3.21580	-0.17619	H1	4.87908	-0.42203	0.09057
H2	-0.58851	1.58279	-0.70800	H2	3.57087	-1.53033	0.77613
H3	2.14003	2.19908	0.57915	H3	3.18621	1.13832	-0.69133
H4	3.64153	0.31113	0.79178	H4	1.38535	-2.07280	-0.28014
H5	3.06990	-1.40694	0.38544	H5	-0.24968	-1.26231	0.01259
H6	0.93218	-2.06265	-1.47784	H6	-0.89745	0.90108	0.82851
H7	-3.79413	-0.28572	0.84506	H7	-4.14803	-0.98861	0.04371
H8	-1.01200	-0.64902	0.52125	H8	-1.82345	0.20536	-1.02685
O1	0.61513	-0.57370	-0.38357	O1	0.85362	1.23511	0.07122
O2	0.93192	-1.97347	-0.51332	O2	-0.57482	1.18411	-0.04881
O3	-2.72038	0.39783	-0.74045	O3	-2.68922	-0.10921	1.14939
O4	-1.85884	-0.74996	1.00486	O4	-2.67995	-0.27105	-1.10582

	HPBD−FA-11[a]				HPBD−FA-12[a]		
	x	*y*	*z*		*x*	*y*	*z*
C1	2.51811	1.84244	-0.16356	C1	0.474456	2.123935	-0.97031
C2	2.21011	0.61788	-0.58067	C2	1.107999	1.578175	0.0664
C3	1.36590	-0.33670	0.15307	C3	1.360386	0.156839	0.274406
C4	1.24402	-0.43590	1.47769	C4	2.059948	-0.35035	1.286141
C5	-2.03559	0.57765	0.09107	C5	-2.57821	-0.08865	0.369823
H1	3.16878	2.48045	-0.74473	H1	0.331486	3.193428	-1.02433
H2	2.13658	2.24278	0.76659	H2	0.077578	1.534554	-1.78419
H3	2.57577	0.26255	-1.53727	H3	1.484953	2.206924	0.862932
H4	1.84039	0.20175	2.10978	H4	2.479619	0.324533	2.014856
H5	0.60570	-1.17501	1.93089	H5	2.203302	-1.40901	1.414374
H6	-0.93827	-1.76717	-0.14389	H6	1.671964	-2.16104	-1.2527
H7	-1.03343	0.90823	0.38077	H7	-3.6698	0.011224	0.303546
H8	-3.74604	1.27839	-0.27080	H8	-1.10739	-0.53219	-0.74449
O1	0.71294	-1.11890	-0.77226	O1	0.762941	-0.60187	-0.72909
O2	-0.04650	-2.17502	-0.15724	O2	1.005379	-2.01168	-0.56579
O3	-2.34792	-0.57184	-0.10166	O3	-1.93941	0.10454	1.368459
O4	-2.87162	1.61272	-0.01346	O4	-2.08131	-0.45201	-0.81718

	HPBD−FA-13[a]				HPBD−FA-14[a]		
	x	*y*	*z*		*x*	*y*	*z*
C1	-1.01139	2.41154	-0.40997	C1	1.94321	2.26157	-0.32658
C2	-0.44700	1.42533	0.28453	C2	1.05866	1.29388	-0.10344
C3	-0.97585	0.06727	0.41869	C3	1.42235	-0.11323	0.10312
C4	-0.43398	-0.86085	1.21976	C4	2.49964	-0.59589	0.71259
C5	2.88611	-0.30656	-0.38504	C5	-2.93735	0.35086	0.17460
H1	-0.54429	3.38496	-0.45599	H1	1.62216	3.28810	-0.43359
H2	-1.94406	2.28300	-0.94036	H2	3.00379	2.06220	-0.40771
H3	0.49113	1.58030	0.80149	H3	-0.00210	1.51540	-0.06829
H4	0.41359	-0.58231	1.82669	H4	3.20662	0.09601	1.14036
H5	-0.85861	-1.84402	1.32593	H5	2.67283	-1.65282	0.82059
H6	-2.58416	-1.77069	-1.09940	H6	0.89770	-2.61360	-1.05445
H7	3.79338	-0.39037	-0.99705	H7	-4.02254	0.26614	0.31560
H8	1.13598	-1.03794	-0.29963	H8	-1.42998	-0.78111	-0.09571
O1	-2.07254	-0.11987	-0.38182	O1	0.40828	-0.90082	-0.44825
O2	-2.64543	-1.43136	-0.19522	O2	0.59776	-2.30595	-0.18627
O3	2.77652	0.42055	0.56655	O3	-2.34083	1.39608	0.15539
O4	1.94015	-1.12474	-0.85570	O4	-2.39865	-0.85974	0.03182

	HPBD−FA-15[a]		
	x	*y*	*z*
C1	-0.29079	2.32505	-0.18978
C2	0.88706	1.71750	-0.30157
C3	1.19586	0.39035	0.25033
C4	0.66008	-0.17298	1.33856
C5	2.15458	-0.18257	-0.55162
H1	2.60070	-1.45045	-0.02587
H2	-0.43722	3.31205	-0.60584
H3	-1.13840	1.86197	0.29762
H4	1.70004	2.18790	-0.84200
H5	-0.04112	0.39550	1.92761
H6	0.97204	-1.14342	1.68553
H7	2.34887	-2.03483	-0.75528
H8	-2.71210	-0.80735	-0.31182
O1	-2.90223	0.18846	0.33511
O2	-1.54070	-1.43101	-0.45819
O3	-3.49331	-1.33652	-0.87280
O4	-0.85033	-0.95350	0.05543

[a] Optimized geometries were predicted with the B3LYP+D3/aug-cc-pVTZ method

Table S4. Cartesian coordinates of optimized geometries of *syn-trans*-MVKO…FA (PRC1), *syn-cis*-MVKO…FA (PRC2), and transition states TS1−TS5

	syn-trans-MVKO…FA[a]				*syn-cis*-MVKO…FA[a]		
	x	*y*	*z*		*x*	*y*	*z*
C1	3.12116	-1.01326	-0.23471	C1	2.61527	-1.36252	-0.70778
C2	2.13004	-0.28802	-0.75779	C2	2.25725	-0.55000	0.28924
C3	1.12945	0.41313	0.01571	C3	1.18331	0.42765	0.21494
C4	1.02984	0.47344	1.48315	C4	0.83104	1.31629	1.33533
C5	-2.44478	-1.09312	-0.01800	C5	-2.44939	-0.89772	0.32107
H1	3.82791	-1.52006	-0.87588	H1	3.42239	-2.06918	-0.57920
H2	3.25737	-1.13358	0.83014	H2	2.11505	-1.34493	-1.66585
H3	2.01887	-0.19909	-1.83088	H3	2.77061	-0.59194	1.23996
H4	0.91982	1.51559	1.78614	H4	0.76853	2.34405	0.97420
H5	0.10748	-0.03328	1.77374	H5	-0.17272	1.04830	1.67316
H6	1.88158	0.01166	1.97019	H6	1.54099	1.22190	2.15161
H7	-1.98898	0.76858	-0.23763	H7	-1.77501	0.51091	-0.81539
H8	-3.30999	-1.76648	-0.08949	H8	-3.35189	-1.50666	0.46950
O1	0.28624	1.03478	-0.71061	O1	0.55859	0.49836	-0.88804
O2	-0.72402	1.73017	-0.05137	O2	-0.48364	1.42939	-0.96409
O3	-2.78489	0.14179	-0.31978	O3	-2.60355	-0.06728	-0.68750
O4	-1.34087	-1.48128	0.29970	O4	-1.46426	-1.01229	1.01904
	TS1[a]				TS2[a]		
	x	*y*	*z*		*x*	*y*	*z*
C1	2.91199	-0.72232	-0.14712	C1	-3.04595	-0.08601	-1.05647
C2	1.86174	-0.14247	-0.72324	C2	-2.25025	-0.73155	-0.20238
C3	0.75348	0.45507	0.00793	C3	-1.09081	-0.13475	0.45003
C4	0.71033	0.59170	1.48417	C4	-0.25506	-0.86274	1.33887
C5	-1.76381	-1.36975	-0.00007	C5	2.54613	-0.40346	-0.65813
H1	3.70793	-1.13829	-0.74808	H1	-3.89842	-0.58298	-1.49706
H2	3.01403	-0.81110	0.92476	H2	-2.87187	0.94438	-1.33257
H3	1.78051	-0.07935	-1.80020	H3	-2.44085	-1.76166	0.06411
H4	-0.30295	0.74965	1.82870	H4	0.19615	-0.25107	2.11437
H5	1.30088	1.47515	1.74863	H5	0.70163	-1.12793	0.65902
H6	1.13962	-0.28171	1.96210	H6	-0.70190	-1.78459	1.69302
H7	-2.34360	-2.30476	0.01177	H7	3.48514	-0.60976	-1.20005
H8	-1.84947	0.72717	-0.13647	H8	1.11734	1.27605	0.13460
O1	-0.00119	1.15035	-0.76790	O1	-0.83184	1.07713	0.07400
O2	-1.15674	1.71714	-0.15621	O2	0.30287	1.67057	0.72593
O3	-2.47424	-0.31693	-0.11417	O3	2.18807	0.80272	-0.62156
O4	-0.52899	-1.45182	0.09812	O4	1.96445	-1.39935	-0.16173

	TS3[a]				TS4[a]		
	x	*y*	*z*		*x*	*y*	*z*
C1	2.91199	-0.72232	-0.14712	C1	2.46007	0.06528	0.02560
C2	1.86174	-0.14247	-0.72324	C2	1.31643	-0.61581	-0.05256
C3	0.75348	0.45507	0.00793	C3	0.01004	0.00150	0.06161
C4	0.71033	0.59170	1.48417	C4	-0.38094	1.35040	-0.04657
C5	-1.76381	-1.36975	-0.00007	O1	-0.97649	-0.86731	0.08035
H1	3.70793	-1.13829	-0.74808	O2	-2.20730	-0.19470	-0.04295
H2	3.01403	-0.81110	0.92476	H1	2.47661	1.13219	0.20134
H3	1.78051	-0.07935	-1.80020	H2	3.41288	-0.43241	-0.08522
H4	-0.30295	0.74965	1.82870	H3	1.31533	-1.68828	-0.20594
H5	1.30088	1.47515	1.74863	H4	-1.57488	0.88686	-0.48703
H6	1.13962	-0.28171	1.96210	H5	-0.94436	1.75067	0.79504
H7	-2.34360	-2.30476	0.01177	H6	0.35116	2.03883	-0.44597
H8	-1.84947	0.72717	-0.13647				
O1	-0.00119	1.15035	-0.76790				
O2	-1.15674	1.71714	-0.15621				
O3	-2.47424	-0.31693	-0.11417				
O4	-0.52899	-1.45182	0.09812				

	TS5[a]		
	x	*y*	*z*
C1	2.29890	-0.48457	0.01340
C2	1.41219	0.51008	-0.05142
C3	-0.02571	0.34080	0.06479
C4	-1.01304	1.34448	0.01125
O1	-0.47656	-0.88904	0.03950
O2	-1.88492	-0.87558	-0.06628
H1	1.98945	-1.51250	0.14219
H2	3.35916	-0.28664	-0.05068
H3	1.73919	1.53588	-0.15967
H4	-1.84205	0.41005	-0.47574
H5	-1.70103	1.38520	0.85482
H6	-0.68691	2.32020	-0.32477

[a] Optimized geometries were predicted with the B3LYP+D3/aug-cc-pVTZ method

Table S5. Vibrational wavenumbers and IR intensities of three conformers of HPBF [$C_2H_3C(CH_3)(OCHO)OOH$]

mode	HPBF-1 (*syn-cis*)[a]				HPBF-2[a]				HPBF-3 (*syn-trans*)[a]			
	harm.[b]	int.[c]	anharm.[d]	int.[c]	harm.[b]	int.[c]	anharm.[d]	int.[c]	harm.[b]	int.[c]	anharm.[d]	int.[c]
ν_1	3383	287.8	3263	225.5	3496	261.6	3298	215.4	3444	313.4	3244	262.3
ν_2	3154	2.6	3099	4.4	3239	3.3	3098	4.2	3230	5.7	3091	6.8
ν_3	3092	2.9	3047	4.2	3170	3.0	3046	2.8	3167	1.1	3056	1.9
ν_4	3070	6.5	3013	7.2	3152	3.0	3018	7.1	3155	4.6	3027	4.2
ν_5	3068	5.1	2994	2.9	3150	7.8	2989	6.0	3149	4.4	2997	3.9
ν_6	3056	5.6	3003	4.9	3136	5.5	2997	5.7	3138	4.8	3006	6.1
ν_7	2984	5.7	2953	6.5	3063	4.9	2957	3.4	3069	4.5	2968	3.8
ν_8	2955	51.9	2881	54.0	3035	46.5	2876	43.2	3039	51.0	2886	50.1
ν_9	1702	310.6	1708	54.9	1750	291.4	1719	142.2	1740	329.4	1703	72.7
ν_{10}	1670	1.9	1671	0.3	1711	21.4	1671	0.6	1716	0.3	1675	0.3
ν_{11}	1461	3.3	1450	2.9	1493	4.2	1453	4.1	1498	4.3	1453	1.4
ν_{12}	1457	100.9	1458	42.3	1487	7.0	1437	39.6	1495	22.1	1460	2.9
ν_{13}	1452	1.1	1444	2.0	1479	78.3	1447	18.3	1492	70.8	1450	25.8
ν_{14}	1420	14.1	1414	10.9	1454	15.1	1428	10.3	1455	15.2	1420	11.6
ν_{15}	1385	15.9	1384	9.8	1414	16.2	1389	11.5	1417	20.0	1387	13.1
ν_{16}	1377	1.7	1375	7.3	1409	2.5	1371	3.2	1410	2.0	1375	7.3
ν_{17}	1305	0.9	1308	2.0	1334	0.7	1310	1.2	1335	3.5	1316	2.4
ν_{18}	1241	88.3	1233	53.4	1263	44.5	1229	10.3	1299	14.6	1263	3.9
ν_{19}	1218	173.2	1207	124.9	1236	283.7	1187	95.2	1236	312.2	1211	64.4
ν_{20}	1162	97.2	1152	37.7	1167	48.1	1134	29.2	1181	31.4	1154	15.3
ν_{21}	1073	138.1	1071	75.6	1127	116.9	1087	55.0	1090	137.7	1061	75.0
ν_{22}	1044	11.1	1044	19.6	1052	18.6	1036	24.2	1047	2.4	1031	0.8
ν_{23}	1028	1.5	1028	0.1	1047	1.7	1016	1.5	1029	9.0	1016	9.8
ν_{24}	1009	16.4	997	15.5	1029	12.5	1026	20.7	1027	15.9	991	15.6
ν_{25}	974	34.7	973	36.8	986	38.3	967	30.5	980	40.1	964	38.3
ν_{26}	959	7.9	946	1.0	958	5.3	933	5.6	968	0.5	942	3.3
ν_{27}	915	10.3	906	2.6	924	11.5	907	8.9	921	21.5	903	14.8
ν_{28}	812	9.3	811	5.9	837	83.7	815	85.2	827	62.0	805	6.1
ν_{29}	784	104.1	776	56.6	817	11.6	805	4.8	804	67.4	790	20.2
ν_{30}	707	41.0	706	51.6	697	44.7	719	20.1	722	30.7	744	9.1
ν_{31}	673	70.5	709	10.5	685	14.3	664	12.0	677	89.8	561	62.6
ν_{32}	636	59.7	526	76.7	638	83.5	577	90.1	613	46.8	611	41.0
ν_{33}	554	3.9	547	6.0	578	12.9	571	14.0	561	7.3	544	15.9
ν_{34}	500	6.7	492	5.6	495	6.2	485	4.3	490	9.8	480	10.4
ν_{35}	463	5.6	453	6.3	444	4.6	436	3.1	432	8.8	432	7.0
ν_{36}	386	11.3	377	9.7	413	4.1	407	3.3	404	4.8	398	3.9

ν_{37}	357	2.1	344	1.9	357	4.8	346	5.4	347	0.8	359	0.4
ν_{38}	297	3.5	288	7.1	310	2.9	301	0.9	299	10.7	297	15.3
ν_{39}	294	4.0	296	5.6	277	8.9	281	3.7	266	3.1	210	2.1
ν_{40}	268	5.9	271	3.8	254	3.1	242	4.1	256	2.6	254	3.6
ν_{41}	258	3.1	240	4.1	251	2.5	200	2.6	246	1.5	283	0.7
ν_{42}	233	2.8	190	0.1	225	2.7	249	12.4	235	4.6	229	5.4
ν_{43}	181	6.0	172	5.3	185	6.7	158	4.4	190	8.6	174	5.5
ν_{44}	116	1.0	106	1.0	83	0.9	94	1.0	74	0.2	77	1.0
ν_{45}	81	0.2	64	0.1	71	0.3	38	0.2	63	0.8	73	0.6

mode[e]	anharm.[d]	int.[c]	mode[e]	anharm.[d]	int.[c]	mode[e]	anharm.[d]	int.[c]
$2\nu_{37}$	3393	41.0	$\nu_{22} + \nu_{30}$	1713	45.1	$2\nu_{9}$	3387	18.8
$\nu_{12} + \nu_{40}$	1708	23.9	$\nu_{13} + \nu_{39}$	1706	71.2	$2\nu_{13}$	2874	15.8
$\nu_{13} + \nu_{40}$	1698	18.8	$\nu_{12} + \nu_{42}$	1661	16.1	$\nu_{13} + \nu_{41}$	1701	19.5
$\nu_{20} + \nu_{33}$	1697	83.1	$\nu_{21} + \nu_{37}$	1428	17.7	$\nu_{20} + \nu_{33}$	1699	23.1
$\nu_{14} + \nu_{39}$	1695	159.7	$\nu_{16} + \nu_{45}$	1428	15.7	$\nu_{18} + \nu_{35}$	1696	71.2
$\nu_{29} + \nu_{31}$	1418	27.3	$\nu_{30} + \nu_{32}$	1263	23.9	$\nu_{19} + \nu_{34}$	1687	30.1
$\nu_{30} + \nu_{33}$	1240	35.1	$\nu_{22} + \nu_{43}$	1212	36.9	$\nu_{11} + \nu_{42}$	1684	159.9
$\nu_{31} + \nu_{32}$	1214	18.7	$\nu_{23} + \nu_{43}$	1197	21.9	$\nu_{27} + \nu_{33}$	1440	30.3
$\nu_{29} + \nu_{36}$	1151	34.5	$\nu_{26} + \nu_{39}$	1195	66.5	$\nu_{30} + \nu_{31}$	1287	15.2
$\nu_{28} + \nu_{37}$	1148	20.5	$2\nu_{32}$	1193	25.2	$\nu_{22} + \nu_{43}$	1216	213.3
$\nu_{32} + \nu_{35}$	1048	27.2	$\nu_{20} + \nu_{45}$	1191	51.0	$\nu_{26} + \nu_{40}$	1196	28.3
$\nu_{34} + \nu_{39}$	771	29.7	$\nu_{32} + \nu_{34}$	1101	19.0	$\nu_{26} + \nu_{39}$	1192	27.0
			$\nu_{31} + \nu_{35}$	1101	20.8	$2\nu_{31}$	1180	24.0
						$\nu_{32} + \nu_{34}$	1081	30.1
						$2\nu_{36}$	795	81.0
						$\nu_{33} + \nu_{41}$	794	15.7

[a] Predicted using the B3LYP+D3/aug-cc-pVTZ method. [b] Harmonic vibrational wavenumbers x (in cm^{-1}) are scaled according to $y = 0.971 x + 11.0$. [c] IR intensity in unit of km mol^{-1}. [d] Anharmonic vibrational wavenumbers in cm^{-1}. [e] Combination and overtone vibrational mode with IR intensities $>$15 km mol^{-1} are listed.

Table S6. Vibrational wavenumbers and IR intensities of two conformers of HPBD [$C_2H_3C(=CH_2)OOH$]

mode	HPBD-1 (*syn-cis*)[a]				HPBD-2 (*syn-trans*)[a]			
	harm.[b]	int.[c]	anharm.[d]	int.[c]	harm.[b]	int.[c]	anharm.[d]	int.[c]
ν_1	3639	46.7	3547	34.3	3664	51.9	3607	64.1
ν_2	3194	0.9	3143	1.3	3207	0.3	3149	0.5
ν_3	3154	4.7	3094	8.1	3159	6.1	3112	7.4
ν_4	3101	1.5	3051	2.9	3113	0.6	3078	0.8
ν_5	3083	2.8	3070	1.7	3087	3.7	3036	5.5
ν_6	3070	2.4	2984	1.9	3073	4.5	2996	3.5
ν_7	1669	22.6	1655	18.0	1653	61.4	1647	25.6
ν_8	1613	78.2	1609	63.3	1642	27.9	1628	43.8
ν_9	1433	0.1	1434	0.3	1430	17.6	1416	33.3
ν_{10}	1392	13.5	1394	29.9	1394	19.3	1436	18.4
ν_{11}	1356	46.0	1326	11.9	1352	46.3	1307	1.5
ν_{12}	1305	0.5	1313	0.2	1303	2.5	1283	11.2
ν_{13}	1280	43.2	1265	19.2	1206	58.7	1207	23.1
ν_{14}	1054	4.5	1057	6.7	1071	3.8	1070	2.9
ν_{15}	999	15.4	972	4.0	1000	13.8	1000	10.9
ν_{16}	966	7.3	953	7.1	958	17.5	956	8.7
ν_{17}	943	39.9	943	49.6	942	31.6	937	41.6
ν_{18}	914	26.5	909	25.0	909	33.3	904	35.5
ν_{19}	848	55.7	845	29.1	852	9.7	847	8.2
ν_{20}	839	7.1	832	21.7	833	52.5	832	33.0
ν_{21}	753	1.0	763	0.1	730	2.5	732	2.3
ν_{22}	713	0.5	720	0.6	714	1.2	690	1.2
ν_{23}	576	4.9	573	5.1	589	8.8	587	13.3
ν_{24}	495	3.2	486	4.0	499	6.8	490	7.1
ν_{25}	452	5.5	441	3.9	407	5.0	401	2.9
ν_{26}	369	1.4	356	0.2	364	0.6	350	3.3
ν_{27}	242	0.2	234	2.2	256	0.5	250	1.1
ν_{28}	184	103.7	92	75.5	145	12.1	122	283.1
ν_{29}	168	3.1	170	8.9	108	83.1	174*i*	2600.3
ν_{30}	107	5.2	65	3.6	96	27.0	9*i*	96.9
	mode[e]		anharm.[d]	int.[c]	mode[e]		anharm.[d]	int.[c]
					$\nu_7 + \nu_9$		1184.4	24.7

[a] Predicted using the B3LYP+D3/aug-cc-pVTZ method. [b] Harmonic vibrational wavenumbers x (in cm^{-1}) are scaled according to $y = 0.971\ x + 11.0$. [c] IR intensity in unit of km mol^{-1}. [d] Anharmonic vibrational wavenumbers in cm^{-1}.

Table S7. Vibrational wavenumbers and IR intensities of four complexes of HPBD and HC(O)OH (HPBD−FA)

mode	HPBD−FA-1[a]		HPBD−FA-2[a]		HPBD−FA-3[a]		HPBD−FA-4[a]	
	harm.[b]	int.[c]	harm.[b]	int.[c]	harm.[b]	int.[c]	harm.[b]	int.[c]
ν_1	3331	1408.8	3380	847.4	3332	1401.7	3347	955.9
ν_2	3263	22.4	3274	631.8	3261	66.2	3213	492.9
ν_3	3199	1.1	3192	1.2	3198	0.3	3171	3.8
ν_4	3155	6.1	3159	3.1	3149	5.3	3162	4.4
ν_5	3104	2.0	3097	3.2	3106	1.2	3085	3.4
ν_6	3087	2.0	3088	2.6	3071	3.0	3081	4.2
ν_7	3073	1.6	3073	2.1	3063	4.6	3072	2.9
ν_8	2983	71.3	2972	82.4	2983	72.9	2977	84.8
ν_9	1714	278.5	1723	344.8	1714	302.0	1714	304.0
ν_{10}	1671	27.3	1660	4.6	1662	74.0	1669	10.6
ν_{11}	1615	62.8	1594	85.6	1651	7.2	1614	40.2
ν_{12}	1512	74.0	1477	54.7	1508	75.0	1479	57.8
ν_{13}	1434	1.1	1430	1.9	1430	21.0	1436	4.4
ν_{14}	1399	2.3	1386	4.2	1402	0.6	1409	0.3
ν_{15}	1384	2.7	1381	7.2	1387	3.8	1387	4.7
ν_{16}	1353	12.1	1329	11.9	1354	15.5	1356	15.4
ν_{17}	1301	1.4	1303	4.5	1304	3.4	1302	0.7
ν_{18}	1270	73.6	1292	44.8	1192	192.5	1266	55.7
ν_{19}	1184	180.0	1168	185.7	1183	85.1	1189	193.8
ν_{20}	1051	9.2	1056	4.3	1071	2.6	1052	8.6
ν_{21}	1049	5.2	1046	1.6	1049	7.3	1044	14.1
ν_{22}	999	15.9	997	15.7	1004	15.8	998	13.1
ν_{23}	964	8.3	976	7.2	959	22.2	955	34.3
ν_{24}	940	41.7	957	36.6	950	23.2	947	44.4
ν_{25}	907	35.7	920	18.6	901	46.0	917	63.9
ν_{26}	876	111.2	861	96.6	880	133.3	901	52.4
ν_{27}	861	47.2	837	12.3	859	50.6	862	10.3
ν_{28}	832	10.5	780	33.2	844	12.8	822	3.0
ν_{29}	750	1.4	757	5.5	731	4.2	750	5.7
ν_{30}	714	0.7	737	3.1	719	0.2	715	4.2
ν_{31}	683	22.5	669	60.4	686	25.7	686	22.5
ν_{32}	663	66.9	641	53.8	661	68.1	654	70.5
ν_{33}	570	3.4	576	4.6	590	4.6	608	14.8
ν_{34}	495	2.5	491	1.7	492	6.4	477	6.0
ν_{35}	448	3.0	466	3.8	404	0.9	439	7.7
ν_{36}	365	2.8	370	1.2	352	3.4	345	10.5

ν_{37}	276	20.6	248	21.3	279	24.3	261	6.8
ν_{38}	231	15.7	241	0.7	233	17.9	228	8.4
ν_{39}	208	1.5	189	11.7	212	2.3	222	12.9
ν_{40}	193	1.3	169	0.4	197	1.1	188	14.3
ν_{41}	175	12.7	160	4.8	177	12.9	158	0.2
ν_{42}	149	0.6	120	2.2	111	0.1	154	1.0
ν_{43}	66	0.5	93	0.1	56	0.4	77	1.0
ν_{44}	46	0.4	54	0.2	48	0.5	49	0.2
ν_{45}	39	0.4	44	0.1	38	0.3	44	1.1

[a] Predicted using the B3LYP+D3/aug-cc-pVTZ method. [b]Harmonic vibrational wavenumbers x (in cm^{-1}) are scaled according to $y = 0.971\ x + 11.0$. [c] IR intensity in unit of km mol^{-1}. [d] Anharmonic vibrational wavenumbers in cm^{-1}.

Table S8. Comparison of experimentally observed wavenumbers (cm^{-1}) and intensities of (*Z*)-$(CH_2I)HC{=}C(CH_3)I$ with harmonic and anharmonic vibrational wavenumbers and infrared intensities

mode	experiment		harmonic[a]		scale harmonic[a, b]		anharmonic[a]	
	ν / cm^{-1}	int.[c]	ν / cm^{-1}	int.[d]	ν / cm^{-1}	dev.[e]	ν / cm^{-1}	dev.[e]
ν_7	1641	(17)	1684	33.5	1646	5	1640	−1
ν_{10}	1434	(22)	1467	12.5	1435	1	1425	−9
ν_{12}	1295	(29)	1329	29.3	1302	7	1304	9
ν_{13}	1169	(100)	1182	30.8	1159	−10	1154	−15
ν_{14}	1152		1168	73.3	1145	−7	1139	−13
ν_{16}	1062	(25)	1070	31.9	1053	−11	1047	−15
ν_{19}	838	(17)	869	15.3	855	17	854	16
					avg. dev.[f]	8.3 ± 5.1	avg. dev.[f]	11.1 ± 5.3

[a] Computed with the B3LYP/aug-cc-pVTZ-pp method. [b] Harmonic vibrational wavenumbers x are scaled according to $y = 0.971\ x + 11.0$. [c] Integrated IR intensities relative to that of the overlapped bands of 1153 and 1169 cm^{-1}. [d] Harmonic IR intensity in unit of km mol^{-1} [e] Deviation = predicted vibrational wavenumber – experimental value. [f] Averaged absolute deviations; the listed error reflects one standard deviation in fitting.

Table S9. Comparison of observed vibrational wavenumbers (in cm^{-1}) and relative IR intensities of features in group A/B with anharmonic vibrational wavenumbers and IR intensities of conformers $(C_2H_3)C(CH_3)(OCHO)OOH$ (HPBF-1, HPBF-2, and HPBF-3)

mode	HPBF conformers[a]			convoluted[b]	experiment
	HPBF-1	HPBF-2	HPBF-3		
ν_9	1708[c] (54.9)[d]	1719[c] (142.2)[d]	1703[c] (72.7)[d]	1712 (85.7)	1726 (1)[e]
ν_{10}	1671 (0.3)	1671 (0.6)	1675 (0.3)	1671 (0.4)	
ν_{11}	1450 (2.9)	1453 (4.1)	1453 (1.4)	1453 (50.2)	1425 (0.54)
ν_{12}	1458 (42.3)	1437 (39.6)	1460 (2.9)		
ν_{13}	1444 (2.0)	1447 (18.3)	1450 (25.8)		
ν_{14}	1414 (10.9)	1428 (10.3)	1420 (11.6)	1419 (10.8)	
ν_{15}	1384 (9.8)	1389 (11.5)	1387 (13.1)	1382 (16.7)	1378 (0.28)
ν_{16}	1375 (7.3)	1371 (3.2)	1375 (7.3)		
ν_{17}	1308 (2.0)	1310 (1.2)	1316 (2.4)	1308 (1.8)	1310 (0.04)
ν_{18}	1233 (53.4)	1229 (10.3)	1263 (3.9)	1233 (33.7)	1247 (0.41)
ν_{19}	1207 (124.9)	1187 (95.2)	1211 (64.4)	1207 (108.4)	1215 (0.60)
ν_{20}	1152 (37.7)	1134 (29.2)	1154 (15.3)	1151 (32.4)	1170 (0.49)
ν_{21}	1071 (75.6)	1087 (55.0)	1061 (75.0)	1074 (68.7)	1068 (0.54)
ν_{22}	1044 (19.6)	1036 (24.2)	1031 (0.8)	1037 (20.6)	
ν_{23}	1028 (0.1)	1016 (1.5)	1016 (9.8)		
ν_{24}	997 (15.5)	1026 (20.7)	991 (15.6)	997 (17.2)	
ν_{25}	973 (36.8)	967 (30.5)	964 (38.3)	970 (34.9)	984 (0.08)
ν_{26}	946 (1.0)	933 (5.6)	942 (3.3)	944 (2.8)	952 (0.30)
ν_{27}	906 (2.6)	907 (8.9)	903 (14.8)	905 (6.0)	
ν_{28}	811 (5.9)	815 (85.2)	805 (6.1)	813 (32.1)	[f]

[a] Predicted with the B3LYP+D3/aug-cc-pVTZ method. [b] Constructed from the simulated spectra of HPBF-1, HPBF-2, and HPBF-3 using their predicted Boltzmann population distribution of 56%, 33%, and 11%, respectively. [c] Harmonic vibrational wavenumbers (cm^{-1}) scaled with $y = 0.971\ x + 11$, in which x is the harmonic vibrational wavenumber. [d] IR intensities in km mol^{-1} are given in parentheses. [e] Percentage integrated IR intensities relative to the most intense band at 1726 cm^{-1} are listed in parentheses. [f] Unobserved due to poor signal-to-noise ratio caused by the filter cutoff.

Table S10. Comparison of observed vibrational wavenumbers and relative IR intensities of features in group C with the anharmonic vibrational wavenumbers and IR intensities of conformers $C_2H_3C(=CH_2)OOH$ (HPBD-1, HPBD-2)

mode	experiment		HPBD[a]		Mode description[a]
			HPBD-1	HPBD-2	
υ_7	1675	(100)[b,c]	1655 (18)[d]	1647 (25.6)[d]	iph C=C str.
υ_8	1600	[c]	1609 (63.3)	1628 (43.8)	oph C=C str.
υ_9	1432	(21)	1434 (0.3)	1436 (33.3)	iph CH_2 scissor
υ_{10}	1387	(13)	1394 (29.9)	1416 (18.4)	oph CH_2 scissor
υ_{11}			1326 (11.9)	1307 (1.5)	HOO bend
υ_{12}	1330	(15)	1313 (0.2)	1283 (11.2)	ip CH bend
υ_{13}	1254	(38)[e]	1265 (19.2)	1207 (23.1)	iph CCC bend
υ_{14}			1057 (6.7)	1070 (2.9)	CH_2 rock
υ_{15}			972 (4.0)	1000 (10.9)	oop CH bend
υ_{16}			953 (7.1)	956 (8.7)	OO str.
υ_{17}			943 (49.6)	937 (41.6)	CH_2 wag
υ_{18}			909 (25.0)	904 (35.5)	CH_2 rock
υ_{19}			845 (29.1)	847 (8.2)	CH_2 wag
υ_{20}			832 (21.7)	832 (33.0)	COO bend

[a] Predicted with the B3LYP+D3/aug-cc-pVTZ method. [b] Percentage integrated IR intensities relative to the most intense band at 1726 cm^{-1} are listed in parentheses. [c] Overlapped bands; intensities are summed together. [d] IR intensities in km mol^{-1} are given in parentheses. [e] Overlapped with a band at 1200 cm^{-1}; intensities are summed together.

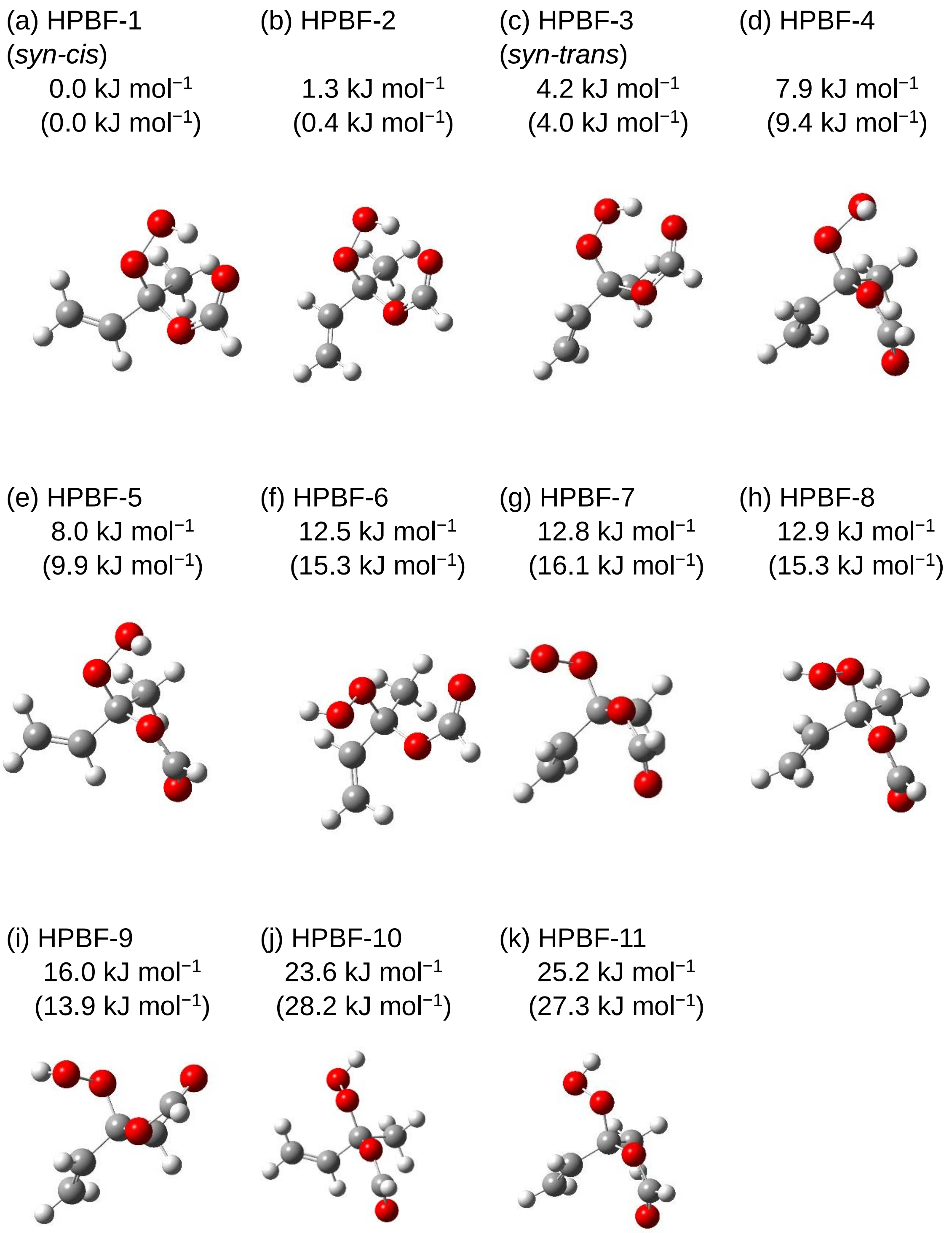

Figure S1. Geometries of eleven conformers of $C_2H_3C(CH_3)(OCHO)OOH$ (HPBF) calculated with the B3LYP+D3/aug-cc-pVTZ method. Relative energies were calculated with the CCSD(T)/aug-cc-pVTZ//B3LYP+D3/aug-cc-pVTZ method; those calculated with the B3LYP+D3/aug-cc-pVTZ method are listed in parentheses for comparison.

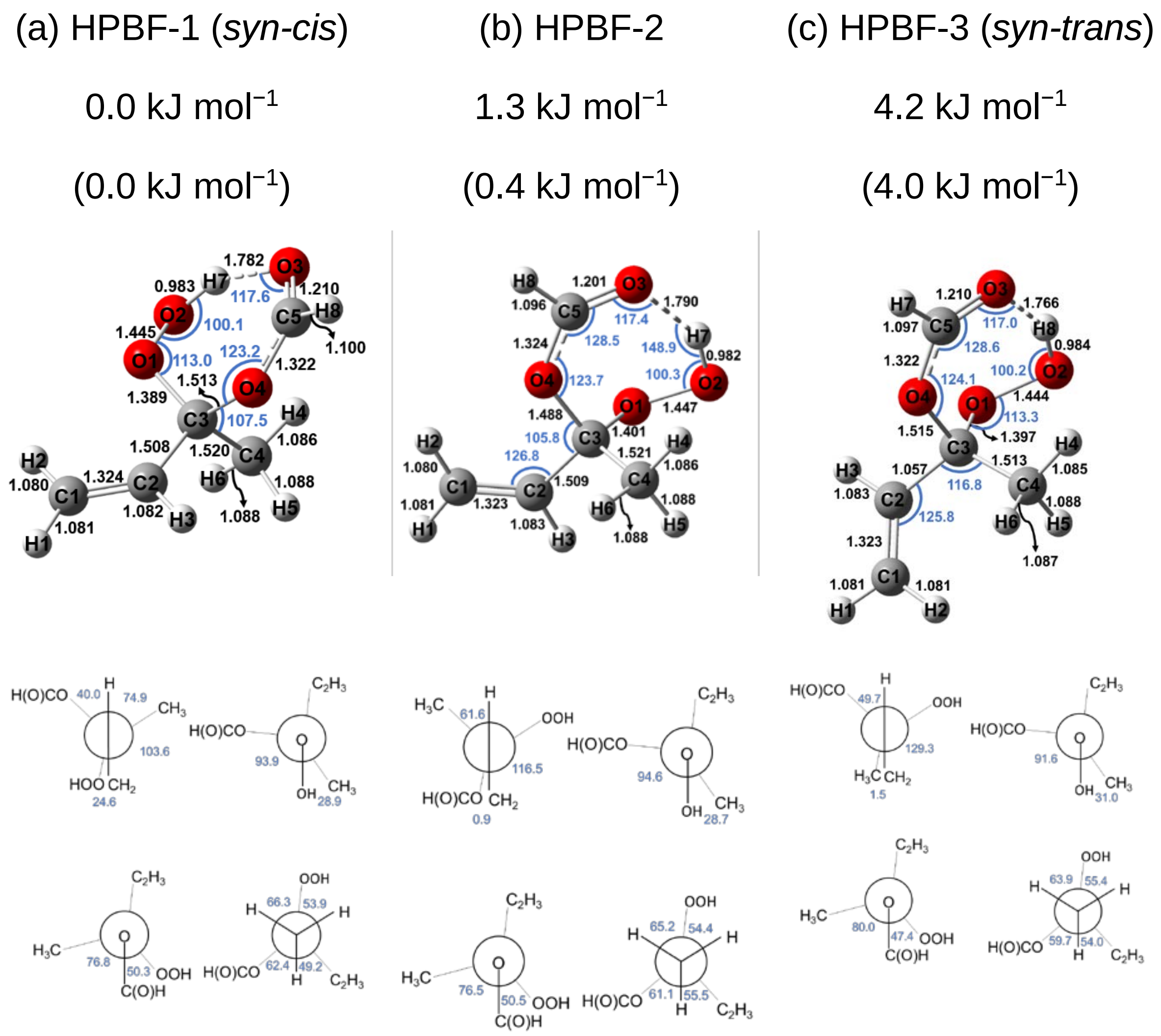

Figure S2. Geometries of three lowest-energy conformers of $C_2H_3C(CH_3)(OCHO)OOH$ (HPBF) calculated with the B3LYP+D3/aug-cc-pVTZ method. (a) HPBF-1, (b) HPBF-2, and (c) HPBF-3. Bond lengths are in Å and bond angles in degrees. Relative energies were calculated with the CCSD(T)/aug-cc-pVTZ//B3LYP+D3/aug-cc-pVTZ method; those calculated with the B3LYP+D3/aug-cc-pVTZ method are listed in parentheses for comparison.

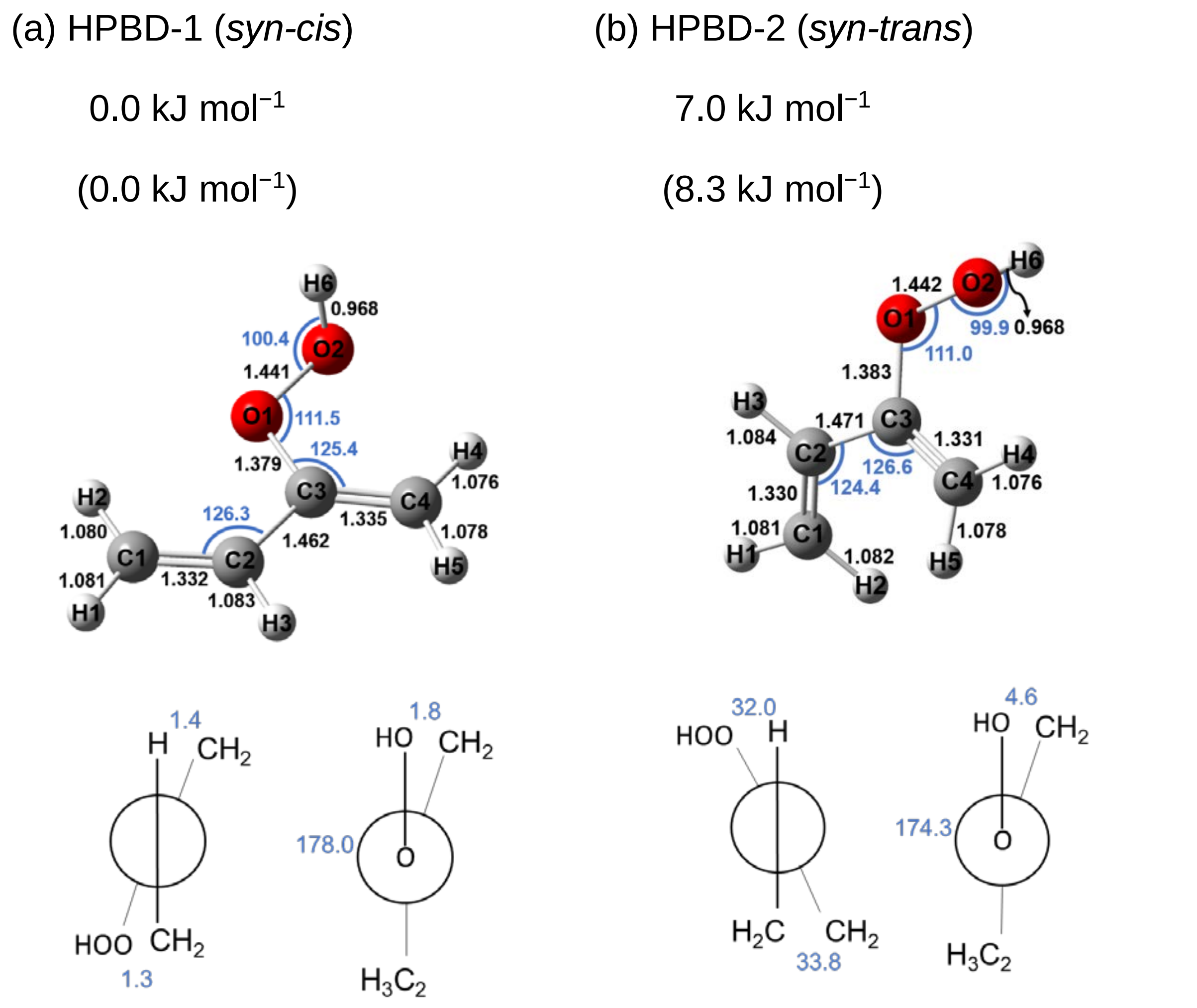

Figure S3. Geometries of two *syn*-conformers of $C_2H_3C(=CH_2)OOH$ (HPBD) calculated with the B3LYP+D3/aug-cc-pVTZ method. (a) HPBD-1 and (b) HPBD-2. Bond lengths are in Å and bond angles in degrees. Relative energies were calculated with the CCSD(T)/aug-cc-pVTZ//B3LYP+D3/aug-cc-pVTZ method; those calculated with the B3LYP+D3/aug-cc-pVTZ method are listed in parentheses for comparison. HPBD-1 is 67.8 kJ mol^{-1} higher in energy than HPBF-1.

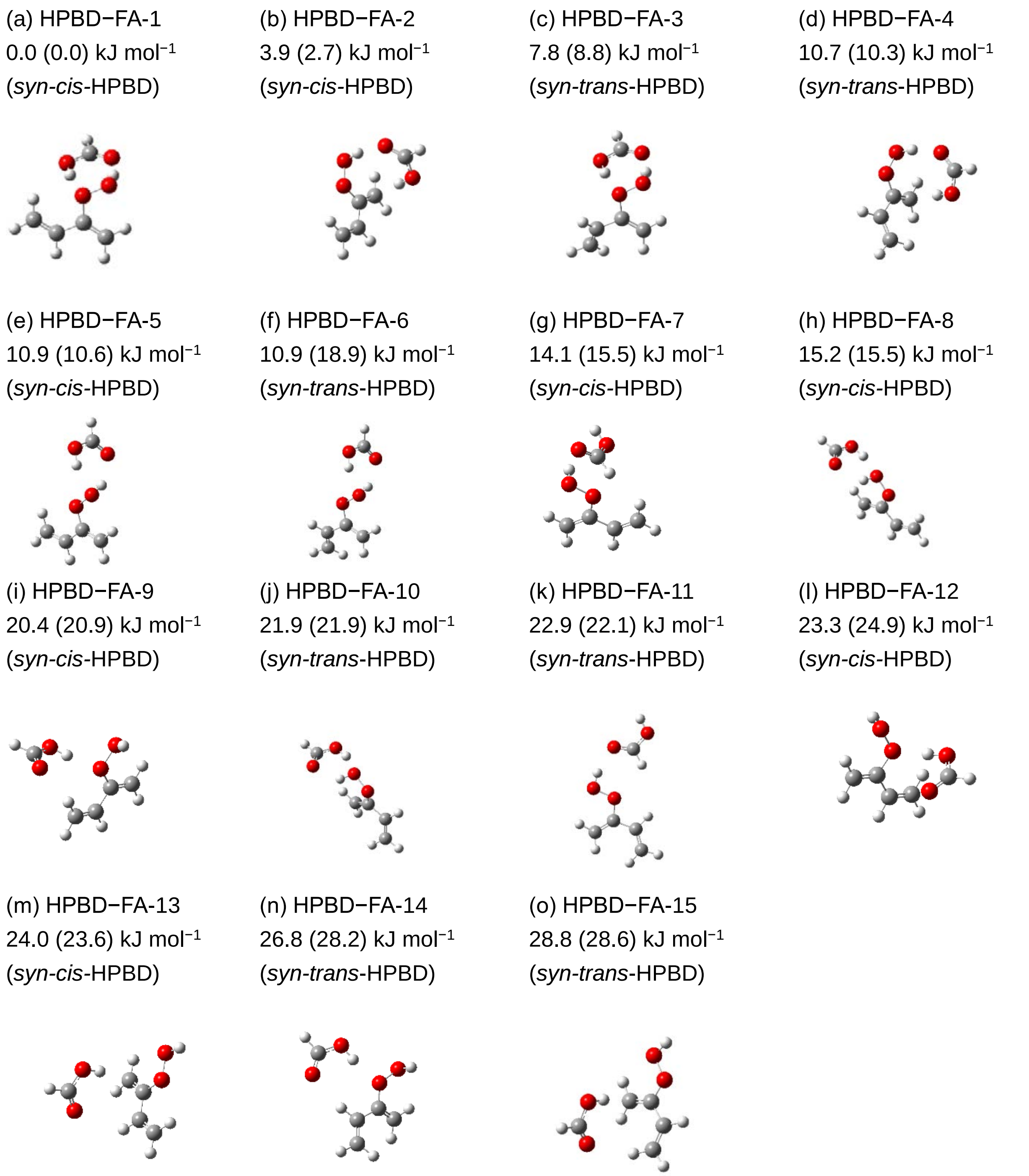

Figure S4. Geometries of fifteen conformers of complex of HPBD and HC(O)OH (HPBD−FA) calculated with the B3LYP+D3/aug-cc-pVTZ method. Relative energies were calculated with the CCSD(T)/augcc-pVTZ//B3LYP+D3/aug-cc-pVTZ method; those calculated with the B3LYP+D3/aug-cc-pVTZ method are listed in parentheses for comparison.

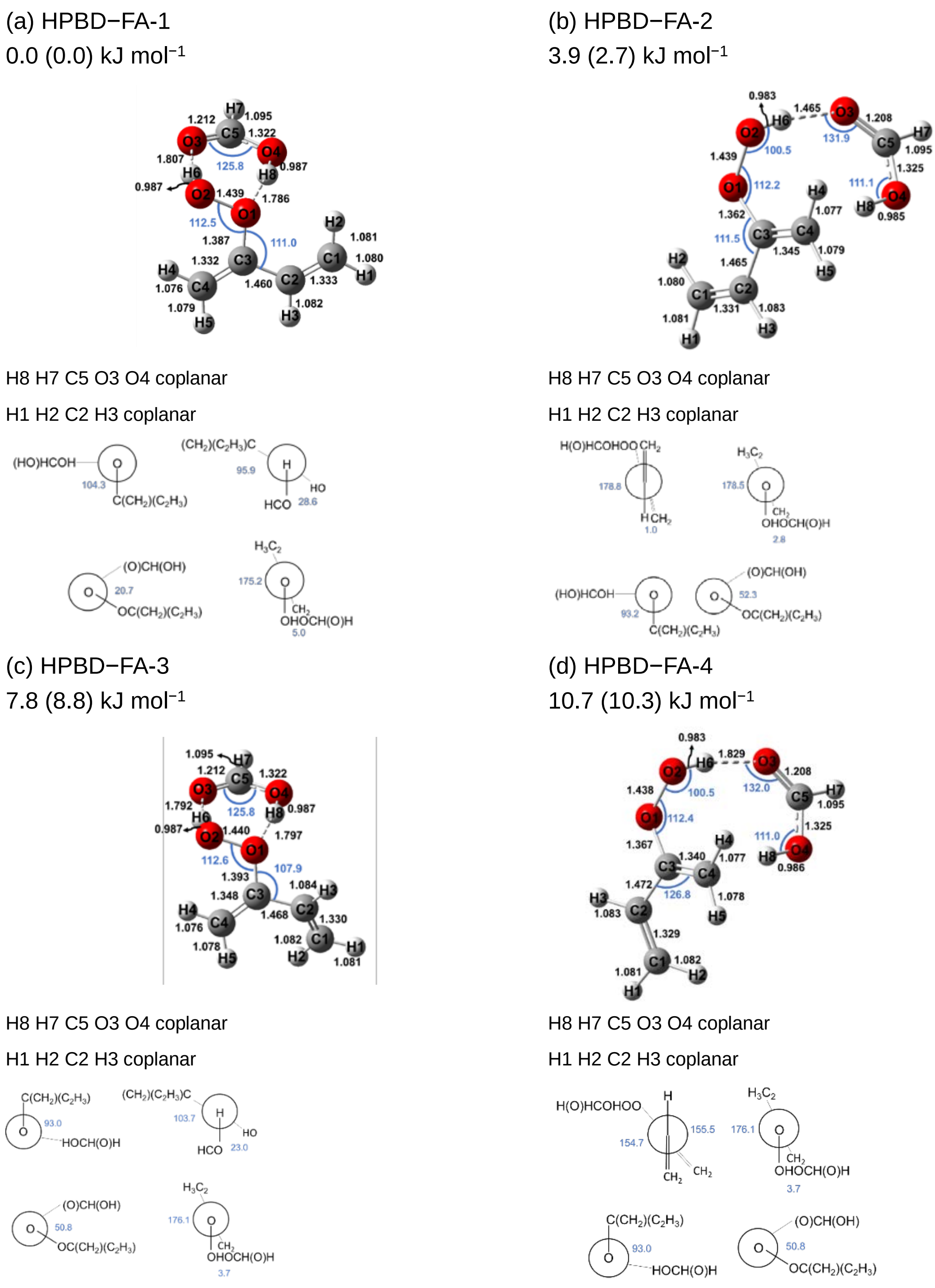

Figure S5. Geometries of four lowest-energy conformers of complex of HPBD and HC(O)OH (HPBD−FA) calculated with the B3LYP+D3/aug-cc-pVTZ method. (a) HPBD−FA-1, (b) HPBD−FA-2, (c) HPBD−FA-3, and (d) HPBD−FA-4. Bond lengths are in Å and bond angles in degrees. Relative energies were calculated with the CCSD(T)/aug-cc-pVTZ//B3LYP+D3/aug-cc-pVTZ method; those calculated with the B3LYP+D3/aug-cc-pVTZ method are listed in parentheses for comparison.

(a) *syn-trans*-MVKO…FA (PRC 1)

−63.7 (−59.0) kJ mol⁻¹

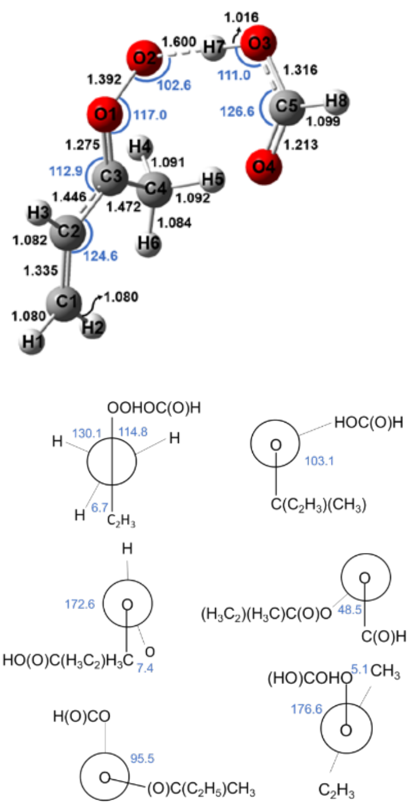

(b) *syn-cis*-MVKO…FA (PRC 2)

−57.2 (−52.8) kJ mol⁻¹

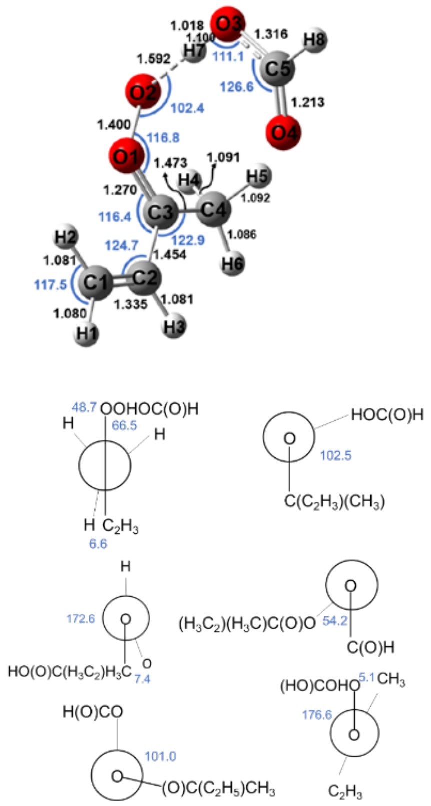

(c) TS1

−61.9 (−51.3) kJ mol⁻¹

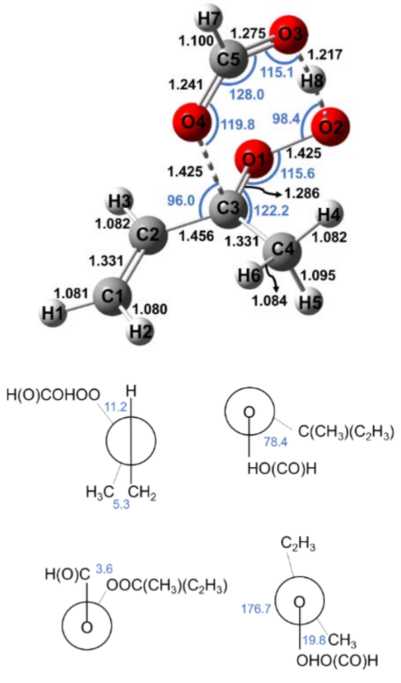

(d) TS2

−23.2 (−28.7) kJ mol⁻¹

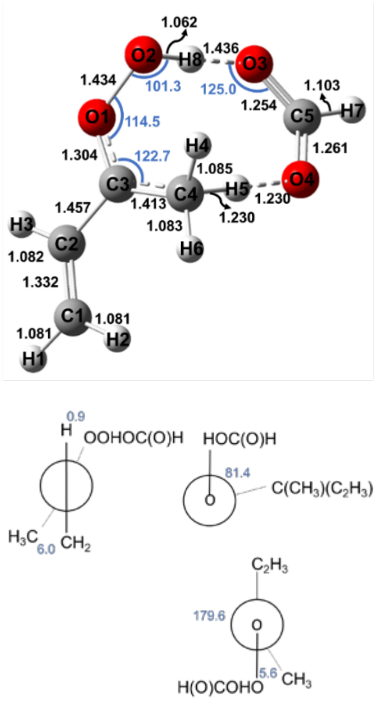

(e) TS3

–25.2 (–28.0) kJ mol⁻¹

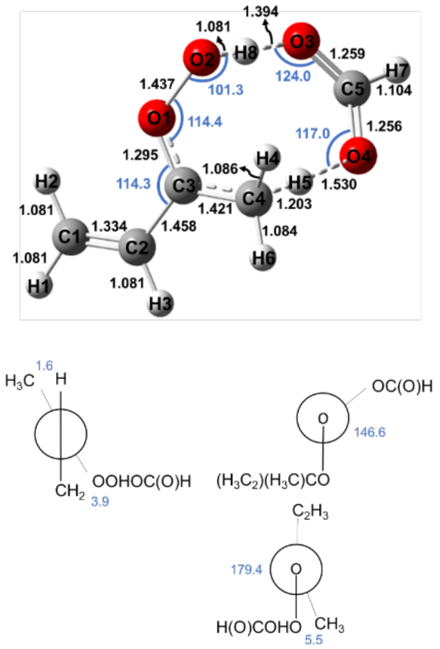

(f) TS4

72.6 (48.5) kJ mol⁻¹

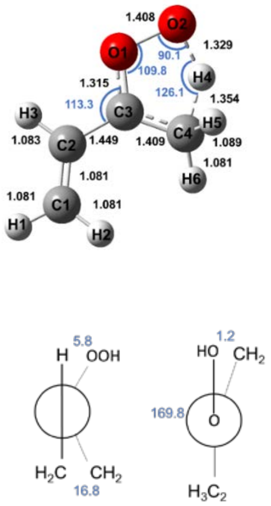

(g) TS5

77.8 (52.9) kJ mol⁻¹

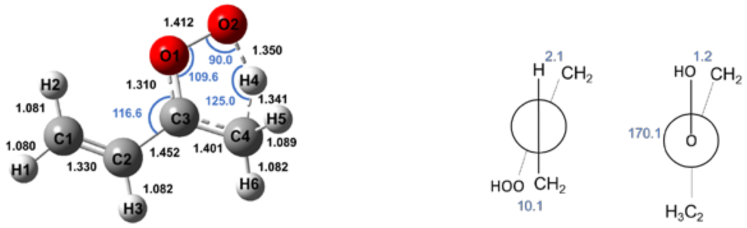

Figure S6. Geometries of two lowest-energy conformers of *syn*-*trans*-MVKO…FA (PRC1), *syn*-*cis*-MVKO…FA (PRC2), and transition state TS1−TS5 calculated with the B3LYP+D3/aug-cc-pVTZ method. (a) PRC1, (b) PRC2, (c) TS1, (d) TS2, (e) TS3, (f) TS4, and (g) TS5. Bond lengths are in Å and bond angles in degrees. Energies relative to *syn*-*trans*-MVKO + HC(O)OH were calculated with the CCSD(T)/aug-cc-pVTZ//B3LYP+D3/aug-cc-pVTZ method; those calculated with the B3LYP+D3/aug-cc-pVTZ method are listed in parentheses for comparison.

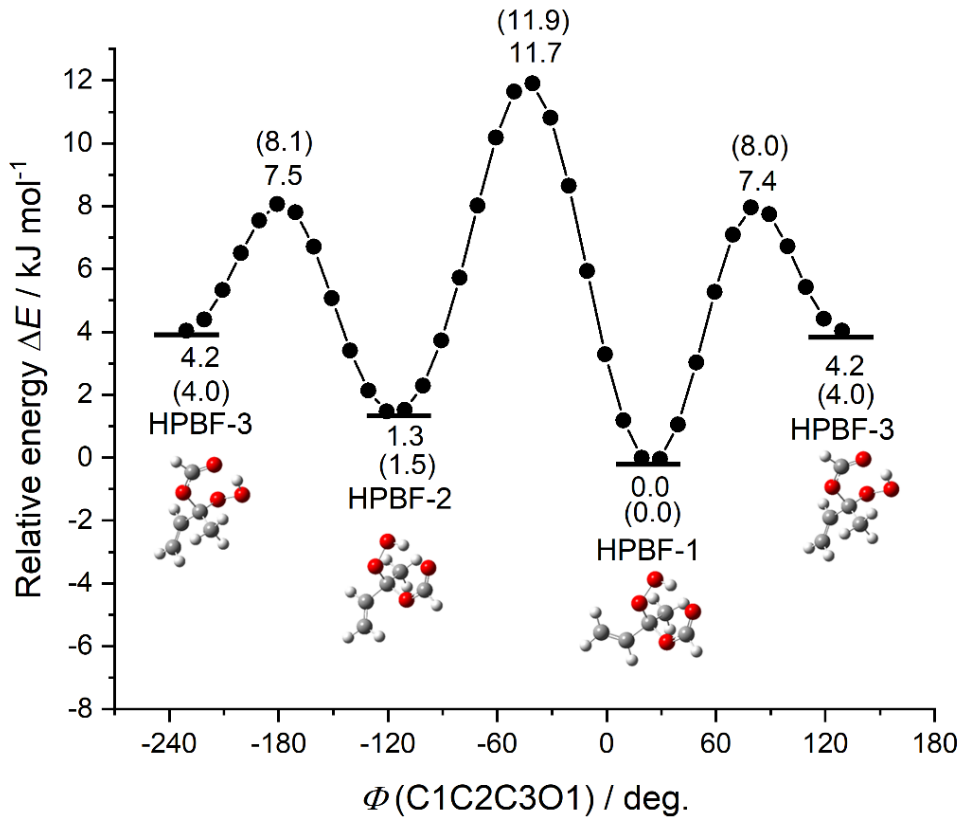

Figure S7. Potential energies for the interconversion of three lowest-energy conformers of HPBF calculated with the CCSD(T)/aug-cc-pVTZ//B3LYP+D3/aug-cc-pVTZ method. Those calculated with the B3LYP+D3/aug-cc-pVTZ method are listed in parentheses for comparison. The energy of HPBF-1 is set to zero.

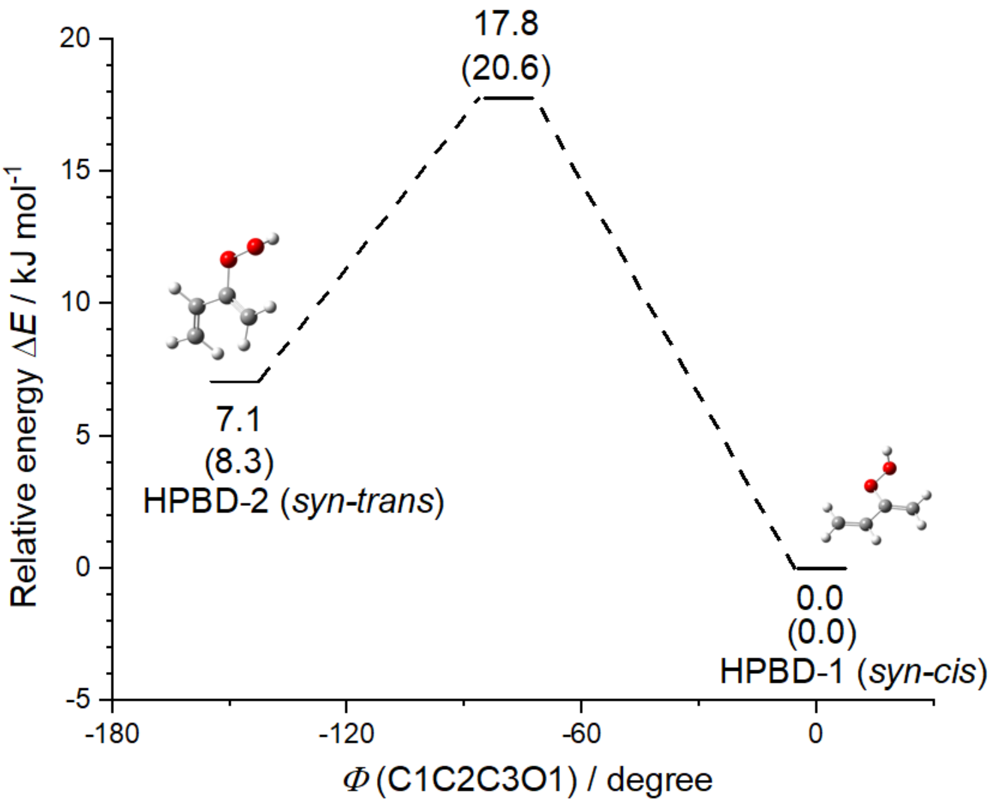

Figure S8. Potential energies for the interconversion of two *syn*-conformers of $C_2H_3C(=CH_2)OOH$ (HPBD) calculated with the CCSD(T)/aug-cc-pVTZ//B3LYP+D3/aug-cc-pVTZ method. Those calculated with the B3LYP+D3/aug-cc-pVTZ method are listed in parentheses for comparison. The energy of HPBD-1 is set to zero.

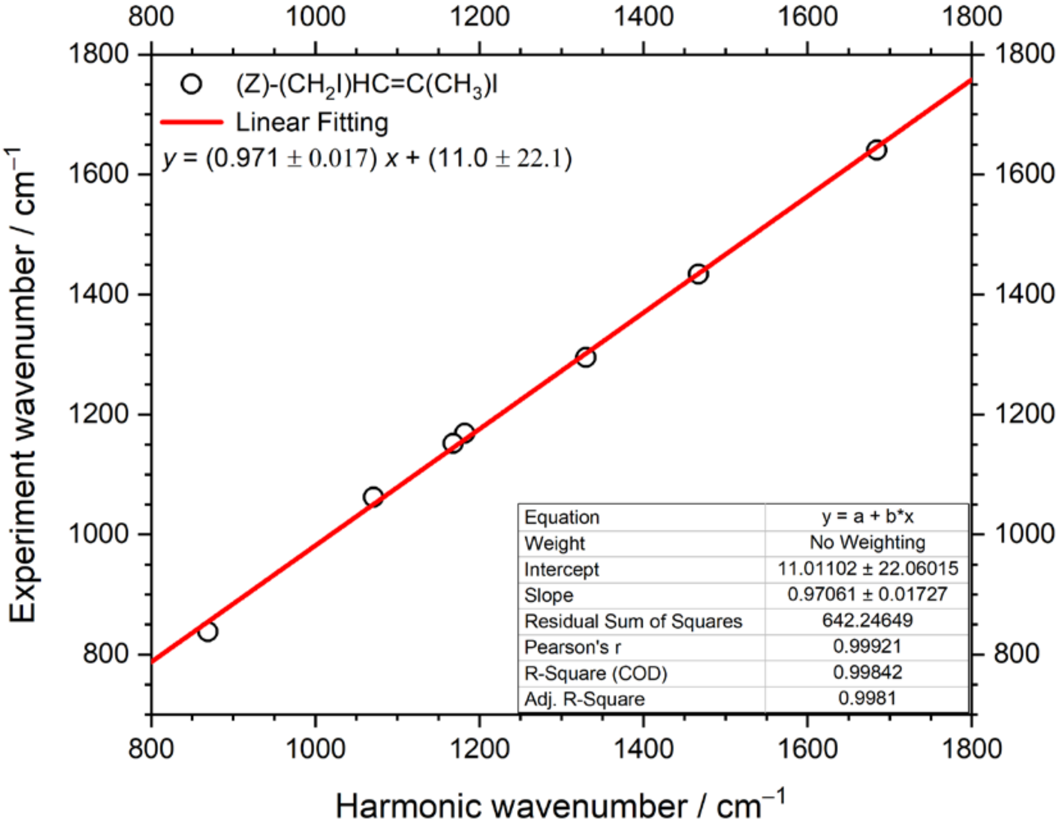

Figure S9. Comparison of experimentally observed wavenumbers with the harmonic vibrational wavenumbers of (*Z*)-(CH_2I)HC=C(CH_3)I predicted with the B3LYP /aug-cc-pVTZ-pp method.

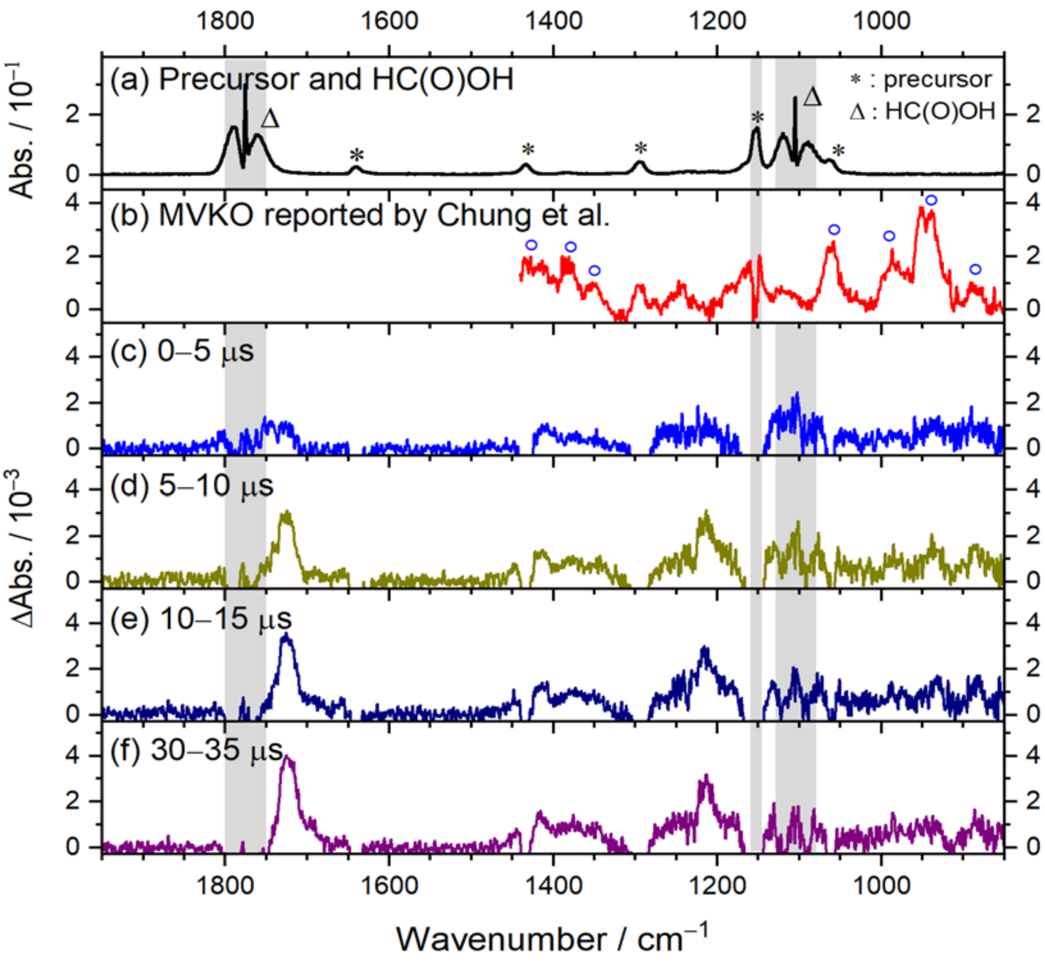

Figure S10. IR spectra recorded with an external ADC following photolysis at 248 nm of a flowing mixture of (*Z*)-(CH_2I)HC=C(CH_3)I/HC(O)OH/O_2 (0.025/0.038/40.0, P_T = 40.1 Torr) at 298 K. (a) Absorption spectrum before photolysis. Bands of (*Z*)-(CH_2I)HC=C(CH_3)I are indicated by *, while those of HC(O)OH by Δ. (b) MVKO absorption spectrum reported by Chung and Lee [*Commun. Chem.* **2021**, *4*, 8]. Difference spectra recorded 0−5 μs (c), 5−10 μs (d), 10−15 μs(e), and 30−35 μs (f) after irradiation; negative bands of precursor and HC(O)OH are truncated. Regions interfered with by absorption of precursors are shaded gray. Instrumental resolution is 1.0 cm^{-1}.

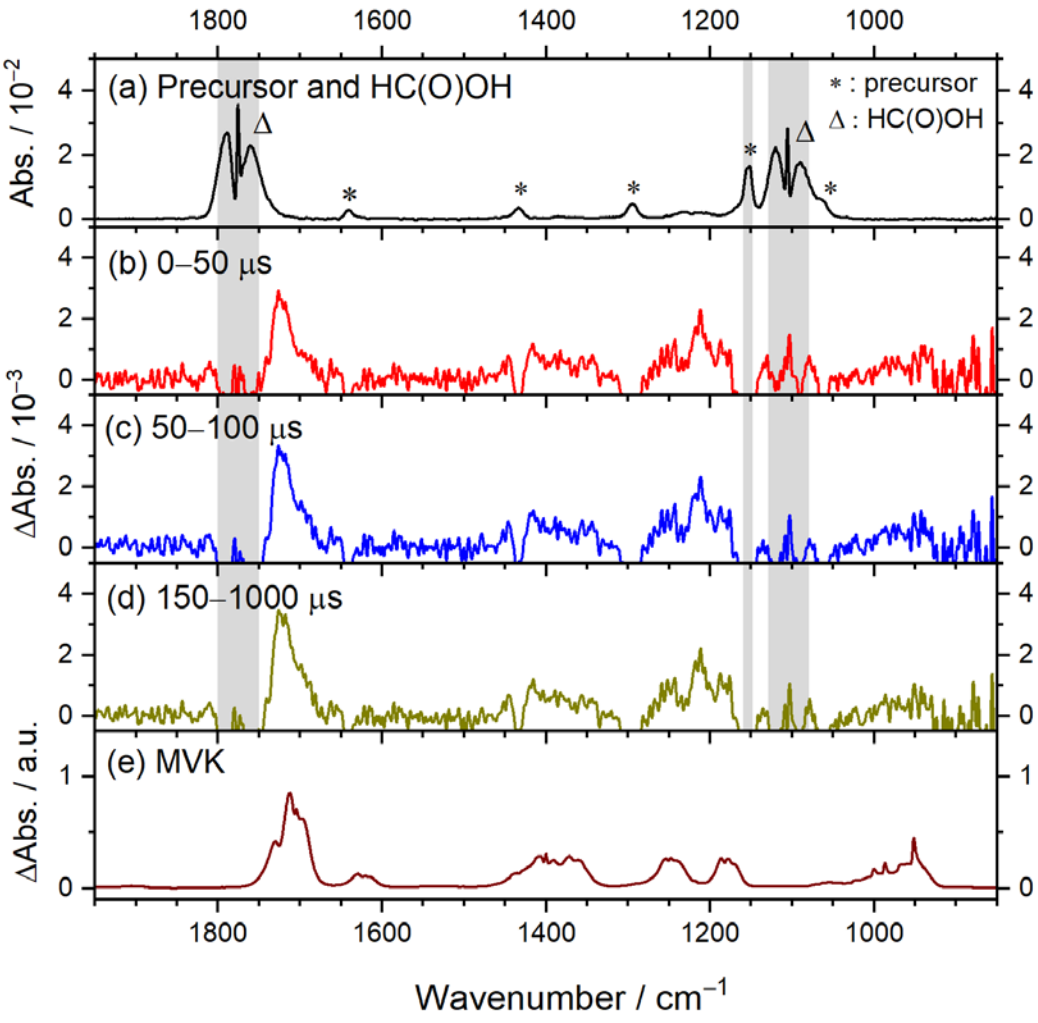

Figure S11. IR spectra recorded with an internal ADC following photolysis at 248 nm of a flowing mixture of (*Z*)-(CH_2I)HC=C(CH_3)I/HC(O)OH/O_2 (0.037/0.055/40.0, P_T = 40.1 Torr) at 298 K. (a) Absorption spectrum before photolysis. Difference spectra recorded 0−50 μs (b), 50−100 μs (c), and 150−1000 μs(d) after irradiation; negative bands of precursor and HC(O)OH are truncated. (e) IR absorption spectrum of methyl vinyl ketone (MVK). Regions interfered with by absorption of precursors are shaded gray. Instrumental resolution is 2.0 cm^{-1}.

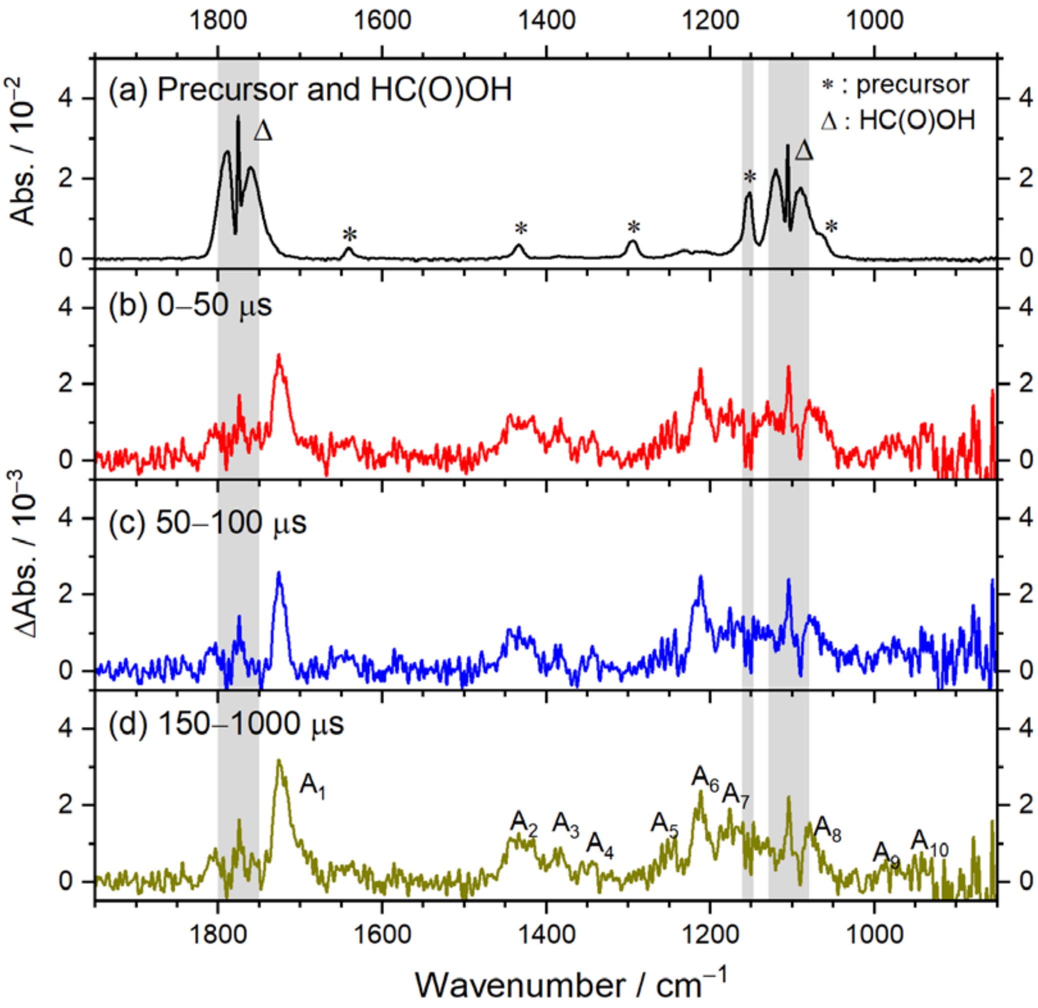

Figure S12. IR spectra recorded with an internal ADC following photolysis at 248 nm of a flowing mixture of (*Z*)-(CH_2I)HC=C(CH_3)I/HC(O)OH/O_2 (0.037/0.055/40.0, P_T = 40.1 Torr) at 298 K. (a) Absorption spectrum before photolysis. Processed difference spectra recorded 0−50 μs (b), 50−100 μs (c), and 150−1000 μs (d) after irradiation; depletion of absorption bands of the precursors (*Z*)-(CH_2I)HC=C(CH_3)I and HC(O)OH were added back and the contribution from MVK was subtracted. Regions interfered with by absorption of precursors are shaded gray. New features in groups A are labeled A_1−A_{10} in (f). Instrumental resolution is 2.0 cm^{-1}.

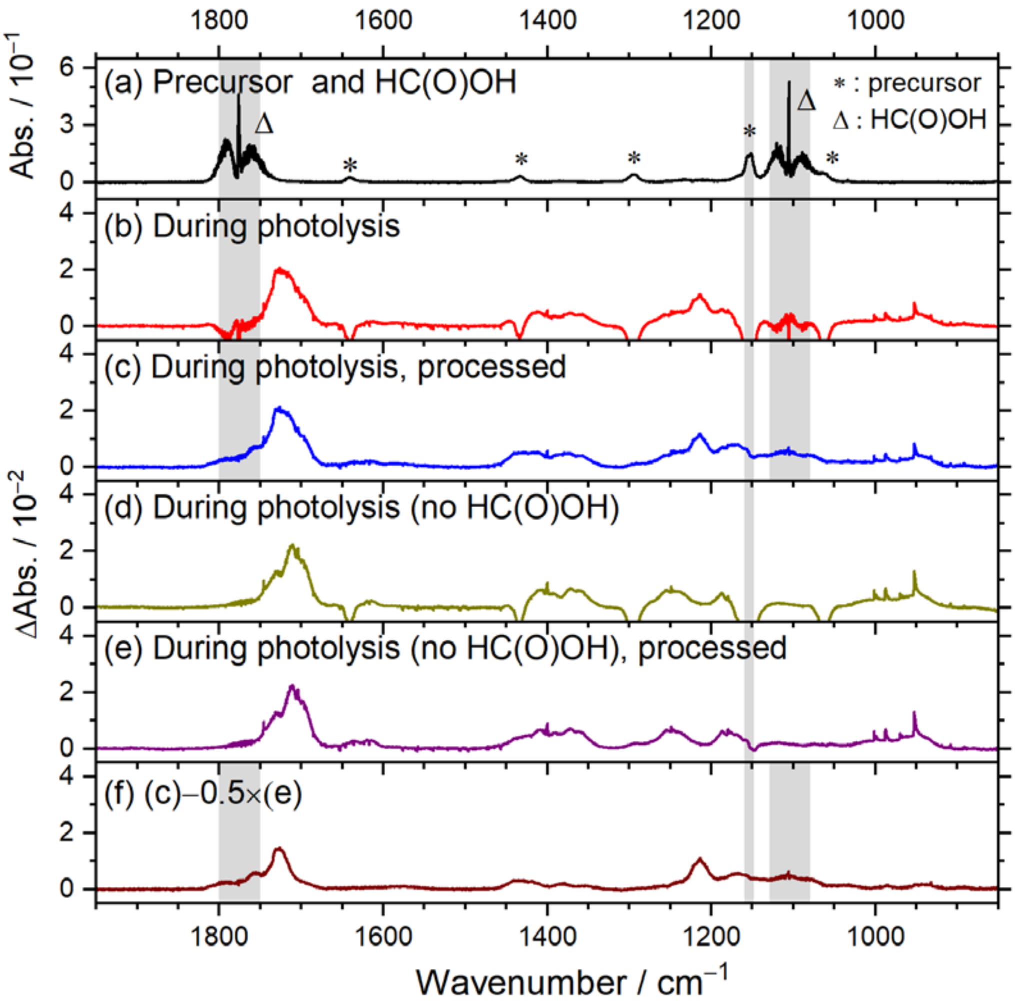

Figure S13. IR spectra recorded with continuous-scan mode during photolysis at 248 nm of a flowing mixture of (*Z*)-$(CH_2I)HC{=}C(CH_3)I/HC(O)OH/O_2$ at 40.8 Torr and 298 K. (a) Absorption spectrum before photolysis. (b) Difference spectra of a flowing mixture of (*Z*)-$(CH_2I)HC{=}C(CH_3)I/HC(O)OH/O_2$ (0.032/0.039/40.7, P_T = 40.8 Torr) during photolysis. (c) Processed spectra of (b); depletion of absorption bands of the precursors (*Z*)-$(CH_2I)HC{=}C(CH_3)I$ and HC(O)OH were added back. (d) Difference spectra of a flowing mixture of (*Z*)-$(CH_2I)HC{=}C(CH_3)I/O_2$ (0.032/40.7, P_T = 40.7 Torr) during photolysis. (e) Processed spectra of (d); depletion of absorption bands of the precursors (*Z*)-$(CH_2I)HC{=}C(CH_3)I$ and HC(O)OH were added back. (f) Spectrum (c) minus 0.5 times spectrum (e). Regions interfered with by absorption of precursors are shaded gray. Instrumental resolution is 0.25 cm^{-1}.

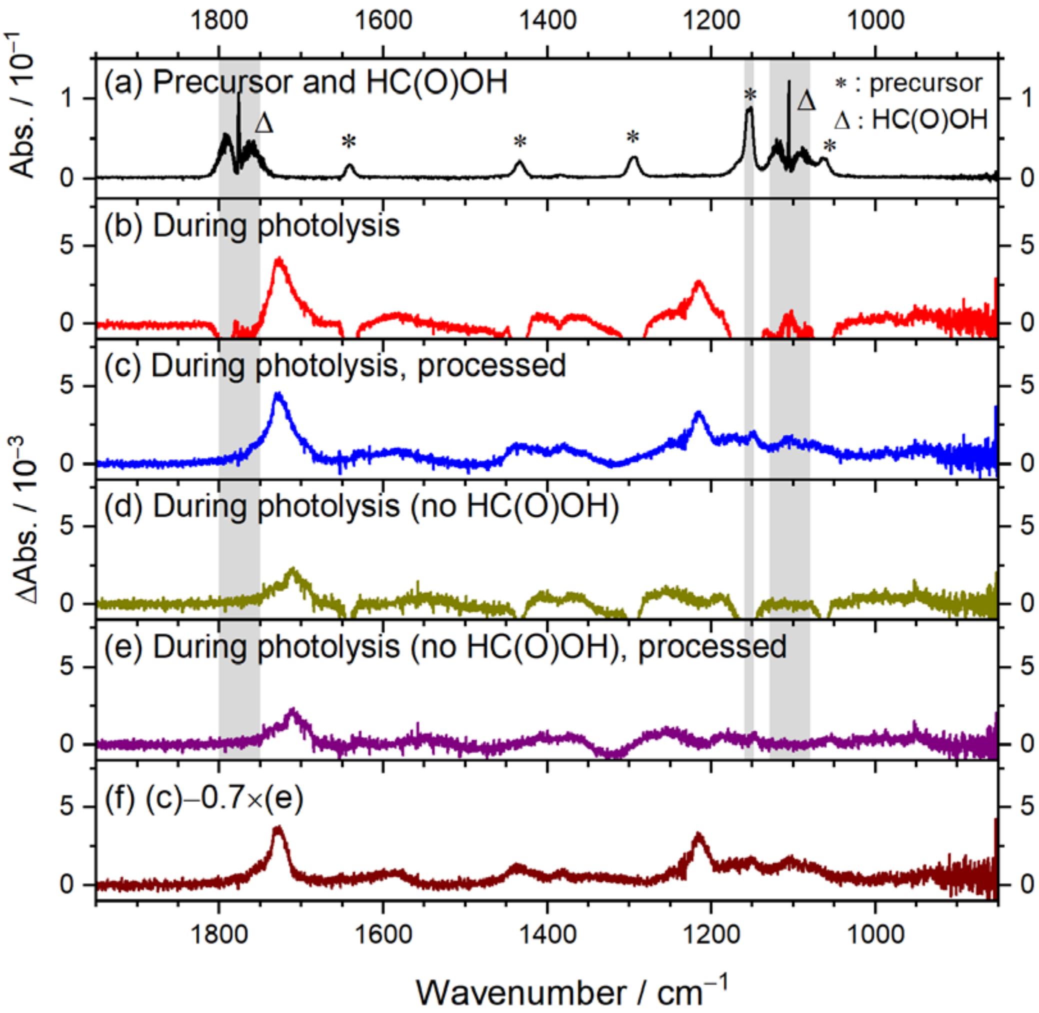

Figure S14. IR spectra recorded with continuous-scan mode during photolysis at 248 nm of a flowing mixture of (Z)-(CH_2I)HC=C(CH_3)I/HC(O)OH/O_2 at 10.7 Torr and 298 K. (a) Absorption spectrum before photolysis. (b) Difference spectra of a flowing mixture of (Z)-(CH_2I)HC=C(CH_3)I/HC(O)OH/O_2 (0.015/0.011/10.7, P_T = 10.7 Torr) during photolysis. (c) Processed spectra of (b); depletion of absorption bands of the precursors (Z)-(CH_2I)HC=C(CH_3)I and HC(O)OH were added back. (d) Difference spectra of a flowing mixture of (Z)-(CH_2I)HC=C(CH_3)I/O_2 (0.015/10.7, P_T = 10.7 Torr) during photolysis. (e) Processed spectra of (d); depletion of absorption bands of the precursors (Z)-(CH_2I)HC=C(CH_3)I and HC(O)OH were added back. (f) Spectrum (c) minus 0.5 times spectrum (e). Regions interfered with by absorption of precursors are shaded gray. Instrumental resolution is 0.25 cm^{-1}.

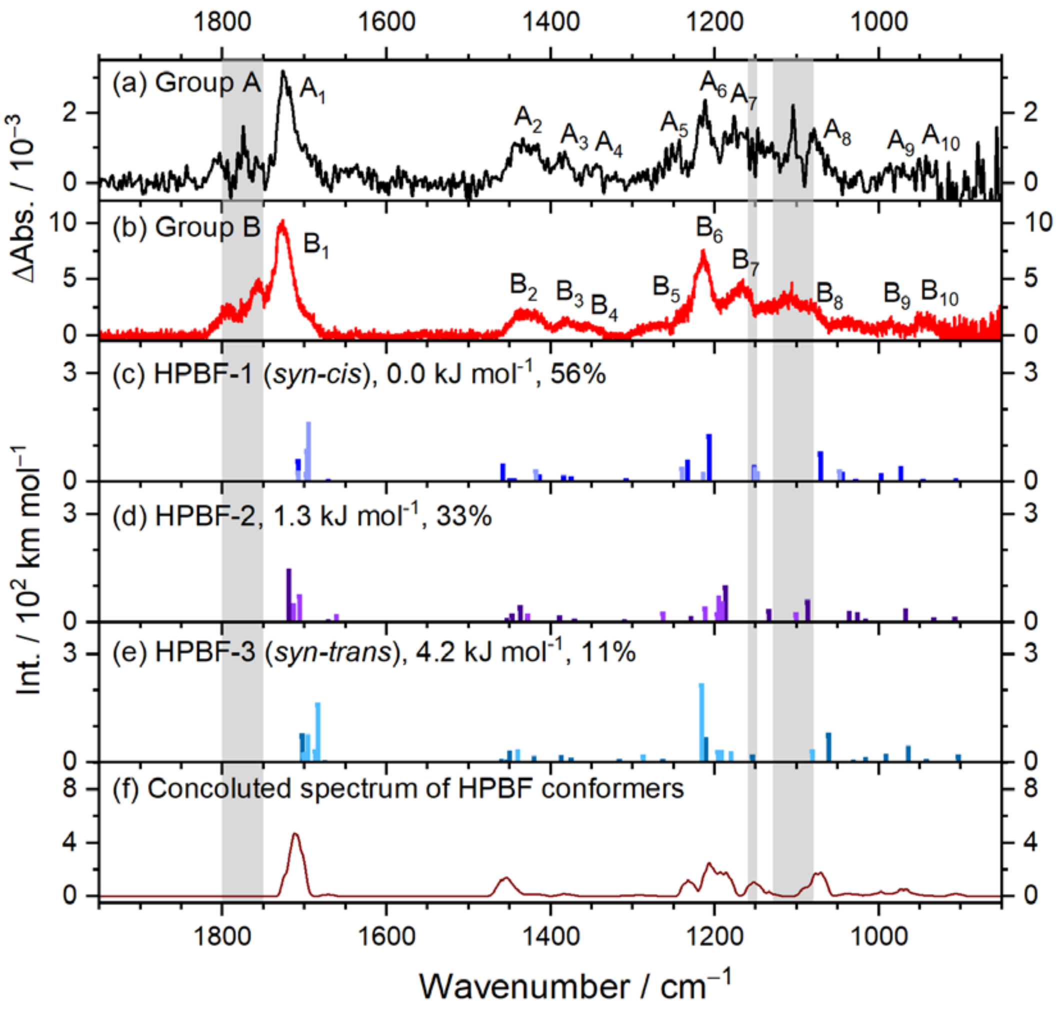

Figure S15. Comparison of bands in groups A and B with simulated anharmonic spectra of three lowest-energy conformers of HPBF, $(C_2H_3)C(CH_3)(OCHO)OOH$. (a) Absorption spectrum of group A, taken from Figure S12d with instrumental resolution 2 cm^{-1}. (b) Absorption spectrum of group B, taken from Figure 5d with instrumental resolution 0.25 cm^{-1}. Simulated spectra of HPBF-1 (c), HPBF-2 (d), and HPBF-3 (e) based on anharmonic vibrational wavenumbers and IR intensities predicted with the B3LYP+D3/aug-cc-pVTZ method; overtone and combination bands are presented in light colors. (f) Convoluted spectrum of HPBF-1 to HPBF-3 according to Boltzmann population distribution 0.58 : 0.33 : 0.11. Gaussian FWHM is 5 cm^{-1}. Regions interfered with by absorption of the precursors are shaded gray.

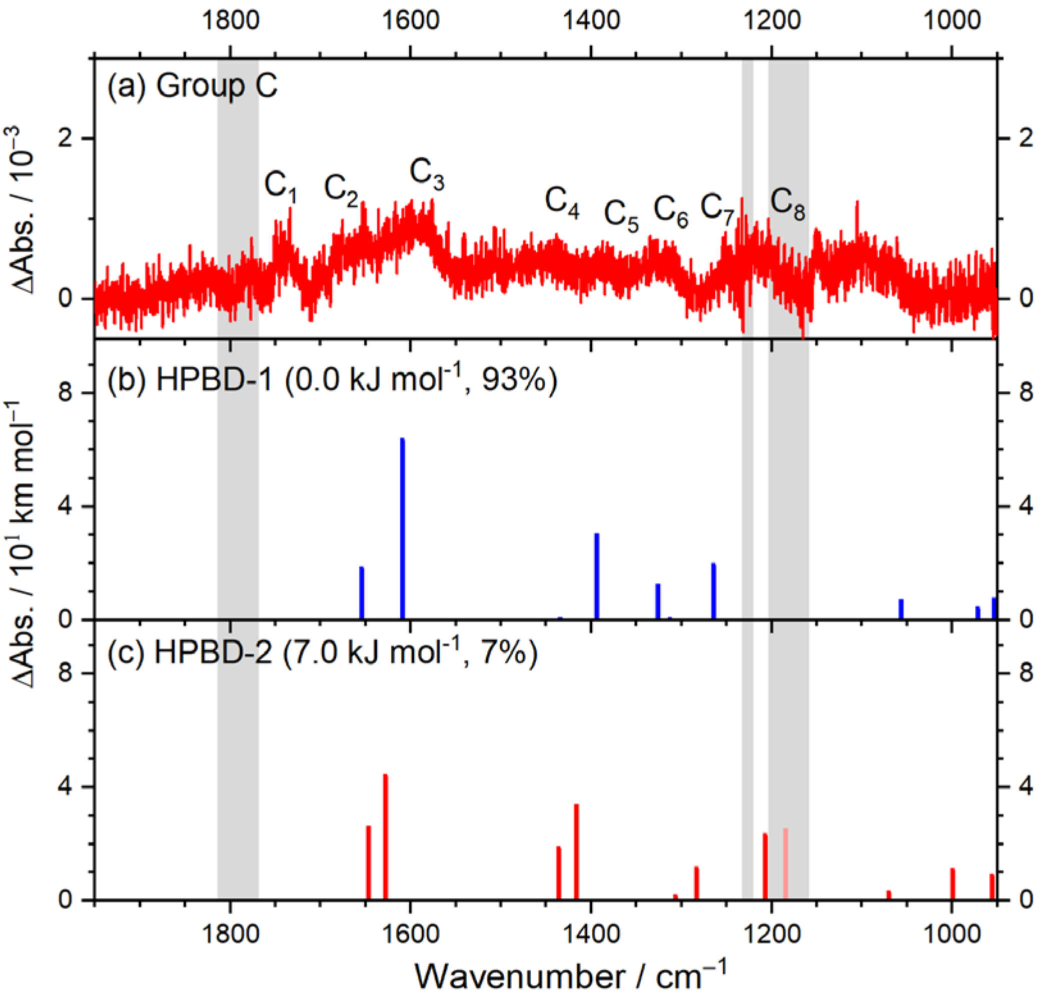

Figure S16. Comparison of bands in group C with stick anharmonic spectra of two conformers of HPBD, $C_2H_3C(=CH_2)OOH$. (a) Absorption spectrum of group C taken from Figure 5e. IR stick spectra of HPBD−1 (b) and HPBD−2 (c) based on anharmonic vibrational wavenumbers and IR intensities predicted with the B3LYP+D3/aug-cc-pVTZ method; overtone and combination bands are presented in light colors. Region interfered with by absorption of the precursors are shaded gray.
